# Abstracts of the 14th C1-inhibitor Deficiency and Angioedema Workshop

**DOI:** 10.1186/s13223-025-00992-1

**Published:** 2026-01-03

## Preface

We are pleased to welcome all participants to the **14th C1-inhibitor Deficiency & Angioedema Workshop**.

The aim of the Workshop is to present new research findings related to rare bradykinin-mediated angioedema disorders. These include conditions caused by hereditary or acquired C1-inhibitor deficiency, as well as those with a hereditary background but normal C1-inhibitor levels. This year, a record number of abstracts will be presented over the four-day program, including 49 oral and 58 poster presentations. In addition to previously unpublished findings, five outstanding keynote lectures will also be delivered.

On the opening afternoon, Nobel Laureate Katalin Karikó will give a special lecture on the development of the mRNA vaccine, sharing all the insights gained from the long and persistent journey that led to the production of life-saving mRNA-based vaccines.

The upcoming days of the program are also filled with a wealth of engaging topics. Of theoretical significance is the presentation by Stephen Thiel on ITIH4 (Inter-α-trypsin inhibitor heavy chain 4), a regulator of plasma proteolytic systems. ITIH4 may play a regulatory role in the complement-kallikrein system, especially under conditions where C1-inhibitor is absent or dysfunctional.

Monoclonal antibody-based therapies are increasingly used to prevent symptoms in angioedema disorders. However, the nature of these antibodies, including how they are dispersed, processed, and degraded within the body, is rarely discussed among prescribing specialists. László Cervenak will provide a comprehensive overview on this topic.

As Anastasios Germenis will highlight, the diagnosis of specific types of HAE with normal C1-inhibitor (HAE-nC1-INH) can only be confirmed by identifying a known pathogenic variant in one of the genes associated with the disease. His presentation will offer guidance on how to interpret and apply genetic results.

Bruce Zuraw will summarize key clinical considerations that practicing physicians must keep in mind when prescribing preventive therapies developed through clinical trials.

The Workshop also serves as an ideal platform to develop international consensus documents or guidelines based on thoroughly prepared collaborative work. This year, we will engage participants in developing professional recommendations for the modern management of hereditary angioedema in children.

The 14th C1-inhibitor Deficiency & Angioedema Workshop is also a venue for celebration. It is a tradition to honor a distinguished professional with the ‘For HAE Patients’ Award, recognizing outstanding contributions to improving the lives of patients.

This year’s honoree is Laurence Bouillet, Clinical Director of the Internal Medicine/Clinical Immunology Unit at the University of Grenoble and Vice President of the French National Society of Internal Medicine. Throughout her career, she has gained international recognition not only for her research but also for leading France’s National Reference Center for Angioedema (CREAK).

A posthumous ‘For HAE Patients’ Award is presented in memory of Marcus Maurer, who passed away unexpectedly in 2024. He was Professor of Dermatology and Allergy, and Executive Director of the Institute of Allergology at Charité – Universitätsmedizin Berlin. His pioneering work, though cut short, created a lasting legacy, and his colleagues and students continue his initiatives.

On the final day of the Workshop, we will recognize outstanding young researchers. An independent jury chaired by Stephen Betschel will award the ‘Grant for Young Investigator’ and the accompanying financial prize to the four best oral and poster presenters.

To help navigate the vast amount of information shared during the four days, Avner Reshef will provide a focused overview of the most significant messages intended to serve as key takeaways for the audience.

To ensure lasting access to the content, all abstracts will be published in complete form in a special issue of the open-access journal Allergy, Asthma and Immunology, available later this year.

Many of our sponsors will be represented at the Workshop in various forms: BioCryst, CSL Behring, Kalvista, Takeda, Otsuka, Pharming, Pharvaris, Astria, Intellia. Their generous support enables us to provide a high-quality environment for productive work and helps fund both the ‘For HAE Patients’ Awards and the ‘Grant for Young Investigator’ prizes. We are sincerely grateful for their contributions.

The Workshop is organized by our professional partner, Diamond Congress Ltd. We are thankful for their smooth and personalized service.

**Henriette Farkas**

Chair of the 14^th^ C1-inhibitor Deficiency and Angioedema Workshop

## I-01 Developing mRNA for therapy: My journey

### Katalin Karikó, PhD

#### Professor, University of Szeged, Hungary; Adjunct Professor, University of Pennsylvania Perelman School of Medicine, USA

***Allergy, Asthma & Clinical Immunology*** 2025, **21(Suppl 2)**:I-01

Messenger RNA was discovered in 1961 and it took 60 years until the first mRNA became FDA-approved product in the form of COVID-19 mRNA vaccine. During those years a lot of progress has been made by hundreds of scientists. First, isolated mRNAs were structurally and functionally characterized. In 1978 isolated mRNA delivered into mammalian cells were shown to produce the encoded protein. In vitro transcription—introduced in 1984—made it possible to generate mRNA coding for any desired protein from the corresponding DNA by using phage RNA polymerases. In the early 90 s in vitro-transcribed mRNA was mainly tested in animals as vaccine against infectious diseases and cancer. A great extent of progress toward a viable treatment was made during those years but the inflammatory nature of mRNA initially hampered its medical use. Together with my colleagues, we achieved a great milestone when we warded off the response by replacing uridine with pseudouridine in mRNA. We further demonstrated that nucleoside-modified mRNA formulated with lipid nanoparticles can be a potent vaccine. These discoveries eventually led to the development of the mRNA vaccine that has now helped to fight the global pandemic and opened the door for developing breakthrough therapeutics for incurable diseases and unmet medical needs.

## I-02 The protease inhibitor Inter-α-trypsin inhibitor heavy chain 4 (ITIH4) as a compensatory inhibitor in plasma of hereditary angioedema patients

### Steffen Thiel

#### Department of Biomedicine, Aarhus University, Denmark

***Allergy, Asthma & Clinical Immunology*** 2025, **21(Suppl 2)**:I-02

Inter-α-trypsin inhibitor heavy chain 4 (ITIH4) is a regulator of plasma proteolytic systems, with growing evidence linking it to both health and disease. Synthesized predominantly in the liver, ITIH4 circulates at concentrations of approximately 200 µg/mL, underscoring its abundance within the bloodstream. We have recently revealed a pivotal role for ITIH4 in modulating inflammatory pathways, particularly the complement and kallikrein-kinin systems. Upon encountering proteases—such as kallikrein or activated components of the complement cascade—ITIH4 undergoes proteolytic cleavage, forming complexes with the active enzyme that can effectively neutralize or diminish enzymatic activity. This function positions ITIH4 as a potential “backup” protease inhibitor, especially significant in conditions where classical regulators (e.g., C1-inhibitor) are deficient or dysfunctional. The resulting alterations in ITIH4 isoforms or cleavage patterns have been observed in various pathologies, making it an intriguing candidate biomarker for inflammatory diseases such as hereditary angioedema (HAE). We have recently identified ITIH4 as a potential compensatory protease inhibitor in HAE. Our findings revealed that ITIH4 is cleaved during HAE attacks, depleting intact ITIH4 in plasma. This indicates that ITIH4 may serve as a biomarker in HAE.

Ongoing research aims to clarify how these changes may predict clinical severity, guide therapeutic interventions, and advance personalized medicine. Collectively, these findings highlight ITIH4’s capacity to modulate proteolytic homeostasis, offering novel insights into disease mechanisms and opportunities for targeted diagnostic and treatment strategies.

## I-03 Incidental findings related to genes associated to HAE-nC1-INH: How to proceed?

### Anastasios E. Germenis

#### Department of Immunology & Histocompatibility, School of Medicine, University of Thessaly, Larissa, Greece

***Allergy, Asthma & Clinical Immunology*** 2025, **21(Suppl 2)**:I-03

The diagnosis of the specific type of HAE-nC1-INH can only be confirmed by the identification of a known pathogenic variant in someone of the seven genes linked to the disease (F12, PLG, ANGPT1, KNG1, MYOF, HS3ST6 or DAB2IP). In view of recent advances in genomic technologies, targeted panels currently used to this purpose will be replaced by exome or genome sequencing. Consequently, incidental finding of VUS in these genes is becoming increasingly probable. In addition, the widespread use of large-scale sequencing in clinical settings, which is increasing the frequency of VUS detection across the genome, is also enabling the incidental finding of HAE-nC1-INH pathogenic variants.

The incidental finding of HAE-nC1-INH causal variants confers the chance to identify and manage a type of HAE-nC1-INH that may otherwise be unrecognized in an individual. Clinical actionability (low burden and risk, availability, and effectiveness of interventions) of these variants justifies their reporting to clinicians. However, it is important to note that incidental findings cannot be considered in the same manner as genotyping results in a diagnostic setting, as they represent a form of screening. For this reason, clinicians should implement evaluation and management approaches that accounts for both the increased disease risk associated with an incidental finding and the lower positive predictive value of a screening result compared to an indication-based testing result. Moreover, clinical evaluation of these findings must take into account the incomplete penetrance demonstrated by the HAE-nC1-INH as well as the fact that, in the setting of an incidental finding, the penetrance may be somewhat decreased compared to penetrance observed from family ascertainment-based studies.

A variant may be classified as a VUS when: (a) there is insufficient evidence to support a more definitive classification of the variant as either likely pathogenic or benign, despite being identified in a gene known to be related to HAE-nC1-INH; (b) it is identified in a gene of uncertain significance but the nature of the variant suggests it may be causative of angioedema phenotype (i.e. truncating variant); (c) a variant lacking evidence of pathogenicity is identified in a known disease causing gene unrelated to angioedema phenotype. In any case, VUS should not be considered as sufficient grounds for clinical decision making. Therefore, VUS should only be considered for reporting to clinicians when there is a high level of supporting evidence and additional evidence might be obtained to allow reclassification as (likely) pathogenic (e.g. a VUS in exon 9 of F12 gene in a HAE-UNK patient). Considering that patients have the right to opt out of unintentional incidental findings, clinicians have pivotal roles in the ongoing re-evaluation and reclassification of VUS. This process mainly involves investigating the possible existence of angioedema phenotype in the family, facilitating the genetic family study to uncover family segregation, following the proband and the family, and reporting in public databases (e.g. ClinVar).

## I-04 Body vs. Antibody – Behavior of monoclonal therapeutic antibodies in angioedemas

### László Cervenak

#### Department of Internal Medicine and Haematology, Semmelweis University, Budapest, Hungary

##### **Correspondence:** László Cervenak (cervenak.laszlo@semmelweis.hu)

***Allergy, Asthma & Clinical Immunology*** 2025, **21(Suppl 2)**:I-04

Monoclonal antibodies are nowadays the most utilized form of large-molecule drugs. It is well-known what the different antibodies do with their target molecules/cells in the body, however, this reaction is always a two-way process. How the body disperses, processes, and degrades the monoclonal antibodies is much less taken into account.

Antibodies as free molecules and in complex with antigens have completely different roles, routes and kinetics in the body. It implies that antibody specificity itself strongly influences these parameters. In this review, however, the focus will be on the specificity independent properties of antibodies. The presence and quality of the Fc part of the antibody determines its clearance by tissue macrophages, neutrophils, Kupfer cells, spleen macrophages and other cell types with phagocytic activity when it is in complex with antigen, as well as the neonatal Fc-receptor/endothelial cell dependent plasma half-life of the free form. Technology behind the production of a monoclonal antibody (i.e. hybridoma vs. phage library methods; mouse, recombinant, humanized or human origin; bispecific, single-chain or conjugated) also influences the half-life, biodistribution, degradation, as well as the immunogenicity.

Even human therapeutic monoclonals antibodies (such as Lanadelumab and garadacimab, both of the two authorized therapeutic monoclonal antibody with hereditary angioedema indication) are potentially immunogenic, since they contain unique amino-acid sequences in their antigen-binding hypervariable region (CDR) in high dose. Moreover, monoclonal antibody therapy for life-long prevention is a higher risk for immunization compared to short term applications. Immunization may occur years after the first encounter with an antigen, thus, although only a few patients were found with neutralizing antibody against Lanadelumab so far, regular monitoring for anti-drug antibody is highly recommended.

Therapeutic monoclonal antibodies, as they are proven to recognize a clinically important target structure, can be utilized to help designing small molecular drugs for the very same target, which may avoid those drawbacks that limits the application of the antibodies. Besides antibody-driven design of small drugs would reduce the costs dramatically, it would enable the oral administration.

Understanding the behavior of therapeutic monoclonal antibodies is substantially important to plan, follow-up, and – if necessary – modify the therapeutic protocol in angioedemas.

## I-05 Assessment of risk and benefit in clinical trials: Challenges in rare diseases

### Bruce Zuraw

#### University of California, San Diego, CA, USA

***Allergy, Asthma & Clinical Immunology*** 2025, **21(Suppl 2)**:I-05

The ethical principles underlying clinical research developed over the past 80 years are well-established and universally accepted. The application of these principles, however, may not always be as clear-cut as desired—particularly in the case of a rare disease where there are unavoidable limits on our scientific knowledge. In such instances, investigators must assume a greater responsibility to independently assess the risk benefit ratio, and then accurately convey this to potential subjects to obtain true informed consent. This talk will focus on ongoing clinical trials of two drugs for the long-term prophylaxis of hereditary angioedema patients where potential concerns about the risk to benefit ratio present challenging questions. In one instance, these concerns are based on animal data and in the other instance on incomplete data from human patients. Understanding the importance of epistemological limits in assessing risk can assist investigators in protecting the interests of HAE patients during clinical research.
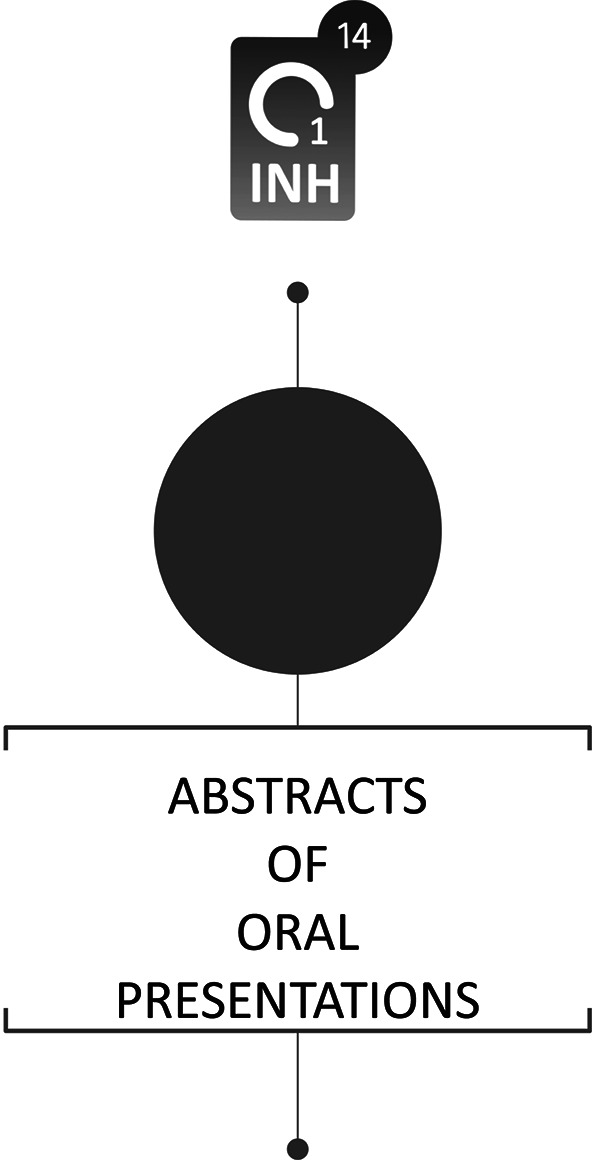


## O-01 Non-peptide bradykinin B2 receptor ligands possessing a quinolinyl moiety, or variants thereof: Pharmacological properties and computational prediction of binding modes

### François Marceau*, Ahmed Sahli, René C.-Gaudreault

#### Centre de recherche du CHU de Québec-Université Laval, Québec, Canada

##### **Correspondence:** François Marceau (francois.marceau@crchudequebec.ulaval.ca)

***Allergy, Asthma & Clinical Immunology*** 2025, **21(Suppl 2)**:O-01

Bradykinin (BK) is a nonapeptide derived from the cleavage of circulating kininogens by kallikreins and is endowed with powerful pharmacologic actions, such as the production of protein rich exudates and vasodilation. The widely expressed B2 receptor for BK (B2R, a G protein coupled receptor) has been the focus of intense drug development efforts for more than 4 decades, with marked differences in affinities and competitiveness for synthetic antagonists across mammalian species. A recurring substituted 8-[(2,6-dichlorophenyl)methoxy]-2-methylquinolinyl ("quinolyl") moiety, or variants thereof, has been explored by several pharmas (Fig. 1). FR173657, fasitibant, anatibant, deucrictibant and Compound 3 (the non-deuterated version of deucrictibant) are examples of competitive antagonists of the human B2R, some of which having reached the stage of clinical trials. Other compounds structurally related to the common moiety, such as FR190997 and Compound 47a, are partial or nearly full agonists of the B2R that are remarkably long acting. The recently disclosed structure of the human B2R obtained using cryo-electron microscopy, with or without coupling to the G_q_ protein, has been exploited in a computational drug binding study. Antagonist drugs of the quinolyl series are positioned in a buried cavity surrounded by the transmembrane domains. Their predicted affinities are in good agreement with pharmacology (notably, with antagonism of BK-induced contractility of the human isolated umbilical vein). Nonpeptide partial agonists rather bind to a different superficial cavity overlapping the BK binding site.

**Figure 1 (abstract O-01):**
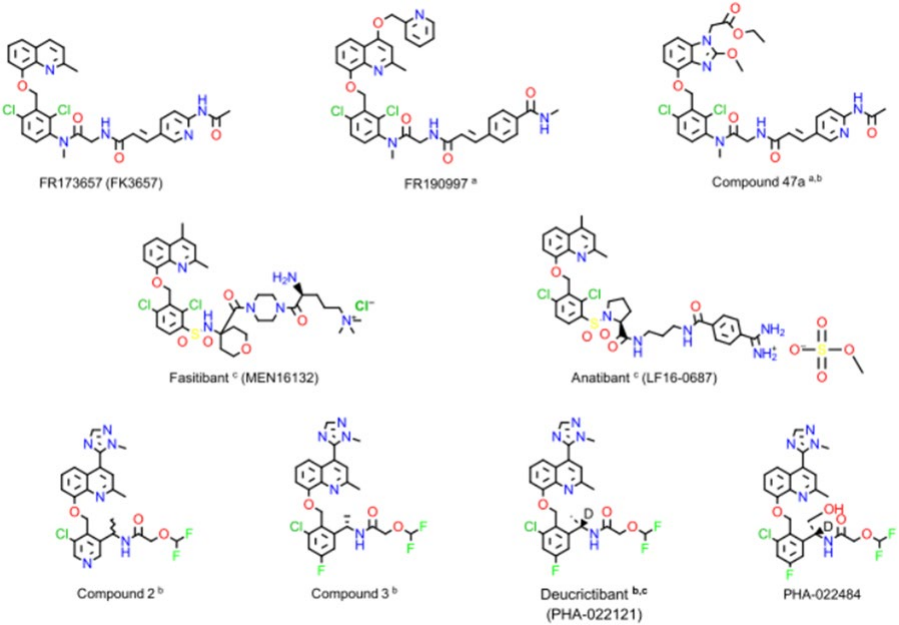
Structure of selected quinolyl ligands of the human B2R

Supported by Fondation du CHU de Québec and Génome Québec.

## O-02 Clinical validation of a novel biomarker assay to characterise bradykinin-mediated angioedema in prospective and biobank plasma samples

### Evangelia Pardali^1^, Hanga Réka Horváth^2^, Oliver Domenig^3^, Dunja van Oyen^3^, Dan Sexton^4^, Grit Zahn^5^, Anne Lesage^6^, Henriette Farkas^2^

#### ^1^Pharvaris B.V., Leiden, The Netherlands; ^2^Hungarian Angioedema Center of Reference and Excellence, Department of Internal Medicine and Haematology, Semmelweis University, Budapest, Hungary; ^3^Attoquant Diagnostics, Vienna, Austria; ^4^Sexton Bio Consulting, LLC, Melrose, MA, USA; ^5^Globalization Partners, Munich, Germany; ^6^GrayMatters Consulting, Schilde, Belgium

##### **Correspondence:** Evangelia Pardali (lia.pardali@pharvaris.com)

***Allergy, Asthma & Clinical Immunology*** 2025, **21(Suppl 2)**:O-02

**Background:** Angioedema (AE) is classified into three endotypes: bradykinin (BK)-mediated (AE-BK), mast cell-mediated and AE due to intrinsic vascular endothelium dysfunction. Differentiating AE-BK from other types of AE and assessing the role of BK in the pathogenesis of other conditions remains a challenge. Qualification and clinical validation of a bioanalytical method to accurately measure BK and related peptides could aid in identifying and managing BK-mediated pathologies.

**Methods:** An ultra-high performance liquid chromatography-tandem mass spectrometry (UHPLC-MS/MS) protocol was developed and qualified to measure kinin peptides (BK1-9, BK1-8, BK1-7, BK1-5) in plasma. Twenty-seven people with hereditary angioedema (HAE) due to C1-inhibitor deficiency (HAE-C1-INH), 4 people with HAE with normal C1-INH function (HAE-nC1-INH; with variants in F12 or PLG genes), and 8 healthy volunteers (HVs) provided informed consent. People with HAE did not receive any prophylactic therapy. They did not use on-demand therapy during the 4 days before sampling. Kinins were analysed in plasma collected from 14 HAE-C1-INH and 8 HVs (prospective samples) and in plasma obtained from the Biorepository of the Hungarian Angioedema Center of Reference and Excellence from 16 HAE-C1-INH (3 were also included in prospective part of the sudy) and 4 HAE-nC1-INH participants (biobank samples). Kinin levels were analysed in EDTA plasma before and after kallikrein-kinin system (KKS) activation by exposure to cold temperature (4 °C) for 24 h to assess KKS pathway sensitivity.

**Results:** Baseline kinin levels before cold activation were significantly (p < 0.001) higher in prospective as well as biobank plasma samples from people with HAE-C1-INH compared to HVs: baseline mean BK1-9 levels were 7 ng/mL, 79.7 ng/mL, and 1.1 ng/mL, respectively. Cold activation resulted in significant (p < 0.001) increase of kinin peptides in prospective and biobank plasma samples from HAE-C1-INH compared to HVs: mean BK1-9 levels following cold activation were 160.6 ng/mL, 591.3 ng/mL, and 6.9 ng/mL, respectively. Cold activation also resulted in significantly increased BK1-9 levels in plasma from people with HAE-nC1-INH (mean baseline 10.7 ng/mL, mean cold activated 483 ng/mL).

**Conclusions:** The qualified UHPLC-MS/MS assay can be used to reliably measure kinin levels and to characterise people with AE-BK. Clinical validation of the biomarker assay suggests that it can be used to assess the hyper-activatable state of the KKS in subjects with HAE-C1-INH and HAE-nC1-INH. Importantly, our results indicate that the assay also allows to measure KKS pathway sensitivity in plasma samples from biorepositories for patients with different AE endotypes. The clinically validated BK assay may become a key tool for identifying, studying, and managing BK-mediated conditions, including AE-BK.

**Conflicts of Interest Disclosures**:

E. Pardali: employee of Pharvaris, holds stocks/stock options in Pharvaris

HR. Horváth: received travel grants from Takeda and CSL Behring

O. Domenig: CEO of Attoquant Diagnostics GmbH.

D. van Oyen: employee of Attoquant Diagnostics GmbH

D. Sexton: Principal at Sexton Bio Consulting, LLC

G. Zahn: employee of Globalization Partners GmbH, consultant to Pharvaris, holds RSU

A. Lesage: employee of Gray Matters Consulting and consultant to Pharvaris, holds stocks/stock options in Pharvaris; advisor to Kosa Pharma.

H. Farkas: received research grants from CSL Behring, Pharming, Takeda and served as an advisor for these companies and BioCryst, Intellia, KalVista, ONO Pharmaceutical, Pharvaris; has participated in clinical trials/registries for BioCryst, CSL Behring, KalVista, Pharming, Pharvaris, Takeda.

## O-03 Impact of C1-inhibitor binding to endothelial cells on the regulation of vascular permeability

### Debora Parolin^1^, Silvia Berra^1,2^, Sonia Caccia^1^

#### ^1^Department of Biomedical and Clinical Sciences, Università degli Studi di Milano, Milan, Italy; ^2^Department of Internal Medicine, ASST Fatebenefratelli Sacco, Milan, Italy

***Allergy, Asthma & Clinical Immunology*** 2025, **21(Suppl 2)**:O-03

**Background:** C1-inhibitor deficiency results in recurrent episodes of increased vascular permeability that are transient in nature and usually self-limiting.

These swellings are a consequence of abnormal activation of the contact system (CAS), of which C1-inhibitor is the main regulator. CAS activation leads to activated plasma kallikrein (PKa) with cleavage of high molecular weight kininogen (HK) and eventual release of the nonapeptide bradykinin (BK). BK interacts with the B2 receptor on endothelial cells (ECs), which mediates increased vascular permeability and leakage of plasma into the interstitial space.

As ECs play a major role in the pathogenesis of angioedema, it is important to understand their role in this scenario.

The binding of C1-inhibitor to ECs has been suspected but never studied in detail. The influence of this binding on the regulatory activity of C1-inhibitor on CAS remains controversial.

**Methods:** To study C1-inhibitor binding to ECs and its functional consequences, we used confocal immunofluorescence microscopy and set up a functional assay based on a modified version of the XPerT technique, which allows permeability measurement by exploiting the binding of fluorescent avidin to biotinylated gelatin used as a growth support for ECs.

**Results:** Our results show that C1-inhibitor binds to the EC monolayer. The C1-inhibitor signal is mainly localised at inter-endothelial junctions and diRuses to the basement membrane.

In the functional assay, we show that incubation of PK and HK on EC monolayers induces permeability, whereas pre-treatment with C1-inhibitor protects ECs and prevents PK-induced permeability. A similar but less pronounced eRect is observed on PKa.

**Conclusion:** Our results indicate that cell-bound C1-inhibitor can inhibit PK activation and attenuate PKa-induced EC permeability.

## O-04 Systemic inflammation biomarkers in different endotypes of angioedema

### Johana Gil-Serrano MD^1,2,3^, Moisés Labrador-Horrillo MD, PhD^1,2,3^, Paula Galván-Blasco MD^1,2^, Anna Sala-Cunill MD, PhD^1,2,3^, Javier Pereira-González MD^1,2^, Marta Planas-Vinos MD^1,2^, Victoria Cardona MD, PhD^1,2^, Mar Guilarte MD, PhD^1,2,3^

#### ^1^Department of Allergy, Hospital Universitari Vall d’Hebron, Barcelona, Spain; ^2^Allergy Research Unit, Institut de Recerca Vall d’Hebron (VHIR), Barcelona, Spain; ^3^Department of Medicine, Universitat Autònoma de Barcelona, Spain

***Allergy, Asthma & Clinical Immunology*** 2025, **21(Suppl 2)**:O-04

**Background:** AE can be classified depending on its endotype and its aetiology in mast cell AE (AE-MC), Bradykinin AE (AE-BK), drug-induced AE (AE-DI) and the unknown aetiology AE (AE-UNK). Despite the underlying mechanism in AE the role of inflammation has been poorly explored. We aim to analyse the role of inflammatory mediators in AE patients during AE attacks.

**Methods:** We enrolled patients with different types of AE including: **AE-BK** (confirmed hereditary angioedema (HAE) due to C1-inhibitor deficiency, patients with F12 gene mutations and acquired C1-INH deficiency AE, **AE-MC**, **AE-DI** (due to angiotensin converting enzyme-ACEi and to rTPA) and Chronic Spontaneous Urticaria with AE **(CSU-AE)** as control group. The study was conducted between November-2019 and December 2024.

We analysed demographic and clinical characteristics of all AE patient groups. Blood samples were collected both during symptom-free periods (baseline) and during AE attacks and acute phase reactants (APR), such as serum amyloid A (SAA), erythrocyte sedimentation rate (ESR), C-reactive protein (CRP), D-Dimer and white blood cells, among others were measured.

**Results:** A total of 380 patients were included, of whom 56% (n = 214) were women, with a mean age of 57.6 years (range: 2–96). Regarding AE endotypes, 47% (n = 178) had AE-MC, 30% (n = 115) had AE-BK, 17% (n = 66) had AE-ACEi, 0.5% (n = 2) had AE-rTPA, and 5% (n = 19) had CSU-AE. During attack-free periods, most patients exhibited normal levels of APR. However, during AE attacks, significant increases were observed in SAA (77% of patients, p < 0.0001 vs. baseline), ESR (54%, p < 0.001 vs. baseline), and D-dimer (74%, p < 0.001 vs. baseline). In contrast, CRP was elevated in only 25% of patients, while 22% showed an increase in WBC. These findings remained consistent across all AE types, with no statistically significant differences compared to the control group.

**Conclusion:** Despite there are distinct AE endotypes, the observed increase in APR across all patients suggests an underlying inflammatory response that extends beyond the known pathogenic mechanisms. Further research is needed to elucidate the role of inflammation in AE attacks and its potential clinical implications.

**Keywords** Angioedema, Mast cell, Bradykinin, Inflammation, Acute phase proteins, D-dimer, Serum amyloid A, Erythrocyte sedimentation rate

## O-05 Identification of candidate biomarkers for diagnosing and assessing the disease severity of type 2 hereditary angioedema via plasma N-glycomics

### Yingyang Xu^1^, Xiangyi Cui^1^, Zejian Zhang^2,*^, Yuxiang Zhi^1,*^

#### ^1^Department of Allergy & Clinical Immunology, National Clinical Research Center for Immunologic Diseases, Peking Union Medical College Hospital, Chinese Academy of Medical Sciences and Peking Union Medical College, Beijing, China; ^2^State Key Laboratory of Complex Severe and Rare Diseases, Peking Union Medical College Hospital, Chinese Academy of Medical Sciences and Peking Union Medical College, Beijing, China

##### **Correspondence:** Yuxiang Zhi (Yuxiang_zhi@126.com); Zejian Zhang (1150678666@qq.com) equally to this work as co-corresponding authors

***Allergy, Asthma & Clinical Immunology*** 2025, **21(Suppl 2)**:O-05

**Rationale:** Hereditary angioedema (HAE) is a rare, life-threatening genetic disease characterized by acute, recurrent episodes of cutaneous and/or submucosal edema. There is great heterogeneity in disease severity. Biomarkers for disease stratification are currently unavailable. Plasma-derived N-glycans have demonstrated remarkably clinical value in the risk stratification, diagnosis, and prognosis of type 1 HAE and other common diseases. This study aims to explore potential plasma N-glycan biomarkers for the diagnosis and stratification of HAE-2.

**Methods:** We analyzed the total plasma N-glycomes of 12 patients with HAE-2, 28 patients with mast cell-mediated angioedema (MC-AE), and 14 healthy controls (HC) by utilizing high-throughput matrix-assisted laser desorption/ionization time-of-flight mass spectrometry with linkage-specific sialyation derivatization.

**Results:** Compared with the patients with MC-AE and HCs, the patients with HAE-2 exhibit 10 abnormal N-glycans. According to a classification model of dysregulated N-glycan traits, the area under the curve (AUC) ranges from 0.905 to 1.000, effectively distinguishing the patients with HAE-2 from those with MC-AE and HCs. Furthermore, we identified a series of promising markers closely associated with the occurrence of laryngeal or abdominal edema and can effectively differentiate between HAE-2 with and without laryngeal or abdominal edema. The classification model demonstrated robust performance, with AUCs ranging from 0.971 to 1.000 for laryngeal edema and 0.829 to 0.886 for abdominal edema. By utilizing two commonly used severity assessment scales for HAE, we found that 2 specific N-glycans were strongly correlated with disease severity (r > 0.8, p < 0.01), enabling the differentiation of patients with varying disease severities.

**Conclusions:** For the first time, this study presented the total plasma N-glycomic profile of HAE-2 and identified a series of novel glycan biomarkers with significant potential for HAE-2 diagnosis, edema attack site prediction, and severity assessment. The findings hold substantial clinical importance and provide valuable insights to advance the clinical diagnosis and management of HAE-2.

**Acknowledgements** We thank all members of our group involved in this study. We thank the patients and volunteers who provided blood samples for the present study. The study was supported by the CAMS Innovation Fund for Medical Sciences (CIFMS) (2022-I2M-C&T-B-004), the National High Level Hospital Clinical Research Funding (2022-PUMCH-B-090), the National Natural Science Foundation of China (82271815), the Beijing Natural Science Foundation (L222082), and the Special Research Fund for Central Universities, Peking Union Medical College (3332024108).

## O-06 Inhibition of bradykinin liberation by plasma kallikrein inhibitors ameliorates vaso-genic edema in experimental (Murine) cerebral malaria independent of and in addition to artesunate

### Alessandro S. Pinheiro^1^, Douglas E. Teixiera^1^, Rodrigo P. Silva-Aguiar^1^, Alona A. Merkulova^1^, Yelenna Skomorovska-Prokvolit^2^, Young Jun Shim^3^, Keith R. McCrae^3^, David Midem^4^, Sidney Ogolla^5^, Celso Caruso Neves^6^, Ana Acacia S. Pinheiro^6^, James W. Kazura^2^, Alvin H. Schmaier^1,7^

#### ^1^Department of Medicine, School of Medicine, Case Western Reserve University, Cleveland, OH, USA; ^2^Department of Pathology, Center for Global Health & Diseases, School of Medicine, Case Western Reserve University, Cleveland, OH, USA; ^3^Department of Hematology and Medical Oncology, Cleveland Clinic Foundation, Cleveland, OH, USA; ^4^Chulaimbo Sub-County Hospital, Kenya; ^5^Kenya Medical Research Institute, Kisumu, Kenya; ^6^Carlos Chagas Filho Biophysics Institute, Federal University of Rio de Janeiro, Brazil; ^7^University Hospitals Cleveland Medical Center, Cleveland, OH, USA

***Allergy, Asthma & Clinical Immunology*** 2025, **21(Suppl 2)**:O-06

**Background:** Cerebral malaria (CM) due to *Plasmodium falciparum* infection kills > 500,000 children in Africa annually. It manifests with coma and brain swelling due to vasogenic edema. *Hypothesis: BK is a proximal cause of vasogenic edema in cerebral malaria.* We studied this disease in *Plasmodium bergehi-ANKA-* (*PbA*-) infected mice and human plasma samples.

**Results:** *PbA*-infected mice had significantly reduced plasma HK and PK activity and antigen levels versus uninfected mice. *PbA*-infected plasma had circulating cleaved cHK with 56 and 46 kDa bands. Murine bradykinin (BK) levels measured by LCMSMS assay were increased 3–fourfold over normals. Plasma FXII levels were unchanged between groups.

*PbA*-infected *Kng1*^*−/−*^ mice were protected significantly from neurologic deterioration vs WT mice (P = 0.0063) and had a 58% reduction in brain edema (P = 0.0006*).* Likewise, *PbA*-infected combined *Bdkrb1*^*−/−*^*/Bdkrb2*^*−/−*^ or *Bdkrb2*^*−/−*^ mice also had significant protection from neurologic deterioration and brain edema. In contrast, *PbA*-infected *F12*^*−/−*^ mice only had a 33% reduction in brain edema (P = 0.019) but no protection from neurologic deterioration. Both *PbA*-infected *Klkb1*^*−/−*^ and *Prcp*^gt/gt^ mice had significant protection protected from neurologic deterioration and brain edema. Prolylcarboxypeptidase is a PK activator in vessel wall independent of FXIIa. *PbA*-infected wild type mice treated with a PrCP inhibitor also had a significant reduction in neurologic deterioration and brain edema. Likewise, *PbA*-infected wild type mice treated with a plasma kallikrein inhibitor (RZLT7824) had significantly reduced neurologic deterioration and brain edema. When *PbA*-infected mice on day 5 were given the anti-parasite therapy artesunate vs artesunate and RZLT7824 treatment, mice given the combined therapy had significantly improved neurologic behavior, reduced brain edema and mass, and survival (33% better) at 24 h.

Forty percent of human plasmas from CM patients on presentation when examined on immunoblot showed cHK with 56 and 46 kDa bands vs 18% uncomplicated malaria patients. HK levels of CM patients at hospital admission also were significantly lower and compared to healthy children and children with non-malaria illness.

**Conclusion:** In murine and human CM circulating HK is commonly cleaved (cHK). In murine CM, kininogen, prekallikrein, prolylcarboxypeptidase, or bradykinin receptor deficiency ameliorates the disorder. Factor XII deficiency does not. Pasma kallikrein and prolylcarboxypeptidase inhibitors block cerebral edema and neurologic deterioration of mice. The plasma kallikrein inhibitor RZLT7824 when given with artesunate reduces brain edema and increases neurologic recovery independent of and in addition to anti-parasite therapy.

## O-07 Differentiating subtypes of angioedema with machine learning

### Toan Do MD^1,*^, Eileen Kim MD^2^, Bruce Zuraw MD^1^, Marc Riedl MD^1^

#### ^1^Division of Allergy & Immunology, University of California San Diego, La Jolla, California, USA; ^2^Division of Internal Medicine, University of California San Diego, La Jolla, California, USA

##### **Correspondence:** Toan Do (ttd001@health.ucsd.edu)

***Allergy, Asthma & Clinical Immunology*** 2025, **21(Suppl 2)**:O-07

**Background:** The rapid and accurate diagnosis of subtypes of angioedema can be clinically challenging due to overlapping symptoms and lack of quick-resulting diagnostics. Hereditary angioedema (HAE types I and II) is an autosomal dominant disorder causing bradykinin-mediated angioedema and is associated with significant diagnostic delays. Machine learning (ML), a form of artificial intelligence, may be an effective clinical tool to differentiate between bradykinin- and histamine-mediated angioedema.

**Methods:** A retrospective chart review of n = 204 subjects with angioedema was performed at the United State Hereditary Angioedema Association Center. Structured data of clinical characteristics and diagnostic laboratory data was analyzed with various machine learning classification algorithms (logistic regression, naive Bayes, etc.) with random forest ultimately being chosen. Five test records were removed from analysis for final testing (3 histamine-mediated and 2 bradykinin-mediated angioedema).

Bootstrapping with 80–20% train-validation instances was done iteratively. Features were iteratively eliminated according to importance in models with higher predictive accuracy. Decision trees were generated to elucidate the relationships between features and diagnosis.

**Results:** The cohort of (n = 204) were mostly female (58%) and white (62%), with 48% (n = 98) with HAE Type I, II, or ACE-inhibitor-induced angioedema. Among those with bradykinin-mediated angioedema, 55% had a strong family history, with average age of onset of symptoms at 21 years old, and average duration of a single angioedema episode of 77 h. Random forest models, with 30 iterations of cross-validation, that included seven clinical features of: age of onset, pruritus, atopy history, family history, and abdominal symptoms differentiated the subtypes of angioedema with an average accuracy of 93% ± 4. Random forest models which included chromogenic functional level of C1 esterase inhibitor function along with four clinical features of attack duration, age of onset, pruritus, family history resulted in an average accuracy prediction rate of 96% ± 3.

**Conclusions:** Machine learning is a powerful tool to differentiate between bradykinin and mast cell-mediated angioedema with high average accuracies of ≥ 90%. The addition of diagnostic laboratory data (C4 and C1 esterase function) did not significantly improve diagnostic accuracy in our predictive models; however, remains a critical component of confirming subtypes of angioedema. This ML-based approach has potential clinical application in the diagnosis and management of hereditary angioedema, and may have utility in scenarios where laboratory testing is not readily available or the management strategy remains uncertain (Fig. 1).

**Figure 1 (abstract O-07):**
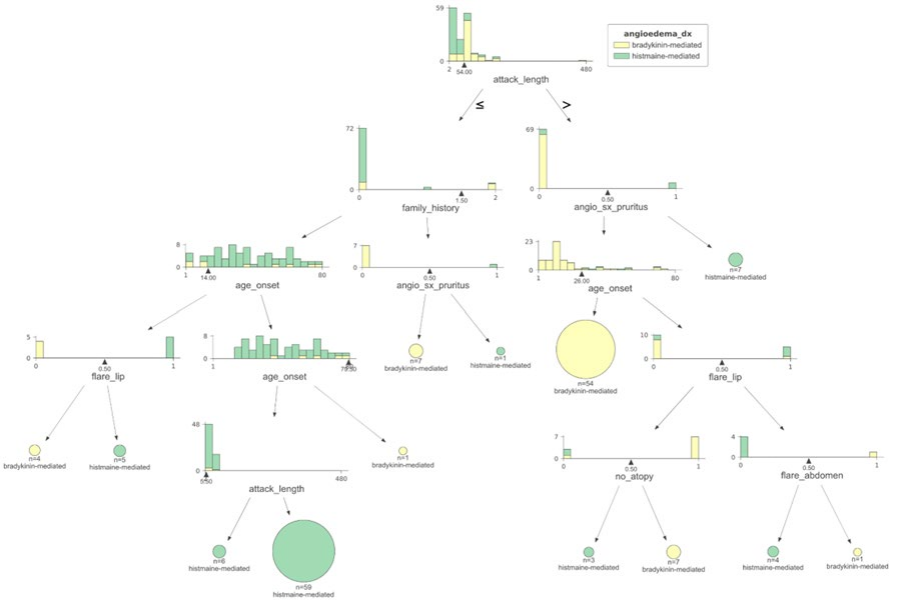
Decision tree with 7 clinical features

## O-08 Clinical and genetic characterization of three families with homozygous SERPING1 mutations in hereditary angioedema

### Kutay Kırdok^1^, Asuman Camyar^2,*^, Melih Ozisik^3^, Huseyin Onay^4^, Ayca Aykut^4^, Nihal Mete Gokmen.^1^

#### ^1^Ege University, Department of Internal Medicine, Division of Immunology and Allergy, Izmir, Turkey; ^2^Bakırcay Training and Research Hospital, Izmir, Turkey; ^3^Izmir City Hospital, Izmir, Turkey; ^4^Ege University Faculty of Medicine, Department of Medical Genetics, Izmir, Turkey

##### **Correspondence:** Kutay Kırdok (kirdokkutay@gmail.com)

***Allergy, Asthma & Clinical Immunology*** 2025, **21(Suppl 2)**:O-08

**Background:** Hereditary angioedema due to C1-inhibitor deficiency (HAE-C1-INH; OMIM#106100) is an uncommon autosomal dominant disorder, characterized by recurrent episodes of angioedema affecting the skin and mucous membranes without accompanying urticaria. The condition typically arises from heterozygous mutations in the SERPING1 gene, which encodes the C1-INH protein. This report presents three families exhibiting homozygous mutations in the SERPING1 gene, including two previously documented variants (I379T and S438F) and one newly identified mutation (C1198c). These cases demonstrate autosomal recessive inheritance.

**Materials and Methods:** Eighteen patients from three families with homozygous SERPING1 mutations were diagnosed with HAE-C1-INH and followed at the Department of Immunology and Allergy, Ege University. The families were anonymized as A (n = 7), D (n = 5), and DY (n = 6). Data collected included age, symptomatic status, age at onset, attack location, and annual attack frequency. Serum levels of C4, C1-INH, C1-INH function and C1q were measured. Genetic analysis was conducted to identify mutations.

**Results:** Homozygous individuals comprised 2 from family A, 2 from family D, and 3 from family DY. The mean age of onset in homozygotes was 11.7 years (range: 5–15), with a mean annual attack rate of 29.4 (range: 20–35) and a mean C1-INH function of 10.4% (range: 2–18). In contrast, heterozygous individuals demonstrated later onset (mean: 29.8 years), milder C1-INH deficiency (52.9%), and a comparable attack rate (25.9/year). Family A (I379T) exhibited mild symptoms and normal C4/C1q levels. Family D (S438F) presented more severe symptoms and autoimmune comorbidities associated with low C1q levels. The newly identified Family DY (C1198c) had a later onset with predominantly gastrointestinal and laryngeal involvement. (Table 1).

**Table 1 (abstract O-08):** Clinical and Laboratory Features of Hereditary Angioedema Patients (P: Peripheral, F: Face, GIS: Gastrointestinal, G: Genital, L: Laryngeal)

Patient number	F/M	Age	SERPING1 mutation	Age of unset	Localisation of attack	Annual attack frequency	C1-INH (21–39 mg/dL)	C1-INH function (70–130%)	C4 (10–40 mg/dL)	C1q (100–300 µg/mL)	HAE Type
A10	F	48	I379T hom	27	P, GIS, G	2	10.0	18.3	10	248.0	I
A20	F	45	I379T hom	24	P	< 1	12.8	29.6	21	160.0	I
A30	F	23	I379T het	–	–	–	21.3	60.9	22	137.0	II
A50	F	20	I379T het	–	–	–	21.2	62.1	27	150.0	II
A70	M	72	I379T het	70	P	< 1	30.9	97.5	34	138.0	Normal
A80	F	74	I379T het	–	–	–	24.4	71.1	32	141.0	Normal
A170	F	14	I379T het	–	–	–	15.6	54.9	14	151.0	I
D10	F	55	S438F hom	5	P, F, GIS, G	24	2.0	2.0	6	5.0	I
D20	F	67	S438F hom	7	P, F, GIS, G, L	> 60	3.0	0.0	6	1.66	I
D40	M	61	S438F het	21	P, F, GIS, G, L	3	12.0	15.0	7	232.0	I
D50	F	57	S438F het	–	–	–	12.2	42.7	11	177.0	I
D70	F	27	S438F het	–	–	–	11.0	36.0	8	321.0	I
DY39	F	85	C1198c het	–	–	–	15.4	46.3	15	126	I
DY64	F	61	C1198c hom	7	P, F, GIS, G, L	96	2.9	0.2	6	11	I
DY59	F	66	C1198c hom	6	P, F, GIS	12	2.6	10.5	6	0	I
DY67	F	58	C1198c hom	6	GIS, F	12	3.2	11.7	6	9	I
DY94	F	30	C1198c het	21	P	< 1	14.3	48.0	13	115	I
DY98	F	26	C1198c het	11	GIS	> 60	14.8	56.5	13	110	I

**Conclusions:** Homozygous mutations in the SERPING1 gene, although uncommon, may lead to autosomal recessive inheritance of HAE-C1-INH. Within our study group, individuals with homozygous mutations exhibited significantly earlier onset of symptoms (p = 0.029) and reduced C1-INH functionality (p = 0.006) compared to heterozygous subjects. However, there was no significant difference in annual attack frequency between the two groups (p = 0.342). These results underscore the necessity for SERPING1 genotyping and biochemical assessment in patients presenting with early-onset or severe manifestations of HAE-C1-INH.

## O-09 What can we learn from genome sequencing in HAE C1-INH-nl?

### Julie Gauthier^1,2,4,*^, Francois Marceau^3^, Blandine Monjarret^2^, Arnaud Bonnefoy^2,4^, Fadi F. Hamdan^1,2^, Nina Verreault^5^, Fabian Touzot^2,6^, Quang-Hien Le^7^, Clémence Merlen^2^, Georges E Rivard^1,2,4^

#### ^1^Molecular Diagnostic Laboratory of CHU Sainte-Justine; Department of Pediatrics, Faculty of Medicine, Université de Montréal, Montréal, QC, Canada; ^2^CHU Sainte-Justine Research Center, Montréal, QC, Canada; ^3^Axe Microbiologie-Infectiologie et Immunologie, CHU de Québec, Université Laval, QC, Canada; ^4^Centre d'hémostase pédiatrique et adulte du CHU Sainte-Justine, Université de Montréal, Montréal, QC, Canada; ^5^Division of Allergy and Clinical Immunology, Department of Medicine, CHU de Québec, Université Laval, QC, Canada; ^6^Department of Microbiology, Infectiology and Immunology, Université de Montréal; Department of Pediatrics, Faculty of Medicine, Université de Montréal, Montréal, QC, Canada; ^7^Centre Québécois de Génomique Clinique, CHU Sainte-Justine, Montréal, QC, Canada

##### **Correspondence:** Julie Gauthier (julie.m.gauthier.hsj@ssss.gouv.qc.ca)

***Allergy, Asthma & Clinical Immunology*** 2025, **21(Suppl 2)**:O-09

To date, very few genes have been linked to HAE C1-INH-nl, and thus molecular diagnostic options are very limited to individuals affected by this condition. The absence of molecular testing could lead to individuals receiving incorrect diagnoses and inadequate treatments. To address this, we launched a project that combines family genome sequencing and functional testing to obtain a “biological signature” for diagnostics. These tests could help identify effective, targeted, and personalized treatments based on pathophysiology.

We recruited individuals affected by HAE C1-INH-nl and their symptomatic and asymptomatic family members across the Province of Quebec (Canada) to identify the genetic cause and confirm the pathophysiology through functional assays. In total 93 individuals have been recruited so far from 20 families. Illumina short read genome sequencing (50x) has been completed in over 60 individuals. One of the sequenced families included a female patient who has been investigated for several years in search of pathophysiological explanation for her HAE C1-INH-nl with typical clinical manifestations. She has good clinical response to treatment with HAE prophylaxis that significantly diminished her HAE attacks. This patient was found to harbor a pathogenic heterozygous nonsense mutation in *NLRP12*, a gene involved in regulating inflammatory responses through neutrophil expression^1^. Interestingly, dominant mutations in *NLRP12* are known to cause Familial cold autoinflammatory syndrome 2 (MIM 611762). We hypothesized that CD87 (uPAR) levels could be dysregulated in our patient with the *NLRP12* mutation, and that HAE C1-INH-nl may be either a differential diagnosis of cold-induced autoinflammatory syndrome type 2 or part of the clinical spectrum associated with *NLRP12* mutations. Significant neutrophil dysregulation, especially CD87 increased expression, has been observed in HAE C1-INH^2^. We are currently testing CD87 levels in phagocytes in this HAE C1-INH-nl patient and her family members as well other affected individuals in our cohort.

Our ongoing genome project supports the clinical utility of genome sequencing of individuals with HAE C1-INH-nl to identify the genetic abnormality and shorten the well-known clinical diagnostic odyssey for this condition. In addition, our results might increase the understanding of the interacting pathophysiology between the bradykinin and immune systems.

**References**

^1^PMID: 39076995; ^2^ PMID: 31236065

## O-10 Preliminary demographic and clinical data from a cohort of Spanish HAE-C1-INH patients: The GenomAEH Study

### Mar Guilarte^1^, Alberto López-Lera^2^, Ethel Ibáñez-Echevarría^3^, Krasimira Baynova^4^, Carmen Marcos-Bravo^5^, Eugenia Sanchis-Merino^6^, Gabriela Leon-Zambrana^7^, Patricia Bigas Peñuela^1^, Leah Landaveri-Sánchez^3^, Stefan Cimbollek^4^, Johana Gil-Serrano^1^, Marta Goyanes-Malumbres^8^, Isora Vidal-Sernandez^9^, Roger Colobran^10,11^, Teresa Caballero^12^

#### ^1^Allergy Department, Hospital Universitari Vall d'Hebron (HUVH), Allergy Research Unit, Vall d'Hebron Research Institute (VHIR), Barcelona, Spain; ^2^Hospital La Paz Institute for Health Research (IdiPAZ), CIBERER (U754), Madrid, Spain; ^3^Allergy Department, Hospital Universitari i Politècnic La Fe (HUPLAFE), Valencia, Spain; ^4^Angioedema Reference Center, Allergy Department, Hospital Universitario Virgen del Rocío, Sevilla, Spain; ^5^Allergy Department, Complejo Hospitalario Universitario de Vigo, Spain; ^6^Allergy Unit, Hospital Universitario Rio Hortega, Valladolid, Spain; ^7^Allergy Department, Hospital Universitario La Paz, Madrid, Spain; ^8^Allergy Department, Hospital Universitario La Paz, Hospital La Paz Institute for Health Research (IdiPAZ), Madrid, Spain; ^9^Takeda Farmacéutica, Madrid, Spain; ^10^Immunology Division, Genetics Department, Translational Immunology Research Group, Hospital Universitari Vall d'Hebron (HUVH), Vall d'Hebron Research Institute (VHIR), Barcelona, Spain; ^11^Department of Cell Biology, Physiology and Immunology, Universitat Autònoma de Barcelona (UAB), Bellaterra, Spain; ^12^Allergy Department, Hospital Universitario La Paz, Hospital La Paz Institute for Health Research (IdiPAZ), CIBERER (U754), Madrid, Spain

***Allergy, Asthma & Clinical Immunology*** 2025, **21(Suppl 2)**:O-10

Hereditary angioedema due to C1-INH deficiency (HAE-C1-INH; MIM #106100) is a complex disorder characterised by a significant clinical variability, despite its monogenic nature. This variability persists even among individuals who share identical genetic mutations, hindering diagnosis and suggesting the presence of yet unknown genetic factors acting as disease modifiers.

The GenomAEH is a multicentric, cross-sectional, observational study carried out in Spain. The ultimate goal is to analyse, using an unbiased approach, the existence of genetic variants contributing to differences in clinical expression on a large cohort of Spanish HAE-C1-INH patients, categorized by disease severity and activity scores. At this stage, we present the analysis of the clinical and demographic data from the study cohort. The study has been approved by an Independent Ethics Committee.

Recruitment was performed between 2023 and 2024, including biological samples and medical history data from 189 patients (from 93 families) across six Spanish centres. The median age was 44 years (range 18–88), with 60.3% of participants being women. The mean [SD] Body Mass Index (BMI) was 26 [6], and comorbidities were present in 74.6% of patients. A majority, 181 patients (95.8%), were diagnosed with HAE-C1-INH-Type1, and the median age at the onset of symptoms was 12 years (range 0.8–46). Peripheral attacks were the most common, affecting 92.2% of patients, followed by abdominal attacks (85.7%), facial attacks (46.0%), genital attacks (37.0%), and laryngeal attacks (32.3%). Notably, 6 patients (3.2%) were asymptomatic. Concerning attack frequency in the previous year, 38.6% experienced 1 to 5 attacks, 16.4% had 6 to 12 attacks, 9.5% had 13–24 attacks, and 4.8% had 24–52 attacks. Notably, 25.9% of patients were attack-free in the last year, with 55.1% of these receiving long term prophylaxis (LTP). Prodromal symptoms were reported by 56.1% of patients prior to attacks, being most frequent erythema marginatum (38.7% of patients), and 84.7% could identify an attack trigger: 66.9% cited stress, 56.2% trauma, 36.2% infections, 27.5% menstruation, 15.6% pregnancy, 9.4% exogenous oestrogens, and 22.5% other factors. At recruitment, 50.8% of patients were receiving LTP. Mutations in the SERPING1 gene have been evaluated by sequencing.

This descriptive analysis of the GenomAEH cohort provides valuable insights into the clinical and demographic profiles of Spanish HAE-C1-INH patients. Ultimately, we expect the findings from GenomAEH to enhance clinical management, improve patient outcomes, and contribute to the broader understanding of HAE.

Takeda Farmacéutica España SA provided funding for conducting the study.

**Conflicts of interest declared by the authors**:

Mar Guilarte has participated as a principal investigator in clinical trials sponsored by CSL Behring, Takeda, Pharvaris, Jerini AG/Shire, Pharming NV, BioCryst, Biomarin, Ionis, and Kalvista; and offered consultancy and educational services for CSL Behring, Shire/Takeda, BioCryst, Kalvista, and Pharvaris.

Alberto Lopez-Lera declares the following, real or perceived conflicts of interest: receiving grants/research support: Takeda Pharmaceutical Company; Receiving honoraria or consultation fees: Shire Pharmaceuticals Ibérica /Takeda Pharmaceutical Company; Participating in sponsored speaker bureau: Shire Pharmaceuticals Ibérica /Takeda Pharmaceutical Company, CSL Behring.

Ethel Ibañez has received fees as a speaker and consultant from Takeda, CSL Behring, and Biocryst. Additionally, she has received funding from Takeda and CSL Behring to attend events and congresses and participated in clinical studies of CSL Behring, Ionis, and Takeda.

Krasimira Baynova has received fees for speaking engagements and for participating in the medical board of the following companies: CSL Behring, Takeda, Biocryst.

Carmen Marcos-Bravo has received educational sponsorships, speaker fees, participated in advisory boards, and travel grants from Takeda, CSL Behring, and Biocryst.

Eugenia Sanchis-Merino has participated in communications sponsored by Takeda, CSL Behring, Novartis, and in Clinical Trials promoted by Roche, Bayer, Iveric, Roche, Bayer, Iveric.

Gabriela Leon-Zambrana has received funding to attend conferences and educational events from CSL Behring and Takeda.

Patricia Bigas-Peñuela declares no conflicts of interest.

Leah Landaveri-Sanchez declares no conflicts of interest.

Stefan Cimbollek has received educational sponsorships, speaker fees, and participated in advisory boards and travel grants from Novartis, Pharming, Takeda, CSL Behring, and Biocryst, and participated in clinical trials sponsored by Novartis, Jerini/Shire, CSL Behring, Pharming, and Ionis.

Johana Gil-Serrano declares no conflicts of interest.

Marta Goyanes-Malumbres declares no conflicts of interest.

Isora Vidal-Sernandez is an employee of Takeda Farmaceutica España SA

Roger Colobran has received honoraria for lectures / courses / seminars from Takeda, CSL Behring, Biocryst, Sanofi and Health in Code.

Teresa Caballero has declared the following conflicts of interest: Organization: Hospital Universitario La Paz; Research Organizations: IdiPAZ (researcher in the IdiPAZ program to promote research activities), CIBERER (Centro de Investigación en Red de Enfermedades Raras); Scientific Societies: SEAIC (Angioedema Committee), SMCLMAIC, EAACI, AAAAI; Medical Advisory Board: AEDAF, HAEi; Speaker Fees: BioCryst, CSL-Behring, Novartis, Pharming Group NV, Takeda; Consultancy Fees: Astria, BioCryst, CSL-Behring, KalVista, Novartis, Pharming NV, Pharvaris, Takeda; Funding for Congress Attendance: BioCryst, CSL-Behring, Novartis, Pharming NV, Takeda; Clinical Trials and Registries Investigator: BioCryst, Biomarin, CSL-Behring, KalVista, IONIS, Novartis, Pharming Group NV, Takeda Pharmaceuticals; Participation in Collaborative Projects: Takeda; Editorial Support for Manuscript Publication: BioCryst, CSL-Behring; Research Grants: AEDAF, CSL-Behring, Takeda; Writing and Editorial Support: BioCryst, CSL-Behring, KalVista, Pharvaris.

## O-47 The reduction of hereditary angioedema attacks following significant weight loss achieved through bariatric surgery

### Marko Barešić^1^, Boris Karanović^1^, Domagoj Vergles^2^, Branimir Anić^1^

#### ^1^Division of Clinical Immunology and Rheumatology, Department of Internal Medicine, School of Medicine, University Hospital Center Zagreb, Zagreb, Croatia; ^2^Division of Endoscopic and Bariatric Surgery, Department of Surgery, Clinical Hospital Dubrava, Zagreb, Croatia

***Allergy, Asthma & Clinical Immunology*** 2025, **21(Suppl 2)**:O-47

The hallmark of hereditary angioedema (HAE) is its unpredictability, with attacks arising spontaneously or in response to triggers. Common triggers include infections, illnesses, hormonal changes (menstrual cycle, pregnancy, estrogen-containing medications), environmental factors, medications (ACE inhibitors and angiotensin receptor blockers), emotional stress, physical trauma, and medical procedures.

Diagnosed with HAE type I at age 16, our patient initially presented with extremity and facial swelling. She had no family history of similar manifestations. Recurrent attacks were managed with on-demand treatment (ODT) and long-term prophylaxis (LTP) available at the time (tranexamic acid and danazol). The patient experienced the emotional overeating as a maladaptive coping mechanism in response to objective psychosocial stressors, including familial and occupational difficulties. Over time, this led to significant weight gain prompted by the use of high doses of danazol. Due to high number of attacks she frequently required ODT with icatibant and C1 esterase inhibitor. By the age of 33, she weighed 166 kg, with a BMI of 58.81 kg/m2, classifying her as extremely obese. In 2022, first-line LTP treatments (Lanadelumab and berotralstat) and GLP-1 receptor agonists were not reimbursed in Croatia and only became available in 2024. Attempted pharmacological and non-pharmacological (various diets) methods yield no effect so she was referred to abdominal surgeon for consultations regarding bariatric surgery. In January 2023 she underwent sleeve gastrectomy (resection of a significant portion of the stomach and shaping the remaining part into a narrow, tube-like sleeve). Prior to surgery, the patient received short-term prophylaxis with recombinant human C1 esterase inhibitor. She experienced an uncomplicated post-operative recovery and, over a two-year period, achieved a weight reduction to 81 kg, reducing BMI to 28.70 kg/m2, placing her in the overweight category. Amelioration of attacks was observed following significant weight loss, accompanied by improved emotional status and a reduction of triggers.

**Conclusion:** We highlight the critical role of adequate short-term prophylaxis in the pre-procedural management of HAE patients undergoing attack-triggering interventions, including surgical procedures. Notably, this case represents, to our knowledge, the first reported instance of bariatric surgery in an HAE patient resulting in such significant weight reduction. Consent to publish had been obtained.

## O-48 Hereditary angioedema and autoimmune thyroiditis in a preadolescent female

### Gabriel E. Arce-Estrada^1,*^, Sara E. Espinosa-Padilla^1^, Francisco A. Contreras-Verduzco^2^, Nieto-Martínez Sandra^3^, David Monterrosas-Ustarán^4^

#### ^1^Primary Immunodeficiencies Laboratory, Instituto Nacional de Pediatría, Mexico City, Mexico; ^2^Allergy Service, Instituto Nacional de Pediatría, Mexico City, Mexico; ^3^Nutrition Genetics Unit, Instituto Nacional de Pediatría, Mexico City, Mexico; ^4^Division of Radiology and Imaging, Instituto Nacional de Pediatría, Mexico City, Mexico

##### **Correspondence:** Gabriel E. Arce-Estrada (arce.emmanuel@gmail.com)

***Allergy, Asthma & Clinical Immunology*** 2025, **21(Suppl 2)**:O-48

**Background:** Patients with hereditary angioedema (HAE) are at an increased risk of developing autoimmune diseases (OR 1.65; 95% CI 1.15–2.35). Autoimmune thyroiditis (AT) has a global prevalence of approximately 2%, with women being disproportionately affected, especially between the ages of 45 and 55 years [1,2]. However, there is limited evidence regarding the development of AT in patients with HAE.

**Case report:** We present a Mexican preadolescent female with a family history of HAE (mother and maternal uncle with a pathogenic variant in SERPING1 [del:chr11-57369598–57369642]). Both her great-grandfather and his maternal grandfather died due to idiopathic asphyxia and they had recurrent angioedema atacks.

At two months of age, the patient was admitted with symptoms resembling "whooping cough syndrome," which resolved with the administration of C1-inhibitor (C1-INH) due to suspected laryngeal edema. Subsequently, she experienced recurrent episodes of angioedema (affecting the face, eyelids, nasal area, and extremities), abdominal pain, dyspnea, cough, and syncope. These episodes led to a diagnosis of HAE based on low C4 and reduced C1-INH. She required multiple treatments with C1-INH and icatibant acetate, resulting in symptomatic improvement. The patient is currently awaiting genetic testing results.

At nine years of age, she presented with abdominal pain, respiratory distress, palpitations, and a grade III goiter. Laboratory findings revealed elevated thyroid-stimulating hormone, free thyroxine at the lower limit of normal, and positive antithyroglobulin and antiperoxidase antibodies. Ultrasound findings were consistent with thyroiditis. A diagnosis of AT was made in the hypothyroid phase, with a favorable response to levothyroxine therapy.

**Conclusions:** This case involves a female patient at the onset of adolescence, a period marked by increased estrogen production. Estrogen promotes the activation and maturation of B cells, which enhances the production of both antibodies and autoantibodies. Additionally, HAE independently disrupts complement pathways (both classical and lectin), impairing the clearance of apoptotic cells and immune complexes, thereby increasing exposure to autoantigens and the risk of autoimmunity [3].

In conclusion, both the onset of adolescence and the presence of HAE may have contributed to the development of AT in our patient, which contrasts with previous cases typically observed in adults. Informed consent was obtained from the patient's mother for the publication of this case.

**References**

1. Sundler Björkman L, Persson B, Aronsson D, Skattum L, Nordenfelt P, Egesten A. Comorbidities in hereditary angioedema-A population-based cohort study. Clin Transl Allergy. 2022 Mar;12(3):e12135. 10.1002/clt2.12135. PMID: 35,344,299; PMCID: PMC8967273.
2. Kaur J, Jialal I. Hashimoto Thyroiditis. 2025 Feb 9. In: StatPearls [Internet]. Treasure Island (FL): StatPearls Publishing; 2025 Jan–. PMID: 29,083,758.
3. McMurray JC, Schornack BJ, Weskamp AL, Park KJ, Pollock JD, Day WG, Brockshus AT, Beakes DE, Schwartz DJ, Mikita CP, Pittman LM. Immunodeficiency: Complement disorders. Allergy Asthma Proc. 2024 Sep 1;45(5):305–309. 10.2500/aap.2024.45.240050.

## O-49 Is angioedema a clinical mani-festation of immunodeficiency? Angioedema in heterozygous mutation of the PIK3R1 gene

### Vania M. Miranda-Saavedra^1,*^, Sara E. Espinosa-Padilla^1^, Francisco A. Contreras Verduzco^2^, Saúl Oswaldo Lugo Reyes^1^

#### ^1^Unidad de Investigación de Inmunodeficiencias, Instituto Nacional de Pediatría, Mexico City, Mexico; ^2^Servicio de Alergia, Instituto Nacional de Pediatría, Mexico City, Mexico

##### **Correspondence:** Vania M. Miranda-Saavedra (vaniamiranda211@gmail.com)

***Allergy, Asthma & Clinical Immunology*** 2025, **21(Suppl 2)**:O-49

**Introduction:** Activated Phosphoinositide 3-Kinase Delta Syndrome (APDS) is an autosomal dominant combined primary immunodeficiency caused by mutations in the PI3KCD or PIK3R1 genes. Some rare autosomal recessive loss-of-function mutations in PIK3R1 are associated with agammaglobulinemia; however, the clinical manifestations and immunological phenotypes of diseases related to the PI3K/AKT/mTOR pathway are diverse [1]. A patient with APDS is presented, whose clinical picture was centered on autoimmune and allergic manifestations, with angioedema triggered by sun exposure and peanut consumption being predominant.

**Case Presentation:** A 16-year-old female patient was assessed by the immunology department due to a history of headaches, weakness, oral ulcers, and malar erythema. She had a positive result for antinuclear antibodies with a coarse speckled +  + pattern. Suspecting Systemic Lupus Erythematosus, an autoimmune approach was initiated. Immunological studies showed normal results (C3, C4, IgG, IgM, IgA), and antibodies were negative (ENA 6 breakdown, SAF profile). During follow-up, a myeloproliferative syndrome was ruled out due to recurrent bleeding. Upon the appearance of angioedema related to sun exposure and peanut consumption, C1q, C1-inhibitor, and total IgE levels were all reported as normal. Allergic sensitization was ruled out with negative skin tests and CRD for aeroallergens. Given the persistence of arthralgia, a family history of autoimmunity, and ANA +  + results, an exome sequencing test was performed, which identified the mutation c.398 T > A (p.Ile133Asn) in the PIK3R1 gene, suggesting a diagnosis of primary immunodeficiency APDS associated with the heterozygous mutation on chromosome 5q13.

**Discussion:** The low incidence of APDS highlights a significant gap between the onset of symptoms and diagnosis, ranging from 2 to 9 months, particularly in cases with homozygous defects in PIK3R1. Clinically, 1% of patients present with angioedema with normal C1-inhibitor levels, 58.6% show signs of autoimmunity and inflammation, and 25% are ANA positive [1].

In the pathogenesis of angioedema, the PI3K signaling pathway regulates angiogenesis and endothelial cell proliferation through the basic fibroblast growth factor, as well as reduced activity of the RhoA/ROCK axis and stabilization of adherens junctions. The binding of vascular endothelial growth factor (VEGF) to its receptors (VEGFR1-3) induces tyrosine phosphorylation, activating signaling cascades such as PLC-γ and PI3K, promoting cell proliferation, survival, vasodilation, hyperpermeability, and cell migration [2]. Therefore, a mutation in the PI3K signaling pathway correlates with improper cytoskeletal rearrangement and endothelial hyperpermeability, suggesting angioedema as a characteristic clinical manifestation of this immunodeficiency [3]. Consent to publish had been obtained.

**References**

1. Bildik, H. N., Esenboga, S., Halaclı, S. O., Karaatmaca, B., Aytekin, E. S., Nabiyeva, N., Akarsu, A., Ocak, M., Erman, B., Tan, C., Arikoglu, T., Yaz, I., Cicek, B., Tezcan, I., & Cagdas, D. (2024). A single center experience on PI3K/AKT/MTOR signaling defects: Towards pathogenicity assessment for four novel defects. Pediatric Allergy and Immunology: Official Publication of the European Society of Pediatric Allergy and Immunology, 35(9), e14245. 10.1111/pai.14245
2. Debreczeni, M.L., Németh, Z., Kajdácsi, E. et al. Molecular Dambusters: What Is Behind Hyperpermeability in Bradykinin-Mediated Angioedema?. Clinic Rev Allerg Immunol 60, 318–347 (2021). 10.1007/s12016-021-08851-8
3. Di Lorenzo A, Lin MI, Murata T, Landskroner-Eiger S, Schleicher M, Kothiya M, Iwakiri Y, Yu J, Huang PL, Sessa WC (2013) eNOS-derived nitric oxide regulates endothelial barrier function through VE-cadherin and Rho GTPases. J Cell Sci 126(Pt 24):5541–5552. 10.1242/jcs.115972

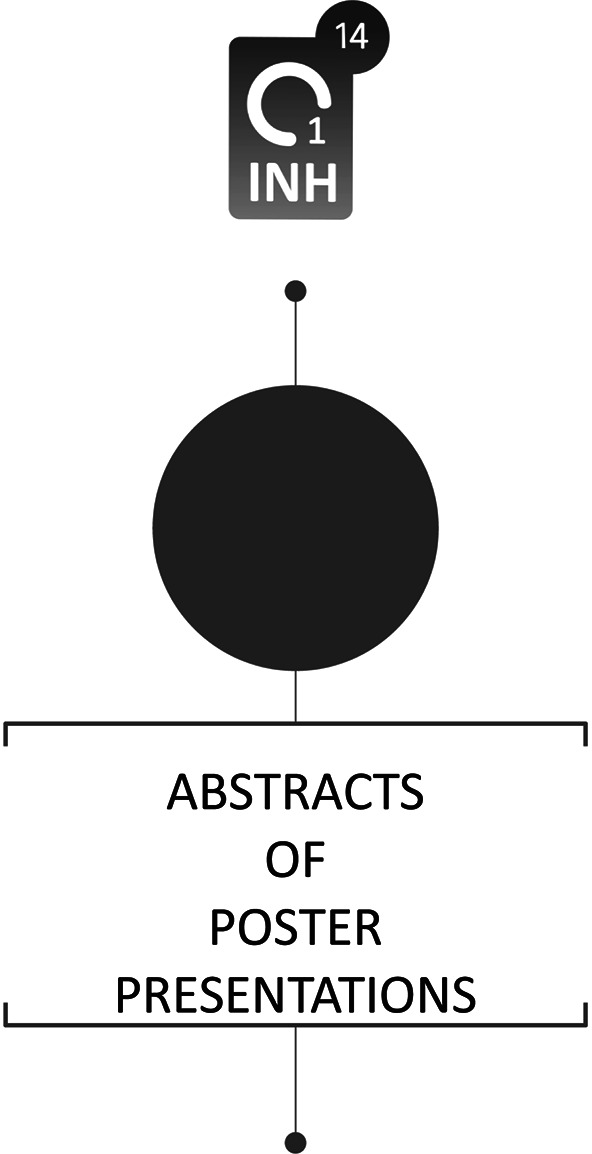


## P-01 Hereditary angioedema with normal C1-INH and PLG muta-tion treated with tranexamic acid: Beware of the risk of thrombosis

### Alexis Bocquet, David Launay, Isabelle Boccon-Gibod, Aurélie Du-Thanh, Delphiine Gobert, Sébastien Sanges, Laurence Bouillet

#### Launay &Sanges University of Lille, U1286-INFINITE-Institute for Translational Research in Inflammation, F-59000, INSERM, and the Department of Internal Medicine and Clinical Immunology, CHU Lille, National Reference Center for Angioedema (CREAK), Lille, France

***Allergy, Asthma & Clinical Immunology*** 2025, **21(Suppl 2)**:P-01

**Introduction:** The anti-fibrinolytic, tranexamic acid (TA) can be proposed as a long term prophylaxis for hereditary angioedema (HAE), and seems to be specifically efficient for HAE with plasminogen gene mutation (HAE-PLG), a subset of HAE with normal C1-INH (HAE-nC1-INH). Although TA is usually well tolerated in patients without any thromboembolic risk factors,we report here 4 cases of thrombosis following the use of TA in HAE-PLG patients.

**Methods:** retrospective study based on clinical data from the French HAE-nC1-INH registry.

**Results:** Of the 28 HAE-PLG patients recorded in France in December 2024, 20 were symptomatic and 10 were receiving long term prophylaxis, including 7 with TA. Of these, 4 have developed venous or arterial thrombotic complications. All patients received a usual dosage of 2 to 3 g/d of TA. For all patients, TA was totally effective in preventing AE: the frequency decreased from an average of 2 attacks per month before TA, to 0 attacks per month during TA.

**Conclusion:** The HAE-PLG patients who developed thrombotic events on AT were all elderly (over 65) and had cardiovascular history and risk factors. For 3 of them, the thrombotic event occurred within a year of the introduction of AT, despite prior antiplatelet aggregation. Four out of 7 (57%) of HAE-PLG patients taking TA had a thrombotic episode. TA administration should be avoided in patients with risk factors or a history of cardiovascular disease. This also raises the question of the role of the *PLG* mutation in this increased vascular risk.

**Table:** 

Age (years) and sex at time of thrombotic event	Cardiovascular risk factors and history	Antiplatelet aggregation therapy prior to TA initiation	Duration of TA treatment prior to thrombotic event	Cardio vascular events
Woman, 73	Hypertension Diabetes	No	1 year	Stroke
Woman, 95	Diabetes	Yes (during 25 years)	5 years	Heart attack
Man, 70	Ischemic heart disease	Yes (during 13 years)	1 year	Deep Venous thrombosis New episode of coronary ischemia
Man, 67	Peripheral arteriel disease	Yes (during 11 years)	1 year	Deep Venous thrombosis

## P-02 Challenges of diagnosis and treatment of recurrent angioedema from Nepal- the unseen struggle in resource-constrained settings

### Dharmagat Bhattarai^1^, Aaqib Zaffar Banday^2^, Apar Pokharel^3^, Asmita Neupane^1^

#### ^1^Advanced Centre for Immunology & Rheumatology, Kathmandu, Nepal; ^2^Department of Pediatrics, Government Medical College Srinagar, Srinagar, Kashmir, India; ^3^Civil Hospital, Kathmandu, Nepal

***Allergy, Asthma & Clinical Immunology*** 2025, **21(Suppl 2)**:P-02

Rationale: Angioedema is often missed or mistreated in a less privileged world. With the availability of a single immunologist, the etiological diagnosis of recurrent angioedema (RAE) is rising. We describe the challenges and struggles of diagnosing cases of RAE.

Methods: Case sheets of patients diagnosed (as per internationally acclaimed criteria) as various etiologies of RAE from August 2020 to February 2024 were analyzed. We also implemented community-directed interventions (CDIs) from January 2022 up to May 2023, like health camps, media promotions, articles, videos, television interviews, awareness talks, college classes, and society formation.

Results: RAE was mistreated as urticaria/allergy with antihistamines. Among > 100 cases with RAE, only 39 patients could do the biochemical tests and 10 could afford commercially available genetic tests. Among 39, Angiotensin-converting enzyme inhibitor- and ibuprofen-related acquired angioedema were diagnosed in 6 and 3 patients, respectively. 21 patients were diagnosed with hereditary angioedema with median age of onset and diagnosis of 8.5 and 23 years, respectively. Eighteen of them had low C4 and C1-esterase inhibitor (C1-INH). One patient had elevated C1-INH whereas the remaining one had normal C1-INH. Among 10 genetic diagnoses, 8 had a mutation in the SERPING1 gene (e.g., p. Gly17Arg, p.Arg494Ter) and 1 each in rare kininogen (KNG1) gene (p.Met379Lys) and 1 in MYOF gene (p.Met1923Thr). Among phenocopies, a girl had autoantibody to C1-inhibitor whereas another was found to have autoantibodies to factor H. In a web-based survey, HAE awareness among physicians was low. All patients with RAE were treated with antihistamines and steroids before visiting us. After CDIs, the rate of visiting patients with RAE doubled. Post-diagnosis, all patients were kept on long-term prophylaxis with tranexamic acid and/or attenuated androgens. Patients with laryngoedema or tongue swelling were also treated with fresh-frozen plasma infusions. One patient has recently availed of C1-INH therapy for her treatment. Others could not procure it due to non-affordability. Occasional angioedema accompanied urticaria in 14.5% (n = 40) of patients.

Conclusion: This is the first report on RAE from Nepal. Lack of awareness and resources have resulted in misdiagnosis, mistreatment, and poor outcomes in resource-constrained settings.

## P-03 Why are some HAE patients more resistant to prophylaxis than others?

### Shira Benor, David Hagin

#### Tel Aviv Souraski Medical Center, Tel Aviv, Israel

***Allergy, Asthma & Clinical Immunology*** 2025, **21(Suppl 2)**:P-03

**Background:** Patients with Hereditary angioedema (HAE) suffer attacks of swelling, which may involve the external and internal organs. The availability of effective prophylactic treatments for HAE with anti-Kallikreins such as Lanadelumab or Berotralstat is a groundbreaking improvement in health and quality of life for patients with frequent or severe attacks reducing attack rates by 50–95%. However, some patients continue to experience attacks despite prophylactic treatment. Our aim was to characterize this “prophylactic resistant” patient group.

**Methods:** A retrospective chart review of the patient database of the HAE clinic at the Tel Aviv Medical Center. Resistant disease was defined as regularly having more than one monthly attack on steady state of prophylactic treatment with Berotralstat or Lanadelumab or an inability to decrease frequency of biological prophylactic treatment with Lanadelumab from twice to once monthly due to breakthrough attacks. Patients with resistant disease were compared to the overall patient population.

**Results:** Sixty-four patients were included in the analysis. Thirty thirty-two were on Berotralstat or Lanadelumab. Of those, eleven were resistant to anti-Kallikrein prophylaxis, this is consistent with 70% attack free rates reported in studies on anti-kallikrein prophylaxis. Female gender, high pre-prophylaxis attack frequency, predominant abdominal attacks and Berotralstat treatment were associated with resistance to prophylaxis.

**Conclusion:** HAE patients with the above-mentioned features may need higher doses, a higher frequency of anti-kallikrein treatment or other prophylactic options.

## P-04 Hereditary angioedema due to factor XII mutation: Clinical manifestations among males subjects

### Delphine Gobert^1,*^, Btisseme Ahouach^1^, Isabelle Boccon-Gibod^2^, Laurence Bouillet^2^, Aurélie Du-Thanh^3^, David Launay^4^, Ludovic Martin^5^, Gaelle Hardy^6^, Olivier Fain^1^

#### ^1^Sorbonne Université, internal medicine department, University Hospital Saint-Antoine, Assistance Publique-Hôpitaux de Paris, Paris, France; ^2^Internal medicine department, University Hospital, Grenoble, France; ^3^Dermatology department, University Hospital, Montpellier, France; ^4^Internal medicine department, University Hospital, Lille, France; ^5^Dermatology department, University Hospital, Angers, France; ^6^Department of molecular genetic, University Hospital, Grenoble, France

##### **Correspondence:** Delphine Gobert (delphine.gobert@aphp.fr)

***Allergy, Asthma & Clinical Immunology*** 2025, **21(Suppl 2)**:P-04

**Background:** Hereditary angioedema caused by mutation of factor XII (HAE-FXII) accounts for 20 to 25% of HAE with normal C1-inhibitor. They occur preferentially in women after puberty, under estrogen-containing contraception or during pregnancy. However, hormones do not appear to be the only factor triggering this type of angioedema, and a few series report cases of symptomatic male subjects. The aim of our study was to investigate the prevalence of angioedema symptoms in male subjects, and to describe their location, frequency, duration and response to treatment.

**Materials and methods:** We conducted a retrospective observational study of angioedema manifestations in males carrying FXII mutation at the angioedema reference center of Saint Antoine Hospital in Paris, and in patients screened by the reference center genetic laboratory, in Grenoble, between June 2006 and May 2020.

**Results:** We included 43 male subjects carriers of the FXII mutation. Of these, 19% (8/43) were symptomatic, whereas 89% of women were symptomatic in our cohort. The mean age of onset of symptoms is 29.5 years (6 to 68 years). The main localizations of angioedema attacks were facial oedema (50%) and abdominal pain (50%), then peripheral oedema (12%), laryngeal (12%) and tongue (12%) oedema. Angioedema attacks lasted 48 to 72 h, with a mean frequency frequency of 2.25 attacks per year. Response to treatment could not be evaluated in all patients; half of patients responded to icatibant or plasma derived C1-inhibitor concentrates.

**Conclusion:** Our work enabled us to identify symptomatic male carriers of the factor XII mutation screened in France between 2006 and 2021. Angioedema attacks are rare in this population, and age at first attack occurs late in life. The mechanism of angioedema in this population has not been confirmed in all subjects. Follow-up of these patients is therefore necessary information on the disease, its genetic transmission and its management, especially in male children.

**Acknowledgements** The French reference centers network od angioedema CREAK.

## P-05 Acquired angioedema: A case of recurrent angioedema for two years after a successful treatment and normalization of the C1-inhibitor level

### Sladjana Andrejevic, Radovan Mijanovic, Vojislav Djuric

#### Clinic of Allergy and Immunology, University Clinical Center of Serbia, Belgrade, Serbia

***Allergy, Asthma & Clinical Immunology*** 2025, **21(Suppl 2)**:P-05

**Background:** Angioedema due to acquired C1-inhibitor (C1-INH) deficiency (C1-INH-AAE) is a rare disorder caused by acquired consumption of C1-INH. A 2016 study reported that 33% of patients presenting with C1-INH-AAE had or would develop non-Hodgkin lymphoma (NHL), in particular splenic marginal zone lymphoma (SMZL).

**Case Report:** A female patient, presented at the age of 50 due to recurrent episodes of angioedema that affects the face, lips and extremities. Her family history was negative for angioedema. She had an appendectomy at age of 18 followed by recurrent episodes of abdominal pain and symptoms of intestinal occlusion. Complement testing showed a reduced levels of C4 < 0.05 g/L and C1-INH of 0.05 g/L (n.v. 0.19–0.58), with normal level of C1q of 0.098 g/L (n.v. 0.05–0.25). Normal number of leukocytes was determined and no monoclonal component was demonstrated by serum immunofixation. Unfortunately, at that point in time, we were not able to perform a genetic analysis of the SERPING1 gene, nor to determine the existence of antibodies against the C1-INH. The diagnosis of angioedema due to C1-INH deficiency was established, indefinite whether it was hereditary or acquired. In the further course, the patient continued to have swellings, which were successfully treated with the use of icatibant. A patient refrained from having regular checkups due to the COVID-19 pandemic. In 2022 she was referred to a hematologist after the detection of peripheral lymphocytosis and splenomegaly. Definitive diagnosis of SMZL was made after integration of bone marrow (BM) histology with cell morphology and immunophenotype in the blood and BM. The diagnosis of C1-INH-AAE was finally made. SMZL was treated with rituximab with cyclophosphamide, vincristine, doxorubicin, and prednisone (R-CHOP). After the treatment, the lymphocyte count rapidly decreased to normal levels. The spleen size returned to normal after 8 months. Control BM histology was negative for infiltration. Moreover, normal concentrations of both C4 of 0.23 g/L (n.v. 0.1–0.4) and C1-INH of 0.3 g/L were registered six months after the treatment. Regrettably, she did not experienced post-treatment reduction in AAE symptoms. During the past year she has experienced 7 moderate/severe attacks of angioedema. The last, severe abdominal attack occurred in January of this year, two years after completing treatment for NHL.

**Conclusion:** Finally, our case highlights that a patient formerly diagnosed with C1-INH-AAE can have a further recurrences of angioedema even after normalization of the C1-INH level.

Written informed consent for publication was obtained from the patient.

### P-06 The effect of surgical and interventional procedures on attack frequency in hereditary angioedema patients

#### Ecem Ay, Ragıp Fatih Kural, Emine Nihal Mete Gökmen, Su Ozgur

#### Ege University Faculty of Medicine, Department of Internal Medicine, Division of Immunology and Allergy, İzmir, Turkey

***Allergy, Asthma & Clinical Immunology*** 2025, **21(Suppl 2)**:P-06

**Introduction:** Hereditary angioedema (HA) is a condition characterized by recurrent angioedema symptoms affecting the skin, internal organs, and larynx in the form of attacks. HA attacks can occur spontaneously or be triggered by factors such as trauma, stress, and surgical interventions. Laryngeal edema can be life-threatening due to the risk of airway obstruction.

**Objective:** The objective is to determine the risk of attack development in HA patients undergoing surgical and medical interventions. This study aims to predict HA attacks based on the type of surgical intervention.

**Method:** The surgical and medical intervention histories of 63 patients diagnosed with C1-inhibitor deficiency-associated HA (HA-C1-INH) were retrospectively analyzed to evaluate post-procedure attack frequency. Invasive surgical procedures involving extensive resections, removal of organs through body cavities, or significant alterations to normal anatomical structures were classified as major surgeries. Limited invasive procedures involving the skin, mucous membranes, and connective tissues without significant changes to the anatomical structure were classified as minor surgeries. Non-invasive procedures that did not involve incisions or sutures were categorized as interventional procedures (Table 1).

**Table 1 (abstract P-06):**
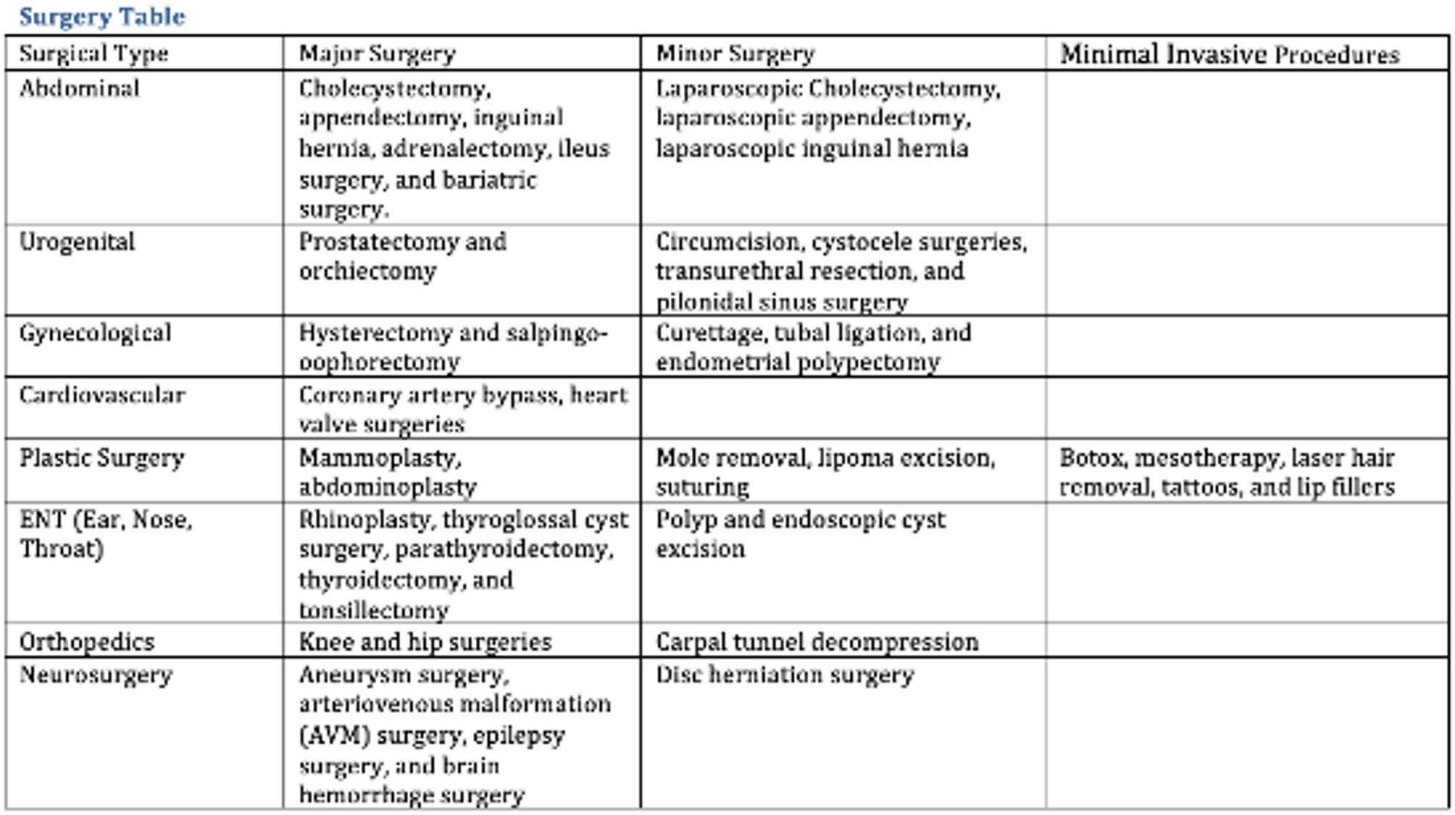
Major/minor surgeries and interventional procedures for our patients

**Results:** Among 63 patients diagnosed with HA-C1-INH, attacks occurred in 16 out of 33 major surgeries (48.5%). Attacks were observed in 12 out of 72 minor surgeries (16.7%). Attacks occurred in 5 out of 244 interventional procedures (2.05%; p < 0.0001) (Tables 2 and 3). The rate of HA attacks associated with minor surgeries and medical interventions was found to be significantly lower compared to major surgeries (p < 0.0001).

**Table 2 (abstract P-06):**
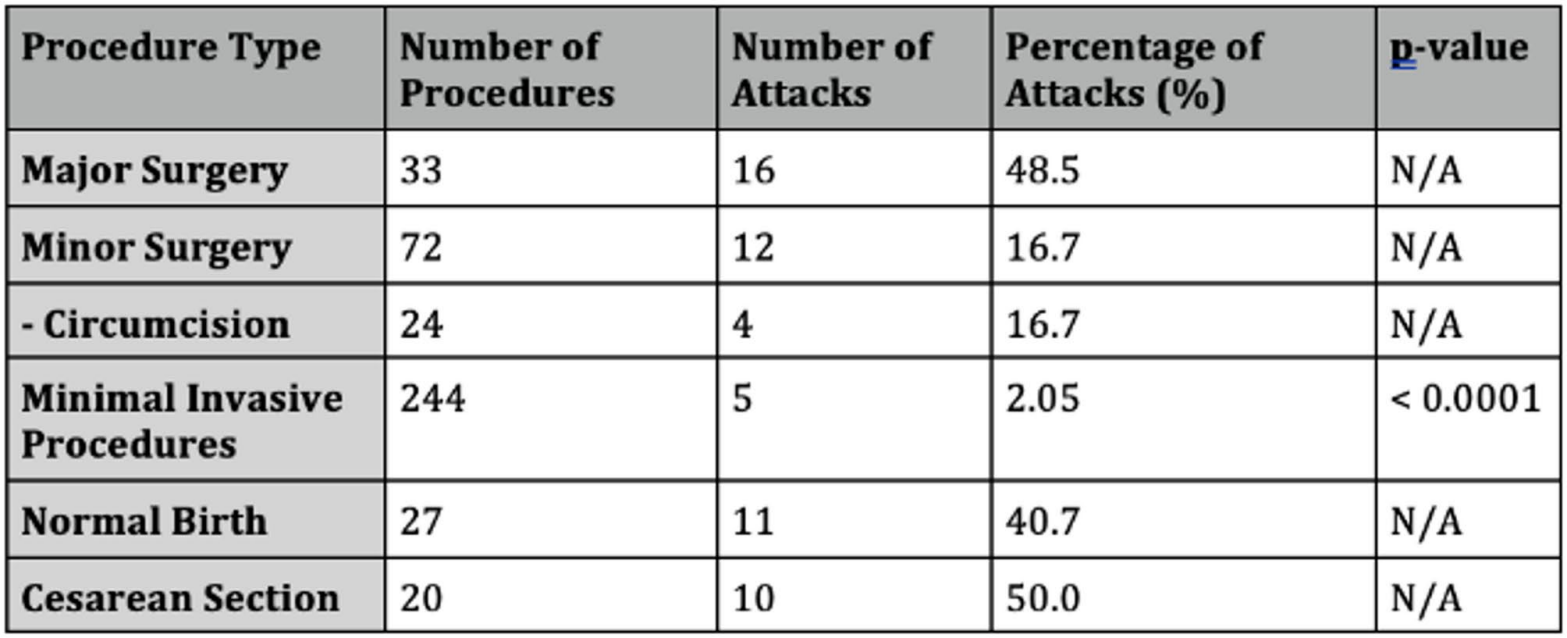
Relationship betweensurgeries and attack

**Table 3 (abstract P-06):**
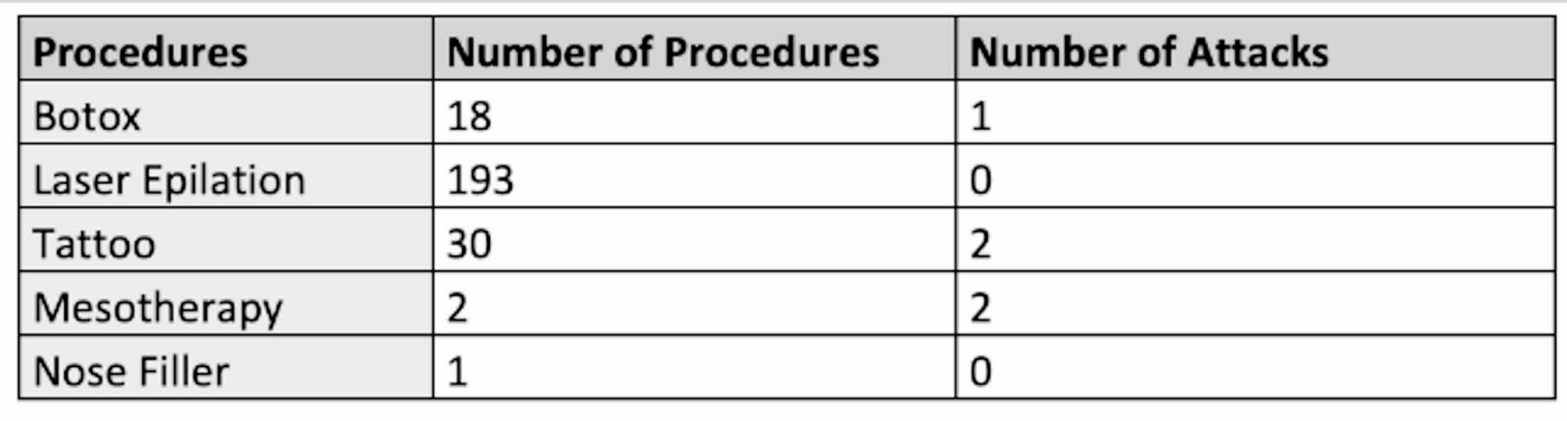
Relationship between interventional procedures and attacks

**Conclusion:** Surgical interventions, particularly major surgeries, can trigger HA attacks. Short-term prophylactic treatment is recommended, especially before major surgical operations. Minor surgeries and medical interventions carry a lower risk; therefore, although prophylactic treatment may not be necessary, acute attack treatment should be readily available (Fig. 1).

**Figure 1 (abstract P-06):**
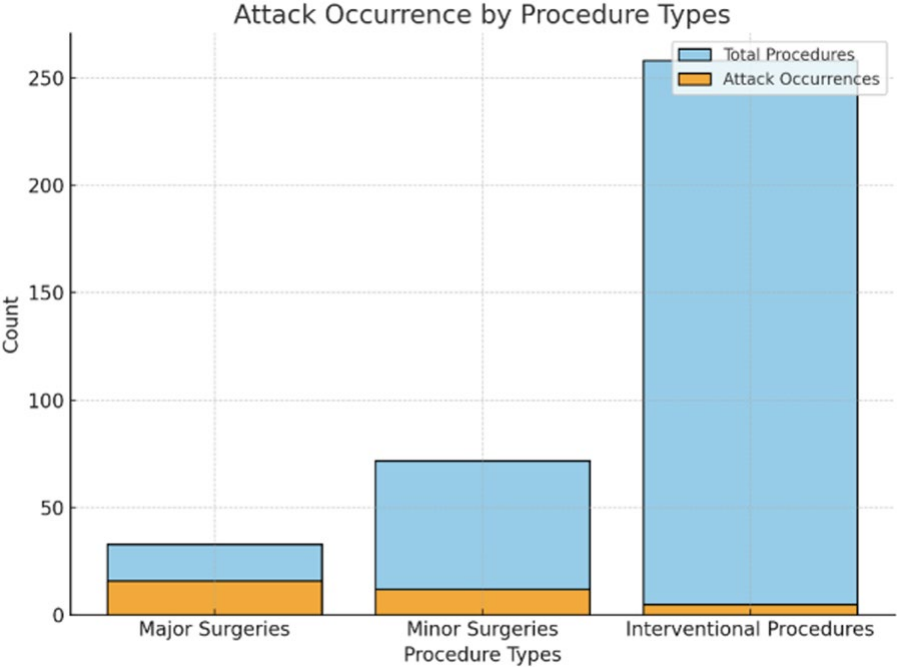
Relationship between surgeries and attack

**Keywords** C1-inhibitor deficiency, Hereditary angio-edema, Invasive surgery, Major surgery, prophylaxis

## P-07 Investigating the risks of long-term glucocorticoid and attenuated androgen use in patients with angioedema: A systematic literature review

### Emel Aygören-Pursun^1^, Noemi-Anna Bara^2^, Thomas Buttgereit^3,4^, Stefan Cimbollek^5^, Danny M. Cohn^6^, Henriette Farkas^7^, Sorena Kiani-Alikhan^8^, Markus Magerl^3,4*^, Johanna M. Mandelin^9^, Marc A. Riedl^10^, Sinisa Savic^11^, Marta Sobotková^12^, Marcin Stobiecki^13^, Andrea Zanichelli^14,15^

#### ^1^Center for Children and Adolescents, University Hospital Frankfurt, Frankfurt, Germany; ^2^Romanian Angioedema Center of Reference and Excellence, Centrul Clinic MediQuest, Sangeorgiu de Mures, Romania; ^3^Angioedema Center of Reference and Excellence (ACARE), Institute of Allergology, Charité – Universitätsmedizin Berlin, corporate member of Freie Universität Berlin and Humboldt-Universität zu Berlin, Berlin, Germany; ^4^Fraunhofer Institute for Translational Medicine and Pharmacology ITMP, Immunology and Allergology, Berlin, Germany; ^5^Angioedema Reference Center (CSUR), Allergy Department, Hospital U. Virgen del Rocío, Seville, Spain; ^6^Department of Vascular Medicine, Amsterdam UMC, Amsterdam Cardiovascular Sciences, University of Amsterdam, Amsterdam, The Netherlands; ^7^Hungarian Angioedema Center of Reference and Excellence, Department of Internal Medicine and Haematology, Semmelweis University, Budapest, Hungary; ^8^Department of Immunology, Royal Free London NHS Foundation Trust, London, UK; ^9^Department of Allergology, Skin and Allergy Hospital, Helsinki University Central Hospital, Helsinki, Finland; ^10^Division of Allergy and Immunology, University of California San Diego, La Jolla, California, USA; ^11^Clinical Immunology and Allergy, NIHR-Leeds Biomedical Research Centre, Leeds Teaching Hospitals NHS Trust, Leeds, UK; ^12^Department of Immunology, 2nd Faculty of Medicine Charles University and Motol University Hospital, Prague, Czech Republic; ^13^Department of Clinical and Environmental Allergology, Jagiellonian University Medical College, Krakow, Poland; ^14^Operative Unit of Medicine, Angioedema Center, IRCCS Policlinico San Donato, San Donato Milanese, Milan, Italy; ^15^Dipartimento di Scienze Biomediche per la Salute, Università degli Studi di Milano, Milan, Italy

##### **Correspondence:** Markus Magerl (markus.magerl@charite.de)

***Allergy, Asthma & Clinical Immunology*** 2025, **21(Suppl 2)**:P-07

**Background:** Angioedema (AE) is charac-terized by leakage of fluid into tissues resulting in localized swelling. It is often categorized by two known mechanisms: mast cell (AE-MC) or bradykinin-mediated (AE-BK). Management of AE is focused on symptom control. In AE-MC this is often achieved with antihistamines, monoclonal antibodies, glucocorticoids, but in AE-BK, drugs such as C1-INH, kallikrein inhibitors, and androgens are used. While treatment varies, adverse effects have been observed with long-term use of glucocorticoids and androgens. Here, we aimed to evaluate the risks and side effects of long-term glucocorticoid and androgen use in patients with AE.

**Methods:** A systematic literature review (SLR) was performed using PubMed, and publications related to long-term glucocorticoid and androgen use were identified using separate search strings. Literature reviews and publications that were written in non-English languages or included non-human data were excluded. Grant funding (#80019) for this SLR was provided to ACARE by BioCryst Pharmaceuticals Inc.

**Results:** Overall, 61 publications concerning the long-term use of glucocorticoids (n = 5) and androgens (n = 56) for the management of AE met the search criteria. Across the publications, patients were often prescribed glucocorticoids such as dexamethasone and prednisone, and androgens such as danazol and stanozolol. Long-term glucocorticoid and androgen use allowed some patients to experience improvements in their symptoms and quality-of-life. However, others experienced multisystemic adverse effects including abnormal liver function, indigestion, hirsutism, menstrual irregularities, behavioural changes and mood disorders (e.g., aggression, anxiety, depression), as well as cardiometabolic changes (e.g., elevated cholesterol, hypertension). Dosage adjustments and changes to therapeutic regimens were able to address certain adverse effects (e.g., depression, hirsutism, menstrual irregularities, weight gain). However, other more severe effects were irreversible and, in some cases, life threatening (e.g., *hepatocellular carcinoma*).

**Conclusions:** The findings from this SLR underscore the need to minimize use of glucocorticoids and androgens where possible and encourage the use of effective modern treatments to reduce the risk of long-term adverse effects. Where modern therapies are not yet available, physicians should aim to prescribe the lowest effective dose of glucocorticoids or androgens and closely monitor for side effects.

**Acknowledgements** Medical writing support was provided by Talisa Silzer, PhD, from Sixsense Strategy Group, Inc., a Herspiegel company, and was funded by ACARE. This work is dedicated to the memory of Professor Marcus Maurer, whose groundbreaking contributions to allergy research and dedication to scientific excellence have profoundly influenced this work.

## P-08 Unmet needs and systemic challenges of HAE pediatric patient families in Europe

### Camelia Isaic^1,*^, Alyssa Sutormina^2^

#### ^1^HAE Junior patient organization, Czech Republic; ^2^Semantic Hub Oy, Helsinki, Finland

##### **Correspondence:** Camelia Isaic (cisaic@haejunior.cz)

***Allergy, Asthma & Clinical Immunology*** 2025, **21(Suppl 2)**:P-08

**Introduction:** The recent therapeutic innovations and improving disease awareness levels in Europe have led to a better quality of life of nowadays hereditary angioedema (HAE) patients when compared to earlier generations. In spite of this progress, practical experience indicates that the youngest HAE patient community still faces a high level of unmet needs and systemic challenges.

**Objective:** The objective of this study is to map out the current unmet needs and systemic challenges of HAE pediatric/young patient experience in Europe as the basis for future educational/ support activities.

**Materials and methods:** Between October 2024 and February 2025 the authors conducted an AI-powered multilingual natural language processing analysis of 537 anonymised patient queries/ stories posted since 2019 on social media and other internet platforms. A total of 105 patients/ carers from 11 European countries (Germany, Poland, United Kingdom, Czech Republic, Greece, Cyprus, Serbia, Belgium, Croatia, North Macedonia, France) were analysed.

**Results:** The research highlights persisting critical gaps in diagnosis, treatment, and support for pediatric HAE patients and their families. Quality of life issues were mentioned by 78% of authors, barriers to diagnosis/ treatment were identified in 74% of cases, while informational unmet needs were highlighted by 35%. The most frequently mentioned QoL issues include family burden, mental health challenges, and the unpredictability of attacks. Key barriers involve diagnostic challenges, lack of specialist support, and difficulties in accessing treatment. Among informational unmet needs, the most commonly cited topics were symptom management and treatment options, application & adjustment.

When considered from a geographical perspective, the results indicate that while young HAE patients and/ or their caregivers in Western Europe have to deal mostly with bureaucratic and insurance hurdles, their Central and Southern European peers face more fundamental access issues, such as regional disparities in availability of specialised healthcare and treatment options.

**Conclusions:** The findings show that the experience of European pediatric/ young HAE patients and their families implies significant health, emotional and financial burdens. Addressing biases, improving treatment access and convenience, upgrading emergency response and providing holistic family care are essential to further enhancing the quality of life for pediatric/ young HAE patients and their caregivers in Europe.

## P-09 Exploring the impact of psychological burden in hereditary angioedema with the pictorial representation of illness and self-measure (PRISM) tool

### Delphine Gobert^1,*^, Mélanie Javaud^1^, Enzo Cohen^2^, Olivier Fain^1^, Boris Bienvenu^3^

#### ^1^Sorbonne Université, French National Reference Center for Angioedema (CREAK), Department of Internal Medicine, Saint Antoine Hospital, Paris 6 University, Paris, France; ^2^BioCryst Pharmaceuticals France, Medical Affairs Department, Boulogne-Billancourt, France; ^3^Department of Internal Medicine, 15-20 National Vision Hospital, Paris, France

##### **Correspondence:** Delphine Gobert (delphine.gobert@aphp.fr)

***Allergy, Asthma & Clinical Immunology*** 2025, **21(Suppl 2)**:P-09

**Background:** Hereditary angioedema (HAE) significantly affects patients' quality of life, imposing physical and psychological burdens. While both the Angioedema Quality of Life (AE-QoL) and Angioedema Control Test (AECT) questionnaires are validated tools for assessing quality of life and disease strain, they may not fully capture the psychosocial dimension. Our objective is to investigate a Pictorial Representation of Illness and Self Measure (PRISM) instrument, a tool that simplifies the visual evaluation of psychological burden and emotional insights, in its first use within the context of HAE.

**Materials and methods:** This exploratory, on a given day of routine visit, monocentric study involves HAE patients. The PRISM tool assesses psychological burden through Self-Illness Separation (SIS) and Self-Medical Care Separation (SMcS) scores, reflecting the perceived impact of illness and medical care (higher distances indicate lower burden; range: 0–27 cm). Patient narratives (verbatim) are also collected during educational sessions. Additional measures include the AE-QoL (higher scores indicate worse quality of life; range: 0–100) and AECT (higher scores indicate better disease control; range: 0–16) for burden of disease.

**Results:** Of the 17 patients recruted, 70.6% were female, with a mean age of 32.8 years (SD ± 12.3). Most patients (78%) had HAE-C1-INH, and 53% were on long-term prophylaxis (LTP; 4 berotralstat, 3 Lanadelumab and 2 danazol) at the time of evaluation. In this first pool of patients, mean scores were as follows: AE-QoL: 21.2 (SD ± 14.8), AECT: 8.8 (SD ± 3.99), PRISM SIS: 10.85 (SD ± 6.53), and PRISM SMcS: 11.57 (SD ± 7.83) reflecting a moderate overall disease burden. The PRISM SIS score showed a statistically significant negative correlation with AE-QoL (r = -0.55, p = 0.022), indicating that improved quality of life is associated with reduced psychological burden. PRISM scores did not correlate with AECT scores. Patients without LTP reported higher psychological burden. Lanadelumab and berotralstat were perceived similarly in their impact. Analysing the patients' verbatim provided interesting insights into how they perceived the treatment and how they dealt with the disease.

**Conclusions:** The PRISM tool is an effective method for assessing the psychological burden of HAE. It offers a user-friendly alternative to traditional questionnaires, strongly correlates with quality-of-life outcomes, and encourages patients to engage in open discussion with their physician. PRISM may complement existing PRO measures by enhancing patient management and understanding of treatment impacts. Further studies are needed to validate its use in HAE patients in routine clinical practice.

## P-10 Lanadelumab leads to meaningful quality of life improvement and reduced productivity loss in patients with HAE: Final results from the ENABLE Study

### Emel Aygören-Pürsün^1^, Mar Guilarte^2^, Mona Al-Ahmad^3^, Andreas Recke^4^, Karin Hartmann^5,6,7^, Maureen Watt^8^, Daniel Nova Estepan^8^, Irmgard Andresen^9^*, Natalie Khutoryansky^8^, Aharon Kessel^10^

#### ^1^Department for Children and Adolescents, Angioedema Centre, University Hospital Frankfurt, Goethe University Frankfurt, Frankfurt, Germany; ^2^Department of Allergy, Vall d'Hebron University Hospital, Vall d’Hebron Research Institute (VHIR), Barcelona, Spain; ^3^Microbiology Department, College of Medicine, Kuwait University, Kuwait City, Kuwait; ^4^Department of Dermatology, Allergology and Venereology, University of Lübeck, Lübeck, Germany; ^5^Division of Allergy, Department of Dermatology, University Hospital Basel and University of Basel, Basel, Switzerland; ^6^Department of Clinical Research, University of Basel and University of Basel, Basel, Switzerland; ^7^Department of Biomedicine, University Hospital Basel and University of Basel, Basel, Switzerland; ^8^Takeda Development Center Americas, Inc., Lexington, Massachusetts, USA; ^9^Takeda Pharmaceuticals International AG, Zurich, Switzerland; ^10^Division of Allergy and Clinical Immunology, Bnai Zion Medical Centre, Technion Faculty of Medicine, Haifa, Israel

##### **Correspondence:** Irmgard Andresen (irmgard.andresen@takeda.com)

***Allergy, Asthma & Clinical Immunology*** 2025, **21(Suppl 2)**:P-10

**Background:** The real-world effectiveness of long-term Lanadelumab treatment in patients with hereditary angioedema (HAE) was evaluated in the Phase 4 non-interventional, prospective ENABLE Study. One of the study objectives was to describe patient-reported outcomes (PROs).

**Materials and methods:** Patients aged ≥ 12 years with HAE who initiated Lanadelumab treatment per approved product labelling were enrolled to the ENABLE Study (NCT04130191). PROs in ENABLE were collected at pre-Lanadelumab baseline, Months 1, 2, 3 and 6, and every 6 months thereafter until the end of follow-up (up to 36 or 24 months if enrolled before or on/after 1 March 2021, respectively). PROs included the Angioedema Quality of Life (AE-QoL; clinically meaningful improvement is defined as ≥ 6-point decrease in the AE-QoL total score; ≥ 39 points in the AE-QoL total score represent moderate to large health-related quality-of-life [HRQoL] impairment), the Hospital Anxiety and Depression Scale (HADS) and the Work Productivity and Activity Impairment: General Health (WPAI-GH; in adults only) questionnaires.

**Results:** In ENABLE, 138 patients (mean ± SD age, 41.0 ± 14.4 years; female, 62,3%; HAE-C1-INH-Type1, 92.0%) received ≥ 1 Lanadelumab dose. On average, patients reported a moderate to large HRQoL impairment at baseline as measured by the AE-QoL total score ≥ 39 (the AE-QoL total score [mean ± SD], 42.9 ± 18.2; n = 138). A clinically meaningful HRQoL improvement as measured by AE-QoL was achieved by Month 1 after Lanadelumab initiation (the AE-QoL total score [mean ± SD], 29.1 ± 19.9; n = 107) and persisted until the end of the study. Improvements from baseline were also observed in the mean AE-QoL Functioning, Fatigue/Mood, Fears/Shame and Nutrition scores. Numerical decreases in the HADS Anxiety and Depression subscale scores (mean ± SD) were observed from baseline (7.6 ± 4.3 and 5.3 ± 4.0, respectively; n = 138) to Month 24 (5.2 ± 4.4 and 4.0 ± 4.1, respectively; n = 116) and Month 36 (4.7 ± 4.4 and 3.2 ± 3.7, respectively; n = 48). Overall work productivity loss and activity impairment as measured by WPAI:GH decreased by Month 1 of Lanadelumab treatment, to 19.0 ± 27.7 (mean ± SD) and 27.1 ± 30.5, respectively (n = 100), from 39.5 ± 31.4 and 41.3 ± 28.9, respectively, at baseline (n = 130); decreases persisted until the end of the study.

**Conclusions:** Real-world findings in patients with HAE starting Lanadelumab treatment show notable and sustained improvements in HRQoL, less anxiety and depression, reduced work productivity loss and less activity impairment, reinforcing the benefit of long-term prophylaxis with Lanadelumab.

Trial registration: Clinicaltrials.gov, NCT04130191.

**Acknowledgements** The ENABLE Study and medical writing assistance for this abstract were sponsored by Takeda Development Center Americas, Inc., Lexington, MA, USA. The interpretation of the data was made by the authors independently.

## P-11 Infection rates in patients with hereditary angioedema treated with long-term prophylaxis with garadacimab

### Mauro Cancian^1^, Hilary Longhurst^2^, Paul K. Keith^3^, Henriette Farkas^4^, Harsha Shetty^5^, Maressa Pollen^5^, Henrike Feuersenger^6^, Jonathan A. Bernstein^7^

#### ^1^UOSD Allergologia, University Hospital of Padua, Padua, Italy; ^2^Auckland City Hospital and The University of Auckland, Auckland, New Zealand; ^3^McMaster University Medical Centre, Hamilton, ON, Canada; ^4^Department of Internal Medicine and Haematology, Hungarian Angioedema Center of Reference and Excellence, Semmelweis University, Budapest, Hungary; ^5^CSL Behring, King of Prussia, PA, USA; ^6^CSL Innovation GmbH, Marburg, Germany; ^7^Department of Internal Medicine, Division of Rheumatology, Allergy and Immunology, University of Cincinnati, and the Bernstein Clinical Research Center Cincinnati, Cincinnati, OH, USA

***Allergy, Asthma & Clinical Immunology*** 2025, **21(Suppl 2)**:P-11

**Background:** Hereditary angioedema (HAE) is characterized by unpredictable, recurrent, and potentially life-threatening attacks of swelling. Garadacimab, (fully human anti-activated factor XII [FXIIa] monoclonal antibody) has demonstrated a favorable long-term safety profile and durable efficacy across the clinical program, with 62% and 50% of patients in the pivotal Phase 3 study and Phase 3 open-label extension study (OLE), respectively, achieving attack-free status. As a key initiator of the contact system, FXIIa is also involved in the regulation of the immune system. Evaluating the occurrence of infections during long-term prophylaxis with garadacimab against HAE is therefore important as part of the characterization of its safety profile. Here we describe immunity-related TEAEs throughout the clinical program.

**Materials and methods:** Data was collected from an integrated analysis of placebo-controlled Phase 2 and pivotal Phase 3 studies and their respective OLE studies in HAE of garadacimab 75/200/400/600 mg subcutaneous (SC) (Phase 2 only), 200 mg SC once-monthly or volume-matched placebo. An assessment of the occurrence of infections (defined as infections and infestations per Medical Dictionary for Regulatory Activities) was performed. Laboratory values relating to immune function were also evaluated.

**Results:** Overall, 122/172 patients (70.9%) receiving garadacimab over a median exposure of 2.5 years experienced infections at a rate of 0.853 per patient-year compared with 10/33 (30.3%) patients with placebo (median exposure 0.5 years) experiencing an infection rate of 0.954 per patient-year. No patients treated with placebo and 2 patients treated with garadacimab experienced serious infections (2 cases of COVID-19). None of the reported infections were related to garadacimab. The most common infections with a yearly rate > 0.03 were COVID-19 (0.176 with garadacimab; 0.220 with placebo), nasopharyngitis (0.180; 0.073), upper respiratory tract infection (0.063; 0.293), oral herpes, (0.048; 0.073) sinusitis (0.038; 0.000), influenza (0.036; 0.000) and urinary tract infection (0.033; 0.073).

Leukocyte and neutrophil mean cell counts remained within normal range between screening, baseline and throughout the pivotal Phase 3 (leukocytes: normal range 4.0–10.0 × 109/L mean count at Day 182: 6.6 × 109/L with garadacimab [n = 39], 6.2 × 109/L with placebo [n = 24], neutrophils: normal range 2.0–7.0 × 109/L, mean count at Day 182: 4.0 × 109/L with garadacimab, 3.9 × 109/L with placebo) and OLE studies (At Month 12 [n = 140]: leukocytes: 6.4 × 109/L; neutrophils: 4.0 × 109/L) for both garadacimab and placebo.

**Conclusions:** Patients with long-term prophylaxis with garadacimab showed a comparable pattern of infections and leukocyte and neutrophil counts to placebo.

## P-12 NTLA-2002 mechanism of action, pharmacology, safety, and efficacy in hereditary angioedema

### Danny M. Cohn^1,*^, Aleena Banerji^2^, Joshua S. Jacobs^3^, Allen P. Kaplan^4^, Andrea Zanichelli^5,6^, James S. Butler^7^, David Maag^7^, Catherine Miller^7^, Jonathan A. Phillips^7^

#### ^1^Department of Vascular Medicine, Amsterdam Cardiovascular Sciences, Amsterdam University Medical Center, University of Amsterdam, Amsterdam, The Netherlands; ^2^Allergy and Immunology, Department of Medicine, Harvard University and Massachusetts General Hospital, Boston, MA, USA; ^3^Allergy and Asthma Clinical Research, Walnut Creek, CA, USA; ^4^Division of Pulmonary Medicine and Allergy and Immunology, Medical University of South Carolina, Charleston, SC, USA; ^5^Department of Biomedical Sciences for Health, University of Milan, Milan, Italy; ^6^Department of Medicine, Angioedema Center, IRCCS Policlinico San Donato, San Donato Milanese, Milan, Italy; ^7^Intellia Therapeutics, Cambridge, MA, USA

***Allergy, Asthma & Clinical Immunology*** 2025, **21(Suppl 2)**:P-12

**Background:** Hereditary angioedema (HAE) requires lifelong treatment to manage symptoms. HAE manifests as severe and unpredictable swelling attacks through dysregulated bradykinin production by kallikrein. NTLA-2002 is an investigational, in vivo CRISPR-based therapy designed to rebalance the kallikrein-kinin system by permanently editing the *KLKB1* gene to prevent attacks after a single administration. We describe the mechanism of action (MOA) of NTLA-2002 and its preclinical and clinical pharmacology, efficacy, and safety in HAE.

**Methods:** Nonclinical experiments were conducted in humanised murine models and nonhuman primates (NHPs) to assess pharmacokinetics, *KLKB1* editing, pharma-codynamics, and durability. Clinical pharmacokinetics, pharmacodynamics, efficacy, and safety were evaluated in a phase 1/2 clinical trial (NCT05120830) [1,2].

**Results:** In primary human hepatocytes, NTLA-2002 demonstrated dose-related increases in *KLKB1* editing with proportional decreases in messenger RNA (mRNA) and prekallikrein protein. Studies in NHPs showed lipid nanoparticles, guide RNA (gRNA), and Cas9 mRNA were rapidly cleared from blood and the liver within 6 h of infusion; gRNA required for editing was undetectable in the liver within 1–2 days. This brief exposure resulted in dose-dependent *KLKB1* editing in liver and correlating deep reductions in total plasma kallikrein. NTLA-2002-treated mice showed *KLKB1* edits persist through natural turnover and liver regeneration.

In the phase 1 study, single-dose NTLA-2002 led to durable reductions in HAE attacks. NTLA-2002 (25-75 mg; N = 10) reduced monthly attacks by 96.6%-99.5% from baseline, observed up to 104 weeks of follow-up (range, 72–104 weeks). In phase 2, NTLA-2002 25 mg (n = 10) and 50 mg (n = 11) reduced attacks by 75%-77% vs placebo (n = 3; weeks 1–16). At ≈1 year, 90% of all treated patients remained off long-term prophylaxis, with 8 patients who were treated with 50 mg attack-free. All adverse events were mild (grade 1/2), most commonly headache (38%), fatigue (29%), and nasopharyngitis (29%).

**Conclusion:** Our nonclinical studies demonstrate how a single, brief exposure to NTLA-2002 durably edits the *KLKB1* gene in hepatocytes, resulting in deep, persistent reductions in prekallikrein, even through partial liver resection, suggesting a permanent effect. In clinical studies, NTLA-2002 was well tolerated and led to durable reductions in HAE attacks, supporting the notion that editing *KLKB1* can lead to a positive effect on disease. Collectively, these data demonstrate the durable efficacy and safety of single-dose NTLA-2002 mediated through its precise MOA. NTLA-2002 is undergoing further clinical investigation in the phase 3, global, multicentre, randomised, double-blind, placebo-controlled HAELO study (NCT06634420) as potentially the first in vivo, one-time gene editing treatment for HAE.

**Acknowledgements** This study is funded by Intellia Therapeutics.

**References**

1. Longhurst HJ, Lindsay K, Petersen RS, et al. CRISPR-Cas9 In Vivo Gene Editing of KLKB1 for Hereditary Angioedema. N Engl J Med. 2024;390(5):432-441.
2. Cohn DM, Gurugama P, Magerl M, et al. CRISPR-Based Therapy for Hereditary Angioedema. N Engl J Med. 2025;392(5):458-467.

## P-13 Therapeutic trends in long-term prophylaxis in adult HAE patients in Serbia 2020–2024

### Radovan Mijanović^1^, Sladjana Andrejević^1^, Dusan Markovic^2^

#### ^1^Clinic of Allergy and Immunology, University Clinical center of Serbia, Belgrade, Serbia; ^2^Center for Medical Biochemistry, University Clinical Center Nis, Nis, Serbia

***Allergy, Asthma & Clinical Immunology*** 2025, **21(Suppl 2)**:P-13

**Background:** New drugs had been dis-covered and registered for long-term prophylaxis (LTP) in patients with hereditary angioedema (HAE) type 1 & 2 in the last decade. Also, international guideline for the management of HAE has been updated in the last couple of years.

**Materials and methods:** Medical records of all registered adult HAE patients in Serbia were examined in 2020. and in 2024. for LTP usage.

**Results:** In 2020. in Serbia 86 HAE patients were registered. Among them 19 (22%) patients have no regulated health insurance or living abroad. 67 patients (78%) had approved treatment in Serbia among whom 7 were minors. 24 adult HAE patients were on LTP: 20 patients were on danazol, 2 patients were taking tranexamic acid and one patient was taking berotralstat and one was receiving Lanadelumab, respectively.

In 2024. 100 HAE patients were registered, among whom 87 were adult. 2 patients have no regulated health insurance (1 minor and 1 adult). Among registered adult patients 26 were on HAE LTP: 3 patients were on danazol, 5 patients were taking tranexamic acid, one patient was taking berotralstat and 17 patients were receiving Lanadelumab.

**Conclusions:** For four years 14 new HAE patients were diagnosed in Serbia. Therapeutic trends in LTP of HAE patients in Serbia revealed that majority of patients were treated according to the newest international guidelines.

## P-14 Garadacimab-mediated inhibition of activated factor XII does not increase risk of thrombosis in patients with HAE

### Emel Aygören-Pürsün^1^, Markus Magerl^2,3,4^, Constance Katelaris^5^, F. Ida Hsu^6^, Harsha Shetty^7^, Iris Jacobs^7^, John-Philip Lawo^8^, Avner Reshef^9^

#### ^1^University Hospital Frankfurt, Department for Children and Adolescents, Goethe University Frankfurt, Frankfurt, Germany; ^2^Institute of Allergology, Charité–Universitätsmedizin Berlin, Berlin, Germany; ^3^Freie Universität Berlin and Humboldt-Universität zu Berlin, Berlin, Germany; ^4^Fraunhofer Institute for Translational Medicine and Pharmacology (ITMP), Immunology and Allergology, Berlin, Germany; ^5^Campbelltown Hospital, Sydney, New South Wales, Australia; ^6^Section of Rheumatology, Allergy & Immunology, Department of Medicine, Yale University School of Medicine, New Haven, CT, USA; ^7^CSL Behring, King of Prussia, PA, USA; ^8^CSL Innovation GmbH, Marburg, Germany; ^9^Allergy, Immunology and Angioedema Center, Barzilai University Hospital, Ashkelon, Israel

***Allergy, Asthma & Clinical Immunology*** 2025, **21(Suppl 2)**:P-14

**Background:** Hereditary angioedema (HAE) is hypothesised to be associated with elevated risk of venous thromboembolism (VTE), as highlighted by a recent registry study that showed the risk of VTE was doubled in patients versus their relatives without HAE. Activated factor XII (FXIIa) is the principal initiator of the kallikrein–kinin system that drives bradykinin release in HAE and the intrinsic coagulation pathway and plays a key role in the fibrinolytic pathway. Garadacimab (fully human anti-FXIIa antibody) has been evaluated for long-term prophylaxis of HAE in a comprehensive clinical programme. Garadacimab 200 mg subcutaneous once monthly provided early and durable protection against HAE attacks with a favourable safety profile. Here, we examine the occurrence of thromboembolic events (TEEs) monitored in patients with HAE during the garadacimab clinical programme.

**Materials and methods:** Garadacimab was evaluated in Phase 2 (n = 32), pivotal Phase 3 (n = 64), and ongoing Phase 3 open-label extension (OLE) (n = 161) studies. Patients received garadacimab 75 mg, 200 mg, 400 mg, or 600 mg subcutaneously in these studies, and were closely monitored for TEEs. A signal detection tool was used to identify additional potential TEEs per standardised Medical Dictionary for Regulatory Activities query (SMQ) methodology (SMQ: ‘[embolic and thrombotic events]’; data cutoff: February 2023). The incidence of TEEs was evaluated post hoc by means of an integrated analysis across the studies. Serum levels of D-dimer and prothrombin fragments (PF1 + 2) were also assessed to further detect the potential occurrence of TEEs.

**Results:** No garadacimab-related TEEs were reported during the clinical programme (n = 172, mean exposure 2.5 years). SMQ analysis yielded one ‘hit’ of venous thrombophlebitis in the Phase 3 OLE, which was unrelated to garadacimab and was medically confirmed to be a localised event, therefore not classed as a TEE. D-dimer and PF1 + 2 values were similar in patients treated with garadacimab versus values in patients treated with placebo in the pivotal Phase 3 study, remaining either unchanged versus pre-treatment or improving towards normal levels in both the pivotal Phase 3 and Phase 3 OLE studies.

**Conclusions:** No treatment-related TEEs were observed in the garadacimab clinical programme. Serum biomarker patterns did not indicate an increased risk of thrombosis and were observed to normalise during garadacimab treatment. This suggests that inhibition of FXIIa with garadacimab does not increase the potential risk of thrombosis in patients with HAE.

## P-15 Rationale and design of the Garadacimab REAl-world treatment outcomes of effec-tiveness, safety and quality-of-life in patients with hereditary angioedema (GREAT) study

### Andrea Zanichelli^1,2^, Markus Magerl^3,4,5^, Laurence Bouillet^6,7,8^, Stephen D. Betschel^9^, Sinisa Savic^10^, Chiara Nenci^11^, Vai Katkade^12^, Marc A. Riedl^13^

#### ^1^Operative Unit of Medicine, Angioedema Center, IRCCS Policlinico San Donato, Milan, Italy; ^2^Department of Biomedical Sciences for Health, University of Milan, Milan, Italy; ^3^Institute of Allergology, Charité–Universitätsmedizin Berlin, Berlin, Germany; ^4^Freie Universität Berlin and Humboldt-Universität zu Berlin, Berlin, Germany; ^5^Fraunhofer Institute for Translational Medicine and Pharmacology (ITMP), Immunology and Allergology, Berlin, Germany; ^6^Translational Research in Autoimmunity and Inflammation Group, TIMC, Grenoble Alpes University, Grenoble, France; ^7^French National Reference Center for Angioedema (CREAK), Grenoble, France; ^8^Internal Medicine Department, Grenoble Alpes University Hospital, Grenoble, France; ^9^Division of Clinical Immunology and Allergy, Department of Medicine, University of Toronto, Toronto, ON, Canada; ^10^Leeds Teaching Hospitals NHS Trust, Leeds, UK; ^11^CSL Behring AG, Bern, Switzerland; ^12^CSL Behring, King of Prussia, PA, USA; ^13^University of California San Diego, San Diego, CA, USA

***Allergy, Asthma & Clinical Immunology*** 2025, **21(Suppl 2)**:P-15

**Background:** Garadacimab (anti-activated factor XII antibody) has demonstrated early and durable efficacy with a favourable long-term safety profile in the Phase 2, pivotal Phase 3 (VANGUARD) and ongoing Phase 3 open-label extension studies in patients with hereditary angioedema (HAE). In 2025, garadacimab was approved for use as once-monthly long-term prophylaxis (LTP) in patients with HAE in Europe, the UK, Australia and Japan. Real-world data provide insights from routine clinical practice and can be used to inform treatment decisions in HAE. The GREAT study will investigate long-term effectiveness, safety, health-related quality of life (HRQoL) and healthcare resource utilisation (HCRU) outcomes in patients with HAE receiving garadacimab LTP in the real-world setting.

**Materials and methods:** This prospective, noninterventional, observational cohort study will enrol patients aged ≥ 12 years with physician- and laboratory-confirmed HAE newly initiating on-label garadacimab (200 mg subcutaneous once monthly) from ~ 30 centres in Europe, the UK, North America and the Asia–Pacific region (target enrolment: 200 patients). Participants will be observed on treatment for a target duration of 24 months with a 30-day follow-up period. The GREAT study will evaluate the characteristics of the enrolled population, HAE attack rate (on treatment with garadacimab vs pre-enrolment baseline), time to occurrence of first HAE attack, the proportion of patients achieving attack-free status and attack-free duration. It will assess safety, HRQoL, productivity, treatment patterns, HCRU and the economic impact of garadacimab LTP, treatment adherence and satisfaction, and patient preference. Retrospective data on HAE attacks and treatments, and on HCRU will be collected from 12 months before garadacimab initiation.

**Results:** The study will begin to enrol patients in mid-2025 and is estimated to complete in 2029.

**Conclusions:** The GREAT study will provide the first comprehensive real-world evidence on the use of garadacimab as LTP against HAE.

## P-16 Avoiding misdiagnosis in hereditary angioedema: A case report

### Irina E. Guryanova^1,*^, Aliaksandr V. Liubushkin^1^, Ekaterina A. Polyakova^1^, Andrej P. Salivonchik^2^, Vladislav R. Vertelko^1^, Mikhail V. Belevtsev^1^, Anzhelika V. Solntsava^1^

#### ^1^Belarussian Research Center for Pediatric Oncology, Hematology and Immunology, Minsk, Belarus; ^2^Centre for Radiation Medicine and Human Ecology, Gomel, Belarus

##### **Correspondence:** Irina E. Guryanova (guryanovairina1985@gmail.com)

***Allergy, Asthma & Clinical Immunology*** 2025, **21(Suppl 2)**:P-16

**Background:** C1-inhibitor deficiency can be either hereditary (hereditary angioedema due to C1-inhibitor deficiency, C1-INH-HAE) or acquired (Acquired Angioedema due to C1-inhibitor deficiency, C1-INH-AAE). Both conditions are characterized by recurrent non-urticarial angioedema, with reductions in C1-inhibitor levels and complement C4. C1-INH-HAE is caused by variants in the SERPING1 gene, leading to C1-inhibitor deficiency from birth, with disease manifestation typically occurring during adolescence, but possible at any age. In contrast, C1-INH-AAE usually manifests after the age of 40 and is most commonly associated with underlying conditions such as lymphoproliferative disorders, autoimmune diseases, or solid tumors, but in some cases, occurs in the absence of a known underlying disorder.

**Materials and Methods:** This study included a patient (born in 1969) with recurrent non-urticarial angioedema, first presenting at the age of 29. No family history of similar symptoms was reported. The patient's daughter (31 years old) has not experienced any swelling. The following analyses were performed: C4 (Abbott, USA) and C1-inhibitor levels (Technoclone, Austria); next-generation sequencing (NGS) using the PID pro panel (452 genes, 4bases, Switzerland), including SERPING1; copy number variation (CNV) analysis using 4eVAR1.0 software (4bases, Switzerland); and multiplex ligation-dependent probe amplification (MLPA) of SERPING1 and F12 genes (MRC Holland, Netherlands). Written informed consent was obtained before study participation.

**Results:** The patient's medical history does not include any diseases or conditions typically associated with C1-INH-AAE. In the past 12 months, the patient experienced 15 episodes of swelling, which occurred in the limbs (1), lips (4), larynx (1), and abdomen (9). Complement C4 levels were significantly reduced (< 0.029 g/L; reference: 0.2–0.5 g/L), as was the level of C1-inhibitor (0.03 g/L; reference: 0.21–0.39 g/L). Sequencing analysis of the SERPING1 gene did not reveal any single-nucleotide variants or small deletions/insertions that could explain the patient’s symptoms. CNV analysis revealed a heterozygous deletion of 5–6 exons in the SERPING1 gene (chr11:57373484–57374020), classified as likely pathogenic according to Franklin. The deletion was confirmed by MLPA. In the DNA sample of the patient’s daughter, CNV and MLPA analyses did not detect this deletion.

**Conclusion:** The sequencing analysis of exons and their splice site regions does not allow for the detection of large heterozygous deletions or insertions. By using both qualitative and quantitative genetic testing methods, clinicians can avoid misdiagnosis, particularly in cases with late disease onset or limited familial information, and ensure appropriate management and treatment.

## P-17 Acquired angioedema due to C1-inhibitor deficiency: Patient experience and assessment of patient-reported outcome measures

### Andrea Zanichelli^1,2^, Danny M. Cohn^3^, Marc A. Riedl^4^, Sarah Clifford^5^, Beverly Romero^5^, Kelsie Brewer^5^, Swaha Pattanaik^5^, Maggie Chen^6^, Joan Mendivil^7^

#### ^1^Universita degli Studi di Milano, Milan, Italy; ^2^IRCCS, UO Medicina, Milan, Italy; ^3^Amsterdam UMC, University of Amsterdam, Amsterdam, The Netherlands; ^4^University of California San Diego, La Jolla, CA, USA; ^5^Sprout Health Solutions, Los Angeles, CA, USA; ^6^Pharvaris Inc, Lexington, MA, USA; ^7^Pharvaris GmbH, Zug, Switzerland

***Allergy, Asthma & Clinical Immunology*** 2025, **21(Suppl 2)**:P-17

**Rationale:** Acquired angioedema due to C1-inhibitor deficiency (AAE-C1-INH) is a rare disease mediated by bradykinin and characterized by painful swelling attacks. There are currently no approved prophylactic or on-demand treatments and no patient-reported outcome (PRO) measures validated for use in AAE-C1-INH. This study aimed to (1) create a conceptual model of AAE-C1-INH to describe patients’ experiences of symptoms and impact of AAE-C1-INH and (2) explore and confirm the relevance of adapting PRO measures validated for hereditary angioedema, another bradykinin-mediated disease with similar manifestations, for AAE-C1-INH.

**Methods:** This cross-sectional, remote, qualitative study included adults in the United States with AAE-C1-INH and no prior or concomitant diagnosis of other angioedema. A semi-structured interview guide with open-ended questions elicited patients’ descriptions of AAE-C1-INH manifestations and impact on their daily lives. Cognitive interviews assessed perceptions of clarity, comprehension, and meaningful levels of change of specific PRO measures, including Patient Global Impression of Change (PGI-C) and Severity (PGI-S) and two health-related quality-of-life (HRQoL) measures: Patient Global Assessment of Change (PGA-C) and Status (PGA-S).

**Results:** Of eight individuals interviewed (seven female; all white), all had ≥ 1 attack in the previous six months and six had ≥ 1 attack in the previous 12 weeks. Participants reported traumatic medical emergencies, misdiagnoses, and evaluations by a range of healthcare providers. Participants discussed ten unique attack areas, with abdomen (n = 7), face (n = 6), and foot/hand (n = 5 each) being most common. Daily life impact was common, particularly social/family and treatment-related (n = 7 each). Evaluating PGI-C, 8/8 participants correctly interpreted PGI-C to assess attack symptoms at a given time post-treatment vs at time of treatment for an AAE-C1-INH attack. 4/8 considered “a little better” meaningful up to 2 h post-treatment and 6/6 considered “better” meaningful up to 4 h. Evaluating PGI-S, 3/7 correctly interpreted PGI-S to describe attack symptom severity and 4/7 considered ≥ 1 level of change 4 h post-treatment meaningful. Evaluating PGA-C, 5/6 correctly interpreted PGA-C to describe overall change in HRQoL since starting medication and 5/6 considered “a little better” 12 weeks post-treatment would be meaningful. Evaluating PGA-S, 6/6 correctly interpreted PGA-S to describe how AAE-C1-INH impacts their HRQoL.

**Conclusions:** This study highlights the impact of AAE-C1-INH on patients’ daily lives and overall well-being. Most patients considered the patient global impression and assessment items easy to interpret and relevant, which may inform a clinical outcome assessment strategy for clinical trials in AAE-C1-INH.

## P-19 Long-term prophylactic treatment with oral deucrictibant improves disease control and health-related quality of life in participants with hereditary angioedema: CHAPTER-1 open-label extension study

### Markus Magerl^1,2^, John Anderson^3^, Francesco Arcoleo^4^, Emel Aygören-Pürsün^5^, Mauro Cancian^6^, Hugo Chapdelaine^7^, Niall Conlon^8^, Efrem Eren^9^, Mark Gompels^10^, Sofia Grigoriadou^11^, Maria D. Guarino^12^, Padmalal Gurugama^13^, Sorena Kiani-Alikhan^14^, Tamar Kinaciyan^15^, Michael E. Manning^16^, Marcin Stobiecki^17^, Michael D. Tarzi^18^, Anna Valerieva^19^, H. James Wedner^20^, William H. Yang^21^, Andrea Zanichelli^22.23^, Rafael Crabbé^24^, Susan Mulders^25^, Jonathan Levy^26^, Ulrich Freudensprung^27^, Umar Katbeh^27^, Jochen Knolle^28^, Anne Lesage^29^, Peng Lu^26^, Marc A. Riedl^30^

#### ^1^Charité – Universitätsmedizin Berlin, Berlin, Germany; ^2^Fraunhofer Institute for Translational Medicine and Pharmacology ITMP, Berlin, Germany; ^3^AllerVie Health, Birmingham, AL, USA; ^4^UOC di Patologia Clinica e Immunologia, Palermo, Italy; ^5^Goethe University Frankfurt, Frankfurt, Germany; ^6^University Hospital of Padua, Padua, Italy; ^7^Université de Montréal, Montréal, QC, Canada; ^8^St. James's Hospital and Trinity College, Dublin, Ireland; ^9^University Hospital Southampton NHS Foundation Trust, Southampton, UK; ^10^North Bristol NHS Trust, Bristol, UK; ^11^Barts Health NHS Trust, London, UK; ^12^Ospedale di Civitanova Marche, Civitanova Marche, Italy; ^13^Cambridge University Hospitals NHS Foundation Trust, Cambridge, UK; ^14^Royal Free London NHS Foundation Trust, London, UK; ^15^Medical University of Vienna, Vienna, Austria; ^16^Allergy, Asthma and Immunology Associates, Ltd., Scottsdale, AZ, USA; ^17^Jagiellonian University Medical College, Krakow, Poland; ^18^University Hospitals Sussex NHS Foundation Trust, Brighton, UK; ^19^Medical University of Sofia, Sofia, Bulgaria; ^20^Washington University School of Medicine, St. Louis, MO, USA; ^21^Ottawa Allergy Research Corporation, University of Ottawa, Ottawa, ON, Canada; ^22^Universita degli Studi di Milano, Milan, Italy; ^23^I.R.C.C.S., UO Medicina, Milan, Italy; ^24^RC Consultancy, Bassins, Switzerland; ^25^Mulders Clinical Consulting, Groesbeek, The Netherlands; ^26^Pharvaris Inc., Lexington, MA, USA; ^27^Pharvaris GmbH, Zug, Switzerland; ^28^JCK Consult, Frankfurt, Germany; ^29^GrayMatters Consulting, Schilde, Belgium; ^30^University of California San Diego, La Jolla, CA, USA

***Allergy, Asthma & Clinical Immunology*** 2025, **21(Suppl 2)**:P-19

**Rationale**: International hereditary angio-edema (HAE) guidelines recommend that the goals of treatment are to achieve total disease control and to normalize patients' lives. Deucrictibant is a selective, orally administered bradykinin B2 receptor antagonist under development for prophylactic and on-demand treatment of HAE attacks. CHAPTER-1 (NCT05047185) is a two-part, Phase 2 study evaluating the efficacy and safety of deucrictibant for long-term prophylaxis of HAE attacks. Part 1 is complete and an open-label extension (OLE; part 2) is ongoing. In the double-blind, placebo-controlled part 1, participants reported improved disease control and health-related quality of life (HRQoL) vs placebo from as early as week 4 and through week 12. Participants also reported high levels of satisfaction with deucrictibant treatment.

**Methods:** Thirty participants completed part 1, during which they received deucrictibant 20 mg/day (n = 11), 40 mg/day (n = 10), or placebo (n = 9) for 12 weeks. All 30 participants continued into the ongoing OLE and received treatment with deucrictibant 40 mg/day. Patient-reported outcomes (PROs) evaluated disease control, HRQoL, and satisfaction with treatment. Assessments included Angioedema Control Test (AECT), Patient Global Assessment of Change (PGA-Change), Angioedema QoL Questionnaire (AE-QoL), and Treatment Satisfaction Questionnaire for Medication (TSQM-II). Here, PRO outcomes from the ongoing CHAPTER-1 OLE are reported.

**Results:** This data snapshot (cutoff: 10 June 2024) included 14 participants who had received deucrictibant treatment up to at least week 62 at the time of data cutoff. As measured using AECT, 100% (14/14) of participants reported well-controlled HAE at week 62. All participants also reported an improvement in PGA-Change (indicating improved HRQoL) at week 62 vs baseline, with 93% (13/14) feeling “much better” and 7% (1/14) feeling “a little better”. Furthermore, the mean AE-QoL total score improved by 28.2 points from study baseline to week 62. The AE-QoL domains that showed the greatest improvement with deucrictibant treatment at week 62 vs study baseline were “fear/shame” (32.1-point improvement) and “functioning” (47.3-point improvement). Participants also reported high treatment satisfaction as measured using TSQM.

**Conclusions:** PRO data from the ongoing CHAPTER-1 OLE study provide evidence on the maintenance of improvements in disease control and HRQoL, as well as on the high satisfaction with long-term deucrictibant treatment in participants with HAE.

## P-20 Sustained therapeutic exposure with once-daily oral deucricti-bant xr tablet for prophylaxis of hereditary angioedema attacks: Results of a pharmacokinetics study in healthy volunteers

### Zhi-Yi Zhang^1^, Peng Lu^1^, Kees Groen^2^, Rafael Crabbé^3^

#### ^1^Pharvaris Inc., Lexington, MA, USA; ^2^DGr Pharma BV, Oudenbosch, The Netherlands; ^3^RC Consultancy, Bassins, Switzerland

***Allergy, Asthma & Clinical Immunology*** 2025, **21(Suppl 2)**:P-20

**Rationale**: Deucrictibant is a selective, orally administered bradykinin B2 receptor antagonist under development for prophylactic and on-demand treatment of hereditary angioedema (HAE) attacks. Deucrictibant was evaluated in Phase 2 clinical trials for prophylactic (CHAPTER-1, NCT05047185) and on-demand (RAPIDe-1, NCT04618211) treatment of HAE attacks. In both trials, deucrictibant was efficacious and generally well tolerated. In the prophylaxis CHAPTER-1 trial, deucrictibant was administered as immediate-release (IR) capsule formulation, dosed twice daily, as a proof-of-concept for the once-daily deucrictibant extended-release (XR) tablet, which is the intended formulation of deucrictibant for prophylactic HAE treatment.

**Methods:** PHA022121-C020 was a phase 1, open-label, randomized, 2-period, crossover study in healthy volunteers. The primary objective was the characterization of single-dose pharmacokinetics of deucrictibant XR tablet (40 mg) and deucrictibant IR capsule (2 × 20 mg). Secondary objectives included assessment of relative bioavailability, safety, and tolerability of both formulations. Participants received study drug as a single dose under fasting conditions.

**Results:** This analysis included 14 participants with evaluable data. Mean (SD) maximum plasma concentration (Cmax) was 87 (26) and 515 (154) ng/mL for deucrictibant XR tablet and deucrictibant IR capsule, respectively. Mean (SD) plasma concentration at 24 h post-dose (C24h) was 52 (30) and 8 (7) ng/mL. Deucrictibant XR resulted in a sustained level in circulation exceeding the therapeutic threshold from 1.5 to at least 24 h post-dose. Mean C24h was approximately fourfold higher than the effective concentration that leads to 85% maximal response (EC85; 13.8 ng/mL); mean C24h was higher than EC85 in 12/14 participants and higher than EC50 in all participants. From the time of dosing to the time of last measurable concentration, the area under the curve (AUClast) was 1472 and 1652 ng•h/mL for deucrictibant XR tablet and deucrictibant IR capsule, respectively. Geometric least square mean ratios of AUClast showed that relative bioavailability and overall exposure of the two formulations were comparable. Deucrictibant XR was well tolerated and no adverse events were reported in preliminary data.

**Conclusions:** A single oral deucrictibant XR tablet resulted in sustained exposure for at least 24 h which supports once-daily dosing. Results showed an approximate fivefold lower Cmax and sixfold higher C24h with deucrictibant XR tablet vs deucrictibant IR capsule. Preliminary data provide evidence that deucrictibant XR tablet was well tolerated, with no adverse events. This pharmacokinetic profile supports further investigation of the efficacy and safety of deucrictibant XR tablet for once-daily prophylaxis of HAE attacks in Phase 3 trials.

## P-21 Optical genome mapping for the identification of complex structural variants in hereditary angioedema due to C1-inhibitor deficiency

### Roger Colobran^1,2,3,4^, Mar Guilarte^5,6^, Johana Gil-Serrano^5,6^, Aina Aguiló-Cucurull^1,2^, Kornelia Neveling^7^, Alexander Hoischen^7,8,9^, Laura Batlle-Masó^1^

#### ^1^Translational Immunology Research Group, Vall d’Hebron Research Institute (VHIR), Barcelona, Catalonia, Spain; ^2^Immunology Division, Vall d’Hebron University Hospital (HUVH), Barcelona, Catalonia, Spain; ^3^Department of Clinical and Molecular Genetics, Vall d'Hebron University Hospital (HUVH), Barcelona, Catalonia, Spain; ^4^Department of Cell Biology, Physiology and Immunology, Autonomous University of Barcelona (UAB), Bellaterra, Catalonia, Spain; ^5^Allergy Department, National Reference Hereditary Angioedema Center (CSUR), Hospital Universitari Vall d'Hebron, Barcelona, Catalonia, Spain; ^6^Allergy Research Unit, Vall d'Hebron Research Institute (VHIR), Barcelona, Catalonia, Spain; ^7^Radboud University Medical Center, Department of Human Genetics, Nijmegen, The Netherlands; ^8^Radboud University Medical Center, Department of Internal Medicine and Radboud Institute for Molecular Life Sciences, Nijmegen, The Netherlands; ^9^Radboud Expertise Center for Immunodeficiency and Autoinflammation and Radboud Center for Infectious Disease, Radboud University Medical Center, Nijmegen, The Netherlands

***Allergy, Asthma & Clinical Immunology*** 2025, **21(Suppl 2)**:P-21

**Background:** Hereditary angioedema (HAE) due to C1-inhibitor deficiency (HAE-C1-INH) is the most common form of HAE. It is caused by heterozygous pathogenic variants in the SERPING1 gene. A distinctive feature of SERPING1 is the relatively high proportion of large gene rearrangements that are usually identified by multiplex ligation-dependent probe amplification (MLPA). A limitation of MLPA is that it typically targets only the coding regions (exons) of the gene. Optical genome mapping (OGM) is a high-resolution technique that uses fluorescently labeled long DNA molecules to detect structural variants (SV) with high precision. The aim of this study is to prove that OGM can effectively identify complex SV in patients with HAE-C1-INH.

**Materials and methods:** MLPA, OGM and long read whole genome sequencing (LR-WGS) were used to identify SV in two families with HAE-C1-INH. For OGM and LR-WGS, ultra-high molecular weight (UHMW) DNA was obtained from patients’ blood.

**Results:** First, we aimed to verify that OGM was able to detect SV affecting the coding region of SERPING1. We obtained UHMW DNA from two related patients (mother and daughter) who had a previously identified large heterozygous deletion encompassing the two first exons of SERPING1. We then performed OGM, successfully identifying this deletion and confirming that OGM can detect SV affecting the coding region in HAE-C1-INH.

Next, we tested a family with three HAE-C1-INH patients in whom no pathogenic variant had been identified in the SERPING1 gene after Sanger sequencing and MLPA. We obtained UHMW DNA from one patient and we performed OGM. We identified a 3.8 kb insertion located in the region between intron 6 and the 3’UTR of SERPING1. Since OGM is not a sequencing technique, we could not determine the sequence and the exact insertion site of the variant. To resolve this, we performed LR-WGS. Results showed that the insertion was a SINE-VNTR-Alu (SVA) transposable element. SVA are the evolutionarily youngest retrotransposon family in the human genome and they are still active mobile elements. By analyzing the cDNA we found that the SVA insertion alters mRNA splicing, leading to SERPING1 haploinsufficiency and causing HAE-C1-INH in this family.

**Conclusions:** OGM is a reliable and robust technique for identifying SVs affecting both coding and non-coding regions in HAE-C1-INH. Using OGM, we report the first case of HAE-C1-INH caused by the insertion of an SVA mobile element into a non-coding region of SERPING1.

**Acknowledgements** This work was supported by Solve-RD and ERN-RITA.

## P-22 Somatic variants and mosaicism in hereditary angioedema due to C1-inhibitor deficiency: Implications for genetic counselling

### Roger Colobran^1,2,3,4^, Laura Batlle-Masó^1^, Janire Perurena-Prieto^1,2^, Laura Viñas-Giménez^1,2^, Aina Aguiló-Cucurull^1,2^, Johana Gil-Serrano^5,6^, Mar Guilarte^5,6^

#### ^1^Translational Immunology Research Group, Vall d’Hebron Research Institute (VHIR), Barcelona, Catalonia, Spain; ^2^Immunology Division, Vall d’Hebron University Hospital (HUVH), Barcelona, Catalonia, Spain; ^3^Department of Clinical and Molecular Genetics, Vall d'Hebron University Hospital (HUVH), Barcelona, Catalonia, Spain; ^4^Department of Cell Biology, Physiology and Immunology, Autonomous University of Barcelona (UAB), Bellaterra, Catalonia, Spain; ^5^Allergy Department, National Reference Hereditary Angioedema Center (CSUR), Hospital Universitari Vall d'Hebron, Barcelona, Catalonia, Spain; ^6^Allergy Research Unit, Vall d'Hebron Research Institute (VHIR), Barcelona, Catalonia, Spain

***Allergy, Asthma & Clinical Immunology*** 2025, **21(Suppl 2)**:P-22

**Background:** Hereditary angioedema (HAE) due to C1-inhibitor deficiency (HAE-C1-INH) is a rare genetic disease caused by pathogenic variants in the SERPING1 gene, typically in an autosomal dominant inheritance pattern. More than 700 different pathogenic variants have been reported in patients with HAE-C1-INH. Unlike other genetic forms of HAE, up to 15% of HAE-C1-INH cases are sporadic, resulting from de novo mutations. However, some of these apparently sporadic cases may result from somaƟc variants present in one of the progenitors, leading to mosaicism.

**Materials and methods:** Sanger sequencing, RNA isolation and cDNA analysis, next-generation sequencing (NGS)-based deep amplicon sequencing (DAS), systematic literature review.

**Results:** In this study, we conducted a systematic literature review on mosaicism in HAE-C1-INH and we present a novel case, outlining strategies to address this phenomenon using cutting-edge molecular techniques. To date, we found only three published cases of mosaicism in HAE-C1-INH. Two of them corresponded to gonadal mosaicism (i.e. the somatic variant is only present in gonads) and the other to gonosomal mosaicism (i.e. the somatic variant is present in gonads and other tissues). In all these cases, mosaicism was suspected only after a second HAE-C1-INH-affected child. We report the case of a patient with apparently sporadic HAE-C1-INH, presenting with compatible clinical symptoms and markedly reduced C1-INH function. Both parents showed normal C4 levels and normal C1-INH function. Genetic analysis of the patient revealed a novel splice site mutation in SERPING1 (c.890-1G > C), confirmed as pathogenic through cDNA analysis. Surprisingly, despite the mother’s normal C1-INH function, she was a mutation carrier. The inverted profile of the Sanger sequencing peaks, compared to the patient’s, strongly suggested that the variant was somatic, leading to a gonosomal mosaicism in the mother. This was further confirmed and quantified across DNA from different tissues using highdepth (> 5,000 reads) NGS-DAS, revealing a variant frequency ranging from 17–23%. Due to the inability of testing the degree of mosaicism in the gonads, a precise assessment of recurrence risk was difficult. Adopting a conservative approach in the genetic counselling, we assumed the same risk as in germline SERPING1 mutations (50%) by default.

**Conclusions:** In this study, we present the first case of gonosomal mosaicism in a family with a single child affected by HAE-C1-INH, despite both parents being unaffected. Our findings emphasize the importance of parental genetic testing in all patients, regardless of their clinical presentation, and highlight the critical implications of gonosomal mosaicism for genetic counseling.

## P-23 First Bulgarian family with hereditary angioedema due to plasminogen gene mutation: Clinical insights and treatment approaches

### Anna Valerieva^1^, Alex Fam^1^, Ferhat Maksudov^1^, Elena Petkova^1^, Krasimira Baynova^2^, Stefan Cimbollek^2^, Teresa De Aramburu^2^, José Lucena^3^, Maria Staevska^1^

#### ^1^Department of Allergology, Medical University of Sofia, University Hospital “Alexandrovska”, Sofia, Bulgaria; ^2^Allergy Department, Spanish National Center for Angioedema, Virgen del Rocío University Hospital, Seville, Spain; ^3^Immunology Department, Spanish National Center for Angioedema, Virgen del Rocío University Hospital, Seville, Spain

***Allergy, Asthma & Clinical Immunology*** 2025, **21(Suppl 2)**:P-23

**Background**: Hereditary angioedema (HAE) is a rare genetic disorder marked by episodic cutaneous or submucosal edema, including skin swellings, abdominal attacks, and life-threatening upper airway obstruction. HAE with plasminogen gene mutation (HAE-PLG) is identified by the pathogenic variant c.988A > G in exon 9, resulting in the missense mutation p.Lys330Glu (K330E) within the kringle 3 domain of the PLG protein, with autosomal-dominant inheritance. To date, 146 patients from 33 families have been reported globally.

**Patients and Methods**: We examined the clinical manifestations of a family initially diagnosed with HAE with normal C1-Inhibitor (HAE-nC1-INH) through clinical data, biochemical analysis for C1-INH and C4. For each patient DNA from oral mucosa was studied using the next-generation sequencing( NGS) platform targeting 45 genes from kallikrein kinin system including PLG.[1].

**Case Presentation**: Gene sequencing revealed the first Bulgarian family with HAE-PLG: the c.988A > G variant in exon 9 p.Lys330Glu (K330E). The proband, a 76-year-old male, experienced recurrent angioedema primarily affecting the face (lips, tongue, pharynx), with symptom onset at age 50. He reported abdominal attacks and multiple misdiagnoses over the years, with two severe episodes occurring during ACE inhibitor treatment. Acute attacks were effectively managed with icatibant.

His daughter, recently diagnosed, had symptom onset at age 48 with a single angioedema episode, followed by an asymptomatic period until age 52. No aggravating factors were identified, including pregnancy. Angioedema was primarily localized to the lips and tongue, typically resolving within 48 h despite conventional therapies. Upon HAE-nC1-INH diagnosis, on-demand icatibant treatment was initiated with good response. Severe episodes required repeated redosing, and before gene sequencing results, omalizumab prophylaxis was initiated with uncertain effect.

No other family members reported recurrent angioedema. Family screening for the granddaughter of the proband is pending.

**Discussion**: This report presents the first Bulgarian family with HAE-PLG, detailing the clinical course, treatment challenges, and explored modalities in the context of limited diagnostic tools to identify HAE-nC1-INH and rare genetic variants linked to HAE. Genetic study is crucial for the cases of HAE-C1-INH in order to understand better its pathogenesis and perform screening of asymptomatic family members.

[1] The genetic study was carried out in accordance with the ethical committee of Virgen del Rocío University Hospital. Written informed consent was signed by all subjects.

## P-25 Time to end of progression of hereditary angioedema attacks treated with sebetralstat

### William R. Lumry^1^, John Anderson^2^, Jonathan A. Bernstein^3^, Mauro Cancian^4^, Danny M. Cohn^5^, Henriette Farkas^6^, Henry Li^7^, James Hao^8^, Michael Smith8, Paolo Bajcic^8^, Paul Audhya^8^, Markus Magerl^9,10^

#### ^1^AARA Research Center, Dallas, TX, USA; ^2^Clinical Research Center of Alabama, an affiliate of AllerVie Health, Birmingham, AL, USA; ^3^University of Cincinnati College of Medicine and Bernstein Clinical Research Center, Cincinnati, OH, USA; ^4^Departmental Allergy Division, Department of Systems Medicine, University of Padua, Padua, Italy; ^5^Department of Vascular Medicine, Amsterdam Cardiovascular Sciences, Amsterdam University Medical Center, University of Amsterdam, Amsterdam, The Netherlands; ^6^Hungarian Angioedema Center of Reference and Excellence, Department of Internal Medicine and Haematology, Semmelweis University, Budapest, Hungary; ^7^Institute for Asthma and Allergy, Wheaton, MD, USA; ^8^KalVista Pharmaceuticals, Salisbury, United Kingdom, and Cambridge, MA, USA; ^9^Institute of Allergology, Charité-Universitätsmedizin Berlin, corporate member of Freie Universitätsmedizin Berlin and Humboldt-Universität zu Berlin, Berlin, Germany; ^10^Fraunhofer Institute for Translational Medicine and Pharmacology ITMP, Immunology and Allergology, Berlin, Germany

***Allergy, Asthma & Clinical Immunology*** 2025, **21(Suppl 2)**:P-25

**Background:** On-demand treatments for hereditary angioedema (HAE) attacks aim to interdict the plasma kallikrein-kinin cascade and halt the progression of swelling. Stopping progression as early as possible after attack onset is important to minimize severity and reduce morbidity. Administration of sebetralstat, an investigational oral plasma kallikrein inhibitor, has previously been shown to achieve rapid plasma exposure and near-complete inhibition of plasma kallikrein within 15 min in patients with HAE. This post hoc analysis examines the time to end of attack progression following treatment with sebetralstat in KONFIDENT-S (NCT05505916) and KONFIDENT (NCT05259917).

**Materials and Methods:** End of progression was defined as the time at which the worst attack severity was recorded using the 5-point Patient Global Impression of Severity (PGI-S) scale. PGI-S ratings (ranging from “very severe” to “none”) were recorded by participants every 0.5 h during the first 4 h after taking sebetralstat, then every hour from 5 to 12 h. This analysis includes attacks ranging from “None” to “very severe” at the time of treatment in the ongoing KONFIDENT-S open-label extension study (Sept 2024 data cutoff) and Phase 3 KONFIDENT trial. Attacks treated with conventional therapy were right-censored at the time of use. Attacks with no post-baseline assessment were excluded.

**Results:** Analysis included 1591 attacks (36% mild, 42% moderate, 17% severe, 4% very severe) treated with 600 mg sebetralstat from KONFIDENT-S and 84 attacks (43% mild, 39% moderate, 14% severe, 2% very severe) treated with 300 mg sebetralstat and 88 attacks (46% mild, 34% moderate, 18% severe, 2% very severe) treated with 600 mg sebetralstat from KONFIDENT. The median (interquartile range) time to end of progression within 4 h of administration was 19.8 min (16.2–42.6) for attacks treated with 600 mg sebetralstat in KONFIDENT-S, which was similar to 19.8 min (16.8–97.2) and 19.2 min (16.8–46.2) for attacks treated with 300 mg and 600 mg sebetralstat in KONFIDENT. In KONFIDENT-S, 90.3% of attacks treated with sebetralstat reached end of progression within 4 h of administration. In KONFIDENT, 82.1% of attacks treated with 300 mg sebetralstat and 89.81% treated with 600 mg sebetralstat, reached end of progression within 4 h.

**Conclusions:** In this post hoc analysis, treatment with sebetralstat 600 mg ended progression of HAE attacks early, with a median of 19.8 min in KONFIDENT-S, which was consistent with results from the KONFIDENT trial (medians of 19.8 and 19.2 min for 300 mg and 600 mg sebetralstat, respectively). End of progression rapidly followed the expected time to near complete inhibition of plasma kallikrein based on prior pharmacodynamic studies.

## P-26 Clinical investigation of hereditary angioedema linked to coagulation factor XII mutations in a Southern Spanish cohort

### Teresa De Aramburu Mera, Krasimira Baynova, José Raúl García Lozano, Jose Manuel Lucena Soto, Stefan Cimbollek

#### Spanish National Center for Angioedema Allergy Department Virgen del Rocío University Hospital, Seville, Spain

***Allergy, Asthma & Clinical Immunology*** 2025, **21(Suppl 2)**:P-26

**Introduction:** Numerous alterations genetic in the Factor XII gene associated with HAE-FXII have been documented since its genetic characterization in 2006. Individuals afflicted with hereditary angioedema (HAE) attributable to variants in the Factor XII gene exhibit distinctive characteristics in comparison to other HAE endotypes as well as among members of the same lineage.

The objective of our investigation is to elucidate the demographic, clinical, and genetic attributes of patients diagnosed with HAE-FXII.

**Materials and methods:** We selected 12 index cases from diverse familial backgrounds diagnosed with HAE characterized by:

- Normal levels of C1-INH.
- Absence of pathogenic SERPING1 gene variants.
- Presence of pathogenic variants in the coagulation factor XII gene.

From these 12 index cases, 45 immediate family members were selected for comprehensive genetic and clinical analysis. We collected clinical information encompassing gender, date of birth, familial relationships, age of symptom onset, therapeutic requirements, diagnostic delays, and correlations with estrogen exposure.

**Results**: All index cases were female, with a mean age of symptom onset at 20 years. Eleven subjects demonstrated a significant association between the onset of attacks and estrogen exposure, and seven experiencing exacerbations during gestation. Each patient required emergency medical intervention at least once, and three utilized tranexamic acid (ATX) as a prophylactic measure.

Among the 45 family members analyzed, the c.983C > A variant was identified as heterozygous in 27 individuals, comprising 8 males and 19 females, with all male participants remaining asymptomatic. Within the female cohort, 12 exhibited symptoms while 7 were asymptomatic. In symptomatic group, they presented a mean age of symptom onset of 23 years. Seven women reported exacerbations of angioedema in association with estrogen exposure, and 6 noted worsening during pregnancy. Seven sought emergency medical care at least once, and one received prophylactic ATX.

A significant correlation exists between estrogen exposure and symptomatic presentation in females. This exposure has been associated with an earlier onset of symptoms (p = 0.016), as exposed females experience attacks at an average age of 20 years, in contrast to their unexposed counterparts at 25 years; predominantly, patients experienced peripheral episodes (87.5%), followed by abdominal (54%) and oropharyngeal (25%) episodes. The diagnostic delay was recorded at 16 years. All patients subjected to estrogen required rescue medication, in contrast to 33% of those who were not exposed (p = 0.001); C1-INH concentrate was administered in 57% of patients. Corticosteroids were the main intervention, although without efficacy.

**Conclusion:** HAE-FXII represents a rare disorder characterized by intricate multifactorial traits, significantly influenced by estrogen and a gender bias favoring female.

## P-27 Genetic segregation study in patients with hereditary angio-edema due to mutation in coagulation factor XII in a population of Southern Spain

### Teresa De Aramburu Mera, Krasimira Baynova, José Raúl García Lozano, Jose Manuel Lucena Soto, Stefan Cimbollek

#### Spanish National Center for Angioedema Allergy Department Virgen del Rocío University Hospital, Seville, Spain

***Allergy, Asthma & Clinical Immunology*** 2025, **21(Suppl 2)**:P-27

**Introduction:** Patients with hereditary angioedema due to pathogenic variants in the coagulation factor XII gene show clinical differences not only with other forms of hereditary angioedema, but also show differences between different families with HAE-FXII and even between members of the same family.

Our study aims to identify additional genes that may shed light on the phenotypic diversity observed in our cohort.

**Material and methods:** We selected 12 index cases from different families diagnosed with HAE who met specific criteria:

- Normal C1-INH.
- No pathogenic SERPING1 gene variants.
- Pathogenic variants present in the coagulation factor XII gene.

From these 12 index cases, 45 immediate family members were selected for genetic and clinical research.

The genetic analysis comprised:

- Initially, the 14 exons of the F12 gene were sequenced.
- Subsequently, placing bradykinin as the central figure, we investigated the interconnection between the contact, coagulation and complement systems. Using next-generation sequencing, we analyzed 45 genes related to bradykinin synthesis, metabolism, degradation and/or excretion.

**Results:** All index cases were female with the heterozygous c.983C > a variant. In the genetic study of 45 family members, 27 exhibited the heterozygous c.983C > a variant, including 8 men and 19 women, all men being asymptomatic.

Among the 12 families studied with the c.983C > A variant, clinical discrepancies between the index cases and their relatives carrying the c.983C > A variant were observed in three families, prompting an investigation of genetic variations in 45 bradykinin-associated genes to elucidate these symptomatic differences. These were the results:

- Family 6: the variant in the ACE gene, c.1979C > G, present in the symptomatic index patient and absent in the 8 asymptomatic relatives.
- Family 4: the variant in the TPSG1 gene, c.844C > A, present in the index case, with moderate symptomatology, and absent in the family members with mild symptomatology.
- Family 5: two variants in the MASP1, c.1277G > A and XPNPEP2, c.696G > T genes, with a different distribution between the index patient and the relatives.

**Conclusion:** We identified four genes—ACE, TPSG1, MASP1, and XPNPEP2—that may modulate clinical manifestations in HAE-FXII patients. Identifying new genes associated with HAE-FXII is crucial for elucidating the disease's pathophysiology.

## P-28 Quality of life in elderly patients affected by hereditary angioedema: Data from the ITACA register

### 9.1. Francesca Perego^1^, Azzurra Cesoni Marcelli^1^, Riccardo Senter^2^, Federica Ruin^2^, Lorenza Zingale^1^, Antonio Gidaro^3^, Valentina Popescu Janu^3^, Francesco Arcoleo^4^, Pietro Andrea Accardo^4^, Mariangela Lo Pizzo^4^, Andrea Zanichelli^5,6^, Giada De Angeli^7^, Francesco Giardino^8^, Edoardo Cataudella^9^, Alessandra Vultaggio^9^, Andrea Matucci^9^, Angelica Petraroli^10^, Roberta Gatti^10^, Giuseppe Spadaro^10^, Luisa Brussino^11^, Stefania Nicola^11^, Luca Lo Sardo^11^, Maria Domenica Guarino^12^, Mauro Cancian^2^

#### ^1^Department of Internal Medicine, Istituti Clinici Scientifici Maugeri IRCCS, Milan, Italy; ^2^Department of Systems Medicine, University Hospital of Padua, Padua, Italy; ^3^Department of Biomedical and Clinical Sciences, Luigi Sacco Hospital, University of Milan, Milan, Italy; ^4^UOC di Patologia Clinica e Immunologia, Azienda Ospedaliera Ospedali Riuniti Villa Sofia-Cervello, Palermo, Italy; ^5^Department of Biomedical Sciences for Health, University of Milan, Milan, Italy; ^6^Department of Medicine, Angioedema Center, IRCCS Policlinico San Donato, San Donato Milanese, Milan, Italy; ^7^Clinical Research Service, IRCCS Policlinico San Donato, San Donato Milanese, Italy; ^8^A.O.U. Policlinico “G.Rodolico-San Marco”, Catania, Italy; ^9^Immunoallergology Unit, University Hospital of Careggi, Florence, Italy; ^10^Department of Translational Medical Sciences, University of Naples Federico II, Naples, Italy; ^11^Allergy and Clinical Immunology Unit, Department of Medical Sciences, University of Torino & Mauriziano Hospital, Torino, Italy; ^12^Allergy Unit, Hospital of Civitanova Marche, Civitanova Marche, Italy

##### **Correspondence:** Francesca Perego (Francesca.perego@icsmaugeri.it)

***Allergy, Asthma & Clinical Immunology*** 2025, **21(Suppl 2)**:P-28

**Background:** Hereditary angioedema due to C1-inhibitor deficiency (HAE-C1-INH) is a rare disease that affects individuals of all ages [1]. The aging of the European population, particularly in Italy, is one of the fastest among developed countries. It is estimated that in the next 30 years, the proportion of people aged 65 and over will reach one-third of the total population. Patients' needs change over time, and the burden of disease in elderly HAE patients differs from that in younger adults. One of the main tool to evaluate quality of life (QoL) in HAE patients is the AngioEdema QoL questionnaire (AE-QoL) [2,3]. However, the impact of HAE on QoL in the elderly has never been investigated. Aim: to compare QoL in adult HAE-C1-INH patients younger and older than 65 years. Methods and results: data from the ITACA (Italian Network for Hereditary and Acquired Angioedema) Register were prospectively collected from January 1, 2023, to October 30, 2024. A total of 672 adult patients with HAE-C1-INH, including 375 females (55.8%), were enrolled from 10 reference centers. Among them, 114 patients were aged 65 and older (Over 65) (68 females; 58.6%), representing 17.0% of the total adult HAE population. The AE-QoL was completed by 174 patients (31.2%) in the 18–64 age group (HAE 18- 64) and by 31 patients (14.5%) in the Over 65 group (chi-square test n.s., p = 0.71). A total of 241 AE-QoL.

## P-29 Impact of injectable HAE on-demand treatments on health-related quality of life: A patient and caregiver interview study

### Patrick Yong^1,*^, Aleena Banerji^2^, Paula Busse^3^, Timothy Craig^4^, Sorena Kiani-Alikhan^5^, Rebekah Hall^6^, Siu Hing Lo^6^, Caleb Dixon^6^, Paul Audhya^7^, Alice Wang^7^, Tomaz Garcez^8^

#### ^1^Clinical Immunology and Allergy, Frimley Health NHS Foundation Trust, Frimley, UK; ^2^Division of Rheumatology, Allergy and Immunology, Massachusetts General Hospital, Boston, MA, USA; ^3^The Mount Sinai Hospital, New York, NY, USA; ^4^Departments of Medicine, Pediatrics, Ob/Gyn MFM and Biomedical Sciences, The Pennsylvania State University, Hershey, PA, United States, and Vinmec International Hospital, Times City, Hanoi, Vietnam; ^5^Department of Immunology, Royal Free London NHS Foundation Trust, London, UK; ^6^Acaster Lloyd Consulting Ltd, London, UK; ^7^KalVista Pharmaceuticals, Cambridge, Massachusetts, USA; ^8^Department of Immunology, Manchester University NHS Foundation Trust, Manchester, UK

##### **Correspondence:** Patrick Yong (patrick.yong@nhs.net)

***Allergy, Asthma & Clinical Immunology*** 2025, **21(Suppl 2)**:P-29

**Background:** Hereditary angioedema (HAE) attacks can be unpredictable, painful, and debilitating. Limited data has been reported on the burden and impact of approved on-demand therapies on patient and caregiver health-related quality of life (HRQoL). This qualitative study aimed to understand patient and caregiver experiences with injectable on-demand treatments and perceptions of a potential oral on-demand therapy.

**Materials and Methods:** Adult and adolescent patients (≥ 12 years) with ≥ 1 HAE attack in the prior 6 months and caregivers (≥ 18 years) of patients of any age were recruited by patient advocacy organizations Hereditary Angioedema Association (HAEA) in the US and HAE International (HAEi) in the UK to complete HRQoL adaptive qualitative interviews. Questions concerned on-demand treatment use, impact, and burden.

**Results:** Twenty-five (16 from US, 9 from UK) respondents completed the interview (17 patients [12 adults, 5 adolescents]; 8 caregivers); 14 patients/caregivers reported using icatibant as the primary on-demand treatment, while 11 used a C1 esterase inhibitor. 20% (2/10) of adults, who reported using icatibant, and all adolescents had a caregiver or healthcare provider administer on-demand treatments. All respondents reported delaying or forgoing on-demand treatment for attacks, and many respondents (n = 10) associated increased severity and duration of attacks with treatment delays as a result. Respondents commonly reported that lack of portability (n = 13), painful administration (n = 19), and logistical barriers restricting use away from home (n = 19), led to dissatisfaction with their current on-demand treatment. For some participants these characteristics of injectable treatments contributed to poor HRQoL, negative psychological impacts, and activity disruptions or limitations. Respondents indicated that if they had oral on-demand treatment available, they would be willing to treat more attacks (n = 4) and treat them earlier (n = 8).

**Conclusions:** All patients and caregivers described negative HRQoL impacts of injectable on-demand therapy. An oral on-demand option may reduce barriers to treat for patients, and potentially reduce the psychological burden associated with parenteral on-demand treatment use for not only patients living with HAE but also caregivers.

## P-30 Epidemiology of bradykinin-mediated angioedema in the European population

### Emel Aygören-Pürsün^1^, Danny M. Cohn^2^, Henriette Farkas^3^, Sorena Kiani-Alikhan^4^, Markus Magerl^5,6^, Anna Sala-Cunill^7−9^, Andrea Zanichelli^10,11^, Júlia Vila Guilera^12^, Lia Gutierrez^12^, Sabine Ellenberger^13^, Maggie Chen^14^, Joan Mendivil^13^

#### ^1^University Hospital Frankfurt, Goethe University Frankfurt, Frankfurt, Germany; ^2^Amsterdam UMC, University of Amsterdam, Amsterdam, The Netherlands; ^3^Semmelweis University, Budapest, Hungary; ^4^Royal Free London NHS Foundation Trust, London, UK; ^5^Charité-Universitätsmedizin Berlin, Berlin, Germany; ^6^Fraunhofer Institute for Translational Medicine and Pharmacology ITMP, Berlin, Germany; ^7^Hospital Universitari Vall d’Hebron, Barcelona, Spain; ^8^Institut de Recerca Vall d’Hebron (VHIR), Barcelona, Spain; ^9^Universitat Autònoma de Barcelona, Barcelona, Spain; ^10^Universita degli Studi di Milano, Milan, Italy; ^11^IRCCS, UO Medicina, Milan, Italy; ^12^RTI Health Solutions, Barcelona, Spain; ^13^Pharvaris GmbH, Zug, Switzerland; ^14^Pharvaris Inc, Lexington, MA, USA

***Allergy, Asthma & Clinical Immunology*** 2025, **21(Suppl 2)**:P-30

**Rationale:** Angioedema (AE) is a localized, transient swelling of subcutaneous and submucosal tissue mediated by vasoactive compounds, mainly bradykinin or histamine. Angioedema symptoms usually affect upper and lower limbs, face and neck, and genitals, as well as the gastrointestinal and upper respiratory tracts. Involvement of the upper respiratory tract may be life threatening. Bradykinin-mediated AE (AE-BK) comprises two types of AE known to be mediated by bradykinin: hereditary angioedema (HAE) and acquired angioedema (AAE). HAE may be associated with mutations in the C1-inhibitor (C1-INH) protein gene (HAE-C1-INH) and subclassified as HAE-C1-INH Type 1 (deficient C1-INH) or HAE-C1-INH Type 2 (defective C1-INH), or it may be associated with other mutations in patients with normal C1-INH levels and function (HAE-nC1-INH). AAE due to C1-INH deficiency (AAE-C1-INH) is typically secondary to hematologic and immunologic disorders. As a rare disease, data are limited on AE-BK prevalence. This study aimed to summarize epidemiologic data on AE-BK in the European Union (EU) and United Kingdom (UK).

**Methods:** A targeted literature search was performed on 12 June 2024 using MEDLINE (PubMed) and Elsevier Embase databases. The search was designed to identify articles published in peer-reviewed journals that provided population-based epidemiology estimates and/or clinical features of AE-BK in the EU and UK. The search terms were broad to include all types of AE-BK and were limited to human studies published in English from 1 January 2014 to 12 June 2024. All titles and available abstracts provided by the literature search (n = 249) were manually reviewed, and based on prespecified criteria, a subset of articles underwent full-text review (n = 14). Data extraction focused on incidence and/or prevalence estimates for AE-BK in the EU and UK.

**Results:** Data were extracted from 15 articles, including 12 from full-text review and 3 through desktop search. Based on evidence from 12 population-based studies across 10 European countries, the reported prevalence of HAE-C1-INH (Type 1/2) ranged from 0.05/10,000 individuals in Latvia to 0.33/10,000 in Austria. Based on evidence from 2 studies, the reported prevalence of HAE-nC1-INH ranged from < 0.01/10,000 in the UK to 0.07/10,000 in the Canary Islands, Spain. Based on evidence from 3 studies, the reported prevalence of AAE-C1-INH ranged from 0.01/10,000 in the Czech Republic to 0.02/10,000 in Italy.

**Conclusions:** The results of this targeted literature review of 15 articles indicate that AE-BK, including all types, is a rare condition, with prevalence estimates below 5/10,000 individuals.

## P-31 Assessing the diagnostic value of complement testing in type I hereditary angioedema in a pediatric population

### Monica Colque-Bayona^1,*^, Tatiana Navarro-Cascales^1,2,3^, María Bravo García-Morato^2,3,4^, Alberto López-Lera^2,3,5^, Pilar Nozal2^,3,4,5^, Teresa Caballero^1,2,3,5^, Elsa Phillips-Angles^1,2,3^

#### ^1^Department of Allergy, Hospital Universitario La Paz, Madrid, Spain; ^2^La Paz Institute for Health Research (IdiPAZ), Madrid, Spain; ^3^Hospital La Paz National Reference Center (CSUR) for Hereditary Angioedema, Madrid, Spain; ^4^Department of Immunology, Hospital Universitario La Paz, Madrid, Spain; ^5^Center for Biomedical Research Network on Rare Diseases (CIBERER U754), Madrid, Spain

##### **Correspondence:** Monica Colque-Bayona (monica.colque@salud.madrid.org)

***Allergy, Asthma & Clinical Immunology*** 2025, **21(Suppl 2)**:P-31

**Background:** Hereditary angioedema with C1-inhibitor deficiency (HAE-C1-INH) is a rare disease. There is a paucicity of studies on the diagnostic approach in children with suspected disease [1,2]. While confounding results can hamper biochemical diagnosis due to the lower reference levels reported for complement proteins in children as compared to the adult population, genetic testing improves diagnostic reliability.

The aim of this study was to evaluate the accuracy of complement testing for diagnosis of HAE-C1-INH type I in pediatric patients, using SERPING1 gene testing as a reference.

**Material and methods:** We performed a retrospective descriptive study on pediatric patients (< 16 years) suspected of having HAE-C1-INH due to personal or family history. All patients were followed up at the National Reference Centre for Hereditary Angioedema at Hospital Universitario La Paz (Madrid, Spain) between 2004–2024. Demographic data, laboratory findings (C4 levels, C1-INH levels and fC1-INH) and SERPING1 gene testing results were collected. Eight patients without genetic testing were excluded from the study.

**Results:** A total of 61 children were included, 25 were ≤ 1 year [mean: 3.8 months (range 1–12 mo)] and 36 were > 1 year old [mean: 5.3 years (range, 1–18 y.o)].

Patients were categorized into two groups: HAE-C1-INH (positive SERPING1 gene study) with 38 patients (12 aged ≤ 1 year and 26 aged > 1 year), and non-HAE-C1-INH (negative SERPING1 gene study) with 23 patients (13 aged ≤ 1 year and 10 aged > 1 year).

Among patients with HAE-C1-INH aged ≤ 1 year, 66.7% (8/12) had low C1-INH levels [mean 14.2 mg/dL (range 4.9–46.2)], while 91.7% (11/12) had reduced fC1-INH [mean: 30.8% (range 3.6–101.4)]. All non-HAE-C1-INH ≤ 1 year had normal C1-INH levels and function.

In the group of patients with HAE-C1-INH aged > 1 year, C1-INH levels were low in 92.3% (24/26) and fC1-INH levels were low in 96.2% (25/26). In this group, C4 levels were low in 76.9% (20/26) (mean 6.4, 1.5–13.6). On the other hand, in the non-HAE-C1-INH group > 1 year, all patients had normal C1-INH levels and function and C4 levels.

Table 1 summarizes the data on complement testing.

**Table 1 (abstract P-31):** Complement test results

		HAE-C1-INH type I (N = 38)	Non- HAE-C1-INH type I (N = 23)
≤ 1 year (N = 25)	C4 (mg/dL)	7.1 (2.0–12.1)	11.3 (5.5–16.9)
	C1-INH (mg/dL)	14.2 (4.9–46.2)	27.9 (20.7–35.5)
	fC1-INH (%)	30.8 (3.6–101.4)	92.3 (57.7–121.0)
> 1 year (N = 36)	C4 (mg/dL)	6.4 (1.5–13.6)	18.7 (10.9–26.6)
	C1-INH (mg/dL)	9.5 (2.8–22.0)	34.0 (23.2–54.9)
	fC1-INH (%)	23.4 (5.1–55.0)	103.7 (69.7–121.0)

C4 (normal range: 5–30 mg/dL in < 1 year, 9–40 mg/dl in > 1 year), C1-INH (normal range: 16–33 mg/dL); C1-INH function (normal range, > 50%)

Normal ranges of complement in children are not well stablished and could vary

**Conclusion:** Our study found a high level of agreement between SERPING1 gene testing results and functional C1-INH in both patients with HAE-C1-INH type I and those without HAE-C1-INH, regardless of age. However, the agreement was lower between genetic testing and C1-INH levels in children aged ≤ 1 year with HAE-C1-INH type I.

**References**

1. Pedrosa M, Phillips-Angles E, López-Lera A, López-Trascasa M, Caballero T. Complement Study Versus CINH Gene Testing for the Diagnosis of Type I Hereditary Angioedema in Children. J Clin Immunol. 2016; 36(1):16–8.
2. Bocquet A, Pagnier A, Boccon-Gibod I, Defendi F, Dumestre-Perard C, Hardy G, et al. Early diagnosis of hereditary angioedema in children: genetic testing should be prioritized. Allergy Asthma Clin Immunol. 2025; 21(1):8.

## P-32 Survey results from Italy, the US, UK, and France: Anxiety in patients using injectable on-demand treatments for hereditary angioedema attacks

### Mauro Cancian^1^, Alexis Bocquet^2^, Paula J. Busse^3^, Timothy Craig^4,5^, Tariq El-Shanawany^6^, Tomaz Garcez^7^, Padmalal Gurugama^8^, Rashmi Jain^9^, Sorena Kiani-Alikhan^10^, Maeve O’Connor^11,12^, Cristine Radojicic^13^, Sinisa Savic^14^, Paola Triggianese^15^, H. James Wedner^16^, Patrick Yong^17^, Andrea Zanichelli^18,19^, Vibha Desai^20^, Julie Ulloa^21^, Sherry Danese^21^, Paul K. Audhya^20^, Sandra Christiansen^22^

#### ^1^Azienda Ospedale Università di Padova, Padova, Italy; ^2^Grenoble Alpes University Hospital, Grenoble, France; ^3^The Mount Sinai Hospital, New York, NY, USA; ^4^The Pennsylvania State University School of Medicine, University Park, PA, USA; ^5^Allergy Vinmec International Hospital, Times City, Hanoi, Vietnam; ^6^University Hospital of Wales, Cardiff, Wales, UK; ^7^Manchester University NHS Foundation Trust, Manchester, UK; ^8^Cambridge University Hospitals NHS Foundation Trust, Cambridge, UK; ^9^Oxford University Hospitals NHS Foundation Trust, Oxford, UK; ^10^Royal Free London NHS Foundation Trust, London, UK; ^11^Integrative Allergy & Immunology Care Charlotte, NC, USA; ^12^Allergy, Asthma, & Immunology Research Institute, Charlotte, NC, USA; ^13^Duke University School of Medicine, Durham, NC, USA; ^14^The Leeds Institute of Rheumatic and Musculoskeletal Medicine, University of Leeds, Leeds, UK; ^15^Policlinico Universitario Tor Vergata, Department of Biomedicine and Prevention, Rome, Italy; ^16^Washington University School of Medicine, St. Louis, MO, USA; ^17^Frimley Health NHS Foundation Trust, Frimley, UK; ^18^Operative Unit of Medicine, Angioedema Center, IRCCS Policlinico San Donato, San Donato Milanese, Milan, Italy; ^19^Department of Biomedical Sciences for Health, University of Milan, Milan, Italy; ^20^KalVista Pharmaceuticals, Salisbury, United Kingdom, and Cambridge, MA, USA; ^21^Outcomes Insights, Agoura Hills, CA, USA; ^22^University of California San Diego, La Jolla, CA, USA

***Allergy, Asthma & Clinical Immunology*** 2025, **21(Suppl 2)**:P-32

**Background:** Studies report a high prevalence of anxiety among patients with hereditary angioedema (HAE). Currently, all approved on-demand treatments for managing these attacks require parenteral administration, which can be painful and challenging to administer, and may contribute to this anxiety. This study aimed to quantify levels of anxiety associated with use of parenteral on-demand therapy.

**Materials and Methods:** Patients ≥ 12yrs old with Type 1 or Type 2 HAE from the US, UK, France, and Italy were recruited by patient advocacy groups (HAEA, HAE UK, AMSAO) and a physician association (ITACA), respectively, to complete an online survey. Respondents had to have treated ≥ 1 HAE attack with an approved on-demand therapy ≥ 3 months prior to the survey. Participants rated their anxiety using an 11-point GA-NRS ranging from 0 “not anxious” to 10 “extremely anxious” to answer the question “How much anxiety did you feel about treating this HAE attack with on-demand treatment?”.

**Results:** This analysis included 284 respondents (253 adults [≥ 18yrs] and 31 adolescents [range 12-17yrs old]) from Italy (n = 101), US (n = 94), UK (n = 48), and France (n = 41). The mean age (SD) was 41yrs (16.4) and 57% were receiving long-term prophylaxis. Icatibant was the most commonly used on-demand therapy (adults: 68%; adolescents 13%), followed by intravenous (IV) pdC1-INH (adults: 26%; adolescents 65%). During the last treated attack, the mean anxiety rating regarding on-demand treatment was 3.7 (0–10 scale), with 29% of respondents feeling extremely anxious (anxiety 7–10), 17% moderately anxious (anxiety 4–6), 28% mildly anxious (anxiety 1–3), and 26% not anxious (anxiety 0). The mean anxiety rating was 3.5 for adults and 5.3 for adolescents. For respondents receiving IV treatment, the mean anxiety rating was 4.4, compared to 3.3 for those receiving subcutaneous treatment. For respondents treating in < 1 h, the mean anxiety was 3.0. Anxiety increased with increased time to treatment, with mean ratings of 3.6, 3.7, 3.9, and 4.3 when treating within 1 to < 2 h, 2 to < 5 h, 5 to < 8 h, and ≥ 8 h, respectively. The most common reasons for anxiety in participants receiving IV treatment were related to injection and treatment administration (76%). For participants receiving subcutaneous treatment, the most common reasons for anxiety were related to concerns about treatment efficacy (67%).

**Conclusions:** Nearly one third of survey respondents experienced moderate to extreme anxiety due to anticipated use of parenteral on-demand treatment, particularly adolescents and those receiving IV therapy. Greater levels of anxiety were associated with longer on-demand treatment delays.

## P-34 Physician and patient level of agreement in hereditary angioedema attack reporting among a real-world European population

### Mauro Cancian^1^, Gaia A. Luppino^2^, Charlotte Heeks^2^, Madeleine Thursfield^2^, Hannah Connolly^3^, Shiva Lauretta Birija^3^, Ella Morton^3^, Katherine Smethers^3^

#### ^1^Azienda Ospedale-Università di Padova, Italy; ^2^Otsuka Pharmaceutical Europe Ltd, Windsor, UK; ^3^Adelphi Real World, Bollington, UK

***Allergy, Asthma & Clinical Immunology*** 2025, **21(Suppl 2)**:P-34

**Background:** Hereditary angioedema (HAE) is characterized by unpredictable, recurrent angioedema attacks of the extremities, abdomen, and airways. HAE attacks can negatively impact patients’ quality of life and be life threatening. Physician–patient communication is essential to facilitate shared decision-making of effective disease management. This analysis quantifies agreement between physician and patient reported HAE attack frequency and characteristics.

**Methods:** Data were drawn from the the Adelphi HAE Disease Specific Programme™, a real-world single point-in-time survey of physicians and HAE patients in France, Germany, Italy, Spain and the UK between April 2023-January 2024. Correlation analysis as well as weighted and unweighted Cohen’s Kappa were used to measure agreement, from 0.0 (no agreement) to 1.0 (perfect agreement), between matched physician-reported, patient-level data and patient self-reported data. Agreement of frequency, severity and reporting of HAE attacks were measured alongside overall disease severity.

**Results**: Overall, 52 physicians reported on 199 patients with both physician- and patient-reported data. Mean (standard deviation; SD) patient age was 32.2 (12.6, k = 1, perfect agreement) years; 50.3% (k = 1, perfect agreement) were male and 94.5% were HAE-C1-INH-Type1 or HAE-C1-INH-Type2 (k = n/a, HAE type not asked of the patient). Physicians reported 50.8% of patients were prescribed long-term prophylaxis and 75.4% were prescribed on-demand therapy. Physicians and patients were well aligned on the number of attacks experienced in the last 12 months (k = 0.8021, substantial agreement): physicians reported 2.5 (2.2) attacks and patients reported 2.5 (2.2) attacks. Overall, 42.2% of physicians and 39.9% of patients reported ≥ 3 attacks in the last 12 months (k = 0.8536, almost perfect agreement). Additionally, 59.3% of physicians believed patients always reported attacks to their physician, whereas 69.3% of patients reported always “telling” physicians when attacks occurred (k = 0.2953, fair agreement). Patients and physicians showed fair agreement regarding both the current severity of the patient’s overall disease burden (k = 0.3422), and the severity of their most recent attack (k = 0.4030). When reporting attack location, patients (21.4%) and physicians (23.2%) agreed the most regarding abdominal attacks (k = 0.6107, substantial agreement) and had the lowest agreement when reporting laryngeal attacks (k = 0.1550, slight agreement), reported by 9.1% of physicians and 12.0% of patients.

**Conclusions:** Whilst physicians and patients showed substantial agreement on the number of HAE attacks patients experienced each year, there was only fair agreement in how consistently patients reported their HAE attacks and the severity of the HAE attacks patients experienced. Improved communication between physicians and patients could enhance management and overall health outcomes in people with HAE.

## P-35 Physician-reported outcomes versus location of attack in a real-world hereditary angioedema patient population in Europe

### Laurence Bouillet^1^, Gaia A. Luppino^2^, Charlotte Heeks^2^, Madeleine Thursfield^2^, Hannah Connolly^3^, Shiva Lauretta Birija^3^, Ella Morton^3^, Katherine Smethers^3^

#### ^1^National Reference Center for Angioedema (CREAK), Department of Internal Medicine / Clinical Immunology, Grenoble Alpes University Hospital, Grenoble, France; ^2^Otsuka Pharmaceutical Europe Ltd, Windsor, UK; ^3^Adelphi Real World, Bollington, UK

***Allergy, Asthma & Clinical Immunology*** 2025, **21(Suppl 2)**:P-35

**Background:** Hereditary angioedema (HAE) is a genetic disorder manifesting as recurrent and unpredictable angioedema attacks in the peripheral, abdominal or laryngeal regions. HAE attacks can cause severe pain and life-threatening swelling leading to high physical and emotional burden along with lower health-related quality of life (QoL). The aim of this analysis was to explore the relationship between HAE attack location, related symptoms and key disease outcomes.

**Methods:** Data were drawn from the the Adelphi HAE Disease Specific Programme™, a real-world single point-in-time survey of physicians about their patients in France, Germany, Italy, Spain and the United Kingdom between April 2023-January 2024. Physicians reported HAE patient demographics, clinical history, QoL and HAE attack characteristics. Descriptive statistics for patients that experienced ≥ 1 HAE attack in the last 12 months were reported alongside a series of multivariate regressions run against key disease outcomes.

**Results:** Overall, 114 physicians reported on 534 patients with HAE. Patients were 51.5% female, had a mean (standard deviation; SD) age of 32.4 (14.6) years and were 94.2% HAE-C1-INH-Type1 or HAE-C1-INH-Type2. Physicians reported, 49.6% of patients were prescribed long-term prophylaxis and 82.6% were prescribed on-demand treatment. Overall, 88.2% of patient’s most recent HAE attacks were peripheral, 27.3% abdominal or 8.8% laryngeal (non-exclusive). When stratified by anatomical region of most recent HAE attack, the duration in hours was 12.5 (laryngeal), 12.1 (peripheral), and 11.3 (abdominal), respectively.

For patients with laryngeal involvement, at most recent attack, severity, pain and resultant fatigue was reported as severe or very severe (S/VS) in 31.9%, 8.9% and 15.2% respectively. Physicians reported peripheral attacks of S/VS severity (10.9%), S/VS pain (3.8%), and fatigue (3.4%) less frequently than abdominal attacks of S/VS severity (16.4%), S/VS pain (10.6%) and fatigue6.4%. Overall current disease severity was described as S/VS in 17.0% of patients whose most recent attack had laryngeal involvement (peripheral; 8.7% and abdominal; 8.2%). In the last year, HAE-related hospitalisations were reported in 61.1% of patients whose most recent attack had laryngeal involvement (peripheral; 27.4% and abdominal; 34.1%). Physicians-reported patient overall QoL was good or very good in 52.2% of patients whose most recent attack was laryngeal (peripheral; 66.1% and abdominal; 64.4%).

**Conclusions:** Physicians reported patients’ most recent HAE attacks caused severe symptoms, pain and fatigue. This was more prevalent for those whose most recent attack was laryngeal, with increased disease severity. Further research is needed to improve understanding of disease burden in relation to attack location.

## P-36 Distinct SERPING1 variants and genetic modifiers influencing disease expression in hereditary angioedema due to C1-inhibitor deficiency

### Matija Rijavec^1,2,*^, Mitja Košnik^1,3^, Mihaela Zidarn^1,3^, Helena Jakopič^1,3^, Nina Rupar1, Julij Šelb^1,3^, Slađana Andrejević^4^, Ljerka Čulav^5^, Marko Barešić^6^, Vesna Grivčeva-Panovska^7^, Peter Korošec^1,8^

#### ^1^University Clinic of Respiratory and Allergic Diseases Golnik, Golnik, Slovenia; ^2^Biotechnical Faculty, University of Ljubljana, Ljubljana, Slovenia; ^3^Medical Faculty Ljubljana, Slovenia; ^4^Clinic of Allergology and Immunology, University Clinical Center of Serbia, Belgrade, Serbia; ^5^General Hospital Šibenik, Šibenik, Croatia; ^6^University Hospital Center Zagreb, Croatia; ^7^Dermatology Clinic, School of Medicine, Ss. Cyril and Methodius University, Skopje, Republic of Macedonia; ^8^Faculty of Pharmacy, University of Ljubljana, Ljubljana, Slovenia

##### **Correspondence:** Matija Rijavec (matija.rijavec@klinika-golnik.si)

***Allergy, Asthma & Clinical Immunology*** 2025, **21(Suppl 2)**:P-36

**Background:** Hereditary angioedema due to C1-inhibitor deficiency (HAE-C1-INH) is a rare genetic disorder caused by pathogenic variants in the *SERPING1* gene, characterised by recurrent oedema and a highly variable clinical phenotype. The variability of clinical symptoms, both within and between families, suggests that multiple factors influence the disease's expression and penetrance. This study aimed to identify the spectrum of disease-causing variants in *SERPING1* gene among HAE-C1-INH patients and explore novel genetic modifiers contributing to clinical variability.

**Materials and Methods:** We performed a comprehensive genetic analysis of 163 clinically well-characterised HAE-C1-INH patients from 84 unrelated families from Croatia, North Macedonia, Serbia, and Slovenia, which included Sanger and next-generation sequencing of the *SERPING1* gene, along with MLPA, to identify copy number variations. In addition to the primary genetic analysis, We also explored genetic modifiers, focusing on the *F12* gene (NM_000505.4: c.-4 T > C; rs1801020) [1]. Moreover, whole exome sequencing (WES) was performed on symptomatic and asymptomatic family members (three duos), and selected variants were validated in a larger cohort to assess their potential role in disease severity [2].

**Results:** Of the 163 HAE-C1-INH patients, 143 had HAE-C1-INH-Type1 and 20 had HAE-C1-INH-Type2. We identified 55 distinct disease-causing variants in *SERPING1* gene, including 23 missense, 12 nonsense, 9 frameshift, 2 in-frame deletions, 3 splicing defects, 1 promoter substitution, and 5 large insertions/deletions. In two cases, the disease-causing variant remained undetermined. A genotype–phenotype analysis revealed significant differences in the frequencies of the functional *F12* (NM_000505.4: c.-4 T > C; rs1801020) and *CC2D2B* (NM_001159747.2: c.190A > G; rs17383738) variants between symptomatic and asymptomatic patients.

**Conclusions:** This study highlights the genetic heterogeneity of HAE-C1-INH, identifying 55 distinct disease-causing variants in the *SERPING1* gene. Furthermore, we demonstrated the importance of *F12* and *CC2D2B* variants as potential genetic modifiers that may influence disease expression and contribute to the observed clinical variability in HAE-C1-INH. These insights suggest that these variants could be used to differentiate between clinically affected and symptom-free individuals, offering new avenues for understanding and potentially predicting the clinical course of the disease.

**References**

1. Rijavec M, Košnik M, Andrejević S, Karadža-Lapić L, Grivčeva-Panovska V, Korošec P. The functional promoter F12-46C/T variant predicts the asymptomatic phenotype of C1-INH-HAE. Clin Exp Allergy. 2019;49(11):1520-1522.
2. Rupar N, Šelb J, Košnik M, Zidarn M, Andrejević S, Čulav L, Grivčeva-Panovska V, Korošec P, Rijavec M. The CC2D2B is a novel genetic modifier of the clinical phenotype in patients with hereditary angioedema due to C1-inhibitor deficiency. Gene. 2024;919:148496.

## P-37 Bioequivalence of Lanadelumab 300 mg subcutaneous injection administered via the pre-filled syringe and pre-filled pen

### Yi Wang^1^, Richard D. Finkelman^2^, Boris Karolicki^1^, Hong Ren^1^, Irmgard Andresen^3,*^

#### ^1^Takeda Development Center Americas, Inc., Lexington, MA, USA; ^2^Takeda Development Center Americas, Inc., Lexington, MA, USA *at the time of the study*; ^3^Takeda Pharmaceuticals International AG, Zurich, Switzerland

##### **Correspondence:** Irmgard Andresen (irmgard.andresen@takeda.com)

***Allergy, Asthma & Clinical Immunology*** 2025, **21(Suppl 2)**:P-37

**Background:** Lanadelumab is indicated for the routine prevention of recurrent attacks of hereditary angioedema in patients aged ≥ 2 years. Available in a vial or pre-filled syringe, the European Medicines Agency recently approved an additional pre-filled pen option for the administration of Lanadelumab in patients aged ≥ 12 years subcutaneously.

**Methods:** A Phase 1, randomized, open-label, single-dose, parallel-arm, single-centre study (NCT03918239) was conducted in healthy adult volunteers to evaluate the pharmacokinetics (PK), safety, and tolerability of 300 mg of Lanadelumab administered subcutaneously into the abdomen via either the prefilled syringe or prefilled pen. Primary PK parameters included area under the plasma concentration–time curve (AUC) from time zero to the last quantifiable concentration (AUC_0-last_) and extrapolated to infinity (AUC_0-∞_), and maximum observed concentration (C_max_). Ratios (pen:syringe) of the geometric least squares means and 90% CIs were calculated, with bioequivalence concluded if 90% CIs were between 0.8 and 1.25.

**Results:** In total, 190 study participants (aged 18–55 years) were enrolled in parallel (prefilled syringe [n = 94], prefilled pen [n = 96]). The study population included 94 males and 96 females; mean (SD) age was 40.8 (9.91) years and body-mass index was 28.17 (3.226) kg/m^2^; the majority of study participants were White (158/190 [83.2%]). Geometric mean Lanadelumab C_max_ was attained approximately 4 days postdose (geometric mean ratio [90% CIs]: 1.157 [1.044–1.281]); thereafter, plasma concentrations of Lanadelumab declined in a monophasic manner. The geometric mean ratio (90% CIs) was 1.079 (0.986–1.181) for AUC_0-last_ and 1.079 (0.987–1.179) for AUC_0-∞_. Treatment-emergent adverse events reported in ≥ 2 individuals in either group were injection site bruising in 9 (9.4%), back pain in 3 (3.1%), and injection site erythema, thermal burn, anxiety, and insomnia each in 2 individuals (prefilled pen group) and skin abrasion and rash each in 2 participants in the prefilled syringe group. Viral upper respiratory tract infection and headache were reported in 2 (2.1%) participants in both groups. Population PK analyses including data from this single-dose study and predicting PK parameters after repeated subcutaneous administration of Lanadelumab 300 mg every two weeks showed that geometric mean ratios (90% CIs) were 1.001 (0.948–1.056) for AUC over the dosing interval at steady state and 1.029 (0.981–1.081) for C_max_ at steady state.

**Conclusions:** These findings demonstrate bioequivalence of Lanadelumab administered subcutaneously via prefilled pen and prefilled syringe. The prefilled pen provides patients an additional option to the syringe based on individual preferences.

**Trial registration**: Clinicaltrials.gov, NCT03918239

**Funding and acknowledgements** This study was funded by Dyax Corp., a Takeda company.

## P-38 Impact of sex on the course of hereditary angioedema due to C1-inhibitor deficiency

### Hanga Réka Horváth, Lili Voloncs-Mindszenthy, Henriette Farkas

#### Hungarian Angioedema Center of Reference and Excellence, Department of Internal Medicine and Haematology, Semmelweis University, Budapest, Hungary

***Allergy, Asthma & Clinical Immunology*** 2025, **21(Suppl 2)**:P-38

**Background:** Although hereditary angio-edema (HAE) due to C1-inhibitor (C1-INH) deficiency (HAE-C1-INH) is inherited in an autosomal dominant manner, several reports have been published on female predominance in this disease (1, 2). Our aim with the current study was to investigate the impact of the patients’ sex on the course of HAE-C1-INH.

**Patients and methods:** We analysed data of 224 HAE-C1-INH patients currently in our HAE Registry. We analysed patients’ sex distribution using descriptive statistics and inheritance patterns through pedigree analysis. Further, we compared female (F) and male (M) patients’ age at occurrence of their first HAE symptoms and the length of diagnostic delay using the Mann–Whitney U-test; their complement profile at diagnosis (total complement activity, C1q, C3, C4, C1-INH antigenic concentration, C1-INH functional activity) using t-tests; and their disease severity defined as the average yearly attack rates using the Mann–Whitney U-test.

**Results:** In our patient cohort, 54.9% was female and 45.1% male. We observed no significant difference in HAE-C1-INH inheritance rates between female and male offspring, regardless of whether we analysed multi-generational families (occurrence of HAE-C1-INH 53.1% (F) vs. 47.7% (M)) or three-member (mother-father-child) family units constructed from our patient cohort. In the latter case, the inheritance rates were 61.8% (F) vs. 51.2% (M) when the mother had HAE-C1-INH and 43.3% (F) vs. 42.1% (M) when the father had HAE-C1-INH. HAE symptoms occurred earlier in males (13.7 years (F) vs. 12.3 years (M)), but the difference was not statistically significant. Diagnostic delay was shorter in females (9.53 years (F) vs. 10.65 years (M)), but this was also without statistical significance. No statistical significance could be observed in the complement parameters at diagnosis, but male patients tended to have higher levels of each complement parameters. The yearly average of HAE attacks did not differ between the two groups (8.64 (F) vs. 8.31 (M)).

**Conclusions:** In our HAE-C1-INH cohort we observed no statistically significant difference in the inheritance rates, the course of the disease, or the complement parameters at diagnosis between female and male patients. However, male patients tended to have higher complement levels at diagnosis.

**Acknowledgements** The authors are grateful for all HAE-C1-INH patients included in this study for providing their data, and we thank Judit Bali for her invaluable help with data collection from the patients. We would like to express our gratitude to György Sinkovits MD, Andrásné Dóczy, Lászlóné Kertész, and Éva Zsuzsanna Szendrei for their help with complement measurements.

**References**

1. Bork K, Wulff K, Witzke G, Hardt J, Meinke P. Inheritance Pattern of Hereditary Angioedema Indicates Mutation-Dependent Selective Effects During Early Embryonic Development. J Allergy Clin Immunol Pract. 2022;10(4):1029-37.
2. Machhua S, Kumar Jindal A, Basu S, Jangra I, Barman P, Tyagi R, et al. Transmission patterns of C1-INH deficiency hereditary angioedema favors a wild-type male offspring: Our experience at Chandigarh, India. Immunobiology. 2024;229(2):152790.

## P-39 Candidate variants associated with hereditary angioedema with normal C1-Inhibitor

### Helena Jakopič^1,3^, Mitja Košnik^1,3^, Mihaela Zidarn^1,3^, Julij Šelb^1,3^, ^4^, Marko Barešić^6^, Peter Korošec^1,8^, Matija Rijavec^1,2^

#### ^1^University Clinic of Respiratory and Allergic Diseases Golnik, Golnik, Slovenia; ^2^Biotechnical Faculty, University of Ljubljana, Ljubljana, Slovenia; ^3^Medical Faculty Ljubljana, Slovenia; ^4^Clinic of Allergology and Immunology, University Clinical Center of Serbia, Belgrade, Serbia; ^6^University Hospital Center Zagreb, Croatia; ^8^Faculty of Pharmacy, University of Ljubljana, Ljubljana, Slovenia

##### **Correspondence:** Helena Jakopič (helena.jakopic@klinika-golnik.si)

***Allergy, Asthma & Clinical Immunology*** 2025, **21(Suppl 2)**:P-39

**Background:** Hereditary angioedema with normal C1-inhibitor (HAE-nC1-INH) is a rare disorder characterized by recurrent swelling episodes and significant clinical variability. While pathogenic variants in F12, PLG, KNG1, ANGPT1, MYOF, HS3ST6, CPN1, and DAB2IP have been identified in some patients, the genetic basis remains unknown in many cases, suggesting additional contributing factors.

This study aimed to identify new candidate variants associated with HAE-nC1-INH through whole exome sequencing (WES) and analysis of 180 angioedema-related genes, focusing on rare variants.

**Materials and Methods:** We performed WES on 34 HAE-nC1-INH patients without known pathogenic/likely pathogenic variants in causing genes, focusing on 180 angioedema-related genes (GeneCards). Using an in-house bioinformatics pipeline, we aligned sequencing data to the hg19 reference genome and filtered variants, excluding common variants (1000 Genomes), retaining only rare variants with predicted high or moderate functional impact and excluding HLA-DBR gene. Further filtering with gnomAD v2.1.1 (hg19) retained variants with a frequency of ≤ 0.01 in the European (non-Finnish) population. Variant reliability was verified using IGV to exclude sequencing artefacts and false positives. We calculated variant frequencies in our cohort, compared them with gnomAD, and applied a binomial test (Python) to assess statistical significance (p-value < 0.05).

**Results:** Our analysis identified rare variants in 18 genes, including XPNPEP2, BDKRB1, CFH, CFHR2, F5, and ANPEP, as potential contributors to HAE-nC1-INH, being more common among our patients than in the general population (GnomAD2.1.1). XPNPEP2 encodes X-prolyl aminopeptidase 2, an enzyme that degrades bradykinin, a key mediator of vascular permeability, while BDKRB1 encodes the bradykinin B1 receptor, which regulates inflammation and vasodilation. Dysregulated bradykinin metabolism is strongly implicated in HAE. CFH and CFHR2 regulate the complement system, and their disruption can lead to excessive inflammation and increased vascular permeability. F5 (coagulation factor V) plays a central role in blood clotting, while ANPEP, a peptidase, influences the breakdown of bioactive peptides. These findings highlight the interplay between bradykinin metabolism, complement regulation, and coagulation in HAE-nC1-INH pathogenesis.

**Conclusions:** This study identifies potential genetic contributors to HAE-nC1-INH, highlighting genes involved in bradykinin metabolism, complement regulation, and coagulation. These pathways have already been implicated in disease-related genes, and our findings further support their relevance in patients without identifiable genetic alterations. While these results offer valuable insights into the genetic landscape of HAE-nC1-INH, further research is needed to validate these associations and understand their functional significance. Expanding the study to larger cohorts and conducting experimental validation will be crucial to determining their role in disease pathogenesis.

## P-40 Effect of COVID-19 infection on hereditary angioedema (HAE) – survey of Canadian patients

### Amarjot S. Kang, Chrystina Kalicinsky

#### Allergy & Immunology, University of Manitoba, Winnipeg, Canada

***Allergy, Asthma & Clinical Immunology*** 2025, **21(Suppl 2)**:P-40

**Background:** Hereditary angioedema (HAE) is a potentially life-threatening condition characterized by recurrent swelling attacks of the subcutaneous tissue and mucous membranes. COVID-19 is a viral infection with symptomatology that can range from asymptomatic to severe disease with high mortality. The objective of the study is to determine if COVID-19 infection affects the frequency and severity of angioedema attacks in Canadian hereditary angioedema patients and if HAE can predispose to more severe COVID-19 infection.

**Materials & Methods:** A self-reported, anonymous, online questionnaire was distributed to adult members of the patient organization, HAE Canada. Results were collated and analyzed regarding baseline frequency of angioedema attacks, use of long term prophylaxis (LTP), incidence of COVID-19 infection, frequency and severity of angioedema attacks within two weeks of COVID-19 infection, and need for hospitalization with COVID-19 infection.

**Results:** Results from 75 participants were analyzed.

12/75 (16%) male and 63/75 (84%) female. Age range 24–93, mean age of 53 years.

34/75 (45%) of participants identified as Type I HAE, 7/75 (9%) Type II HAE, 26 /75 (35%) HAE nC1-INH, and 8/75 (11%) did not know their HAE type.

23/75 (31%) of participants reported having flares ≥ once per month, 18/75 (24%) once per 3 months, 9/75 (12%) once per 6 months, 21/75 (28%) once per year or less and 4 (5%) non-respondents.

58/75 (77%) of participants were on LTP.

59/75 (79%) of participants tested positive for COVID-19.

6/59 (10%) were admitted to a medical ward and none required admission to ICU.

25 / 59 (42%) reported an angioedema attack within two-weeks of COVID-19 infection.

Attack severity was classified by patient perception as mild, moderate, or severe.

7/25 (28%) reported severe swelling, 14/25 (56%) moderate swelling, 4/25 (16%) mild swelling, within two-weeks of COVID-19 infection.

**Conclusion:** We observed that COVID-19 infection can trigger swelling attacks of moderate to severe intensity. A minority of individuals required hospitalization for COVID-19 infection, similar to the rate in non HAE individuals in Canada.

## P-41 Increased risk of systemic lupus erythematosus in individuals with hereditary angioedema: a family study

### Linda Sundler Björkman^1^, Mirnabi Pirouzifard^2^, Jan Sundquist^2^, Kristina Sundquist^2^, Bengt Zöller^2^

#### ^1^Division of Respiratory medicine, Allergology and Palliative Medicine, Department of Clinical Sciences Lund, Lund University and Skåne University Hospital, Lund, Sweden; ^2^Department of Clinical Sciences Malmö, Center for Primary Health Care Research, Lund University/Region Skåne, Malmö, Sweden

***Allergy, Asthma & Clinical Immunology*** 2025, **21(Suppl 2)**:P-41

**Background:** Hereditary angioedema (HAE) is a rare congenital disease, caused by mutations in SERPING1 gene, leading to deficient or dysfunctional C1-inhibitor (C1-INH). C1-INH is an important inhibitor in the kallikrein-kinin system, intrinsic coagulation pathway, and classical and lectin pathways of the complement system. HAE has been suggested to be associated with an increased risk of systemic lupus erythematosus (SLE). To our knowledge, this is the first national family study of HAE, which aimed to determine the familial risk of SLE.

**Materials and methods:** To evaluate the risk of SLE in patients with HAE, we conducted a national family study of 2006 individuals from 276 pedigrees. Totally, 365 family members had a validated diagnosis of HAE. Multi-Generation Register was linked to the Swedish National Patient Register for the period of 1964 to 2018. Hazard ratios (HRs) and 95% confidence intervals (CIs) for SLE were calculated for patients with HAE in comparison with relatives without HAE. Adjustment was made for sex, year of birth, education, and country of birth.

**Results:** Nineteen (0.9%) family members (18 female, 1 male) were affected by SLE. In total, 12 (3.3%) patients with HAE were affected by SLE, whereas 7 (0.4%) non-HAE relatives were affected (P < 0.001). The overall incidence of SLE in HAE cases was 0.65/1000 person years versus 0.08/1000 person years in non-HAE relatives. The crude HR for SLE among patients with HAE was 8.23 (95% CI, 3.13–21.65, P < 0.001). The adjusted HR for SLE among patients with HAE was 6.14 (95% CI, 2.32–16.23, P < 0.001).

**Conclusions:** HAE is associated with SLE among Swedish families with HAE. We suggest that HAE may be a novel rare genetic cause of SLE. Further studies are needed to investigate the mechanisms by which C1-INH deficiency leads to an increased risk of SLE.

## P-42 CHAPTER-3 phase 3 trial design: Efficacy and safety of the oral bradykinin B2 receptor antagonist deucrictibant extended-release tablet for prophylaxis of hereditary angioedema attacks

### Andrea Zanichelli^1,2^, Henriette Farkas^3^, Anete S. Grumach^4^, Michihiro Hide^5^, Joshua S. Jacobs^6^, Lucy Leeman^7^, Philip H. Li^8^, Ramón Lleonart^9^, Hilary J. Longhurst^10^, William R. Lumry^11^, Markus Magerl^12,13^, Inmaculada Martinez-Saguer^14^, Jonathan Peter^15^, Sinisa Savic^16^, Marcin Stobiecki^17^, Raffi Tachdjian^18^, Anna Valerieva^19^, H. James Wedner^20^, Ricardo Zwiener^21^, Christoph Cirillo^22^, Li Zhu^23^, Ulrich Freudensprung^22^, Joan Mendivil^22^, Ming Yu^23^, Peng Lu^23^, Emel Aygören-Pürsün^24^

#### ^1^Universita degli Studi di Milano, Milan, Italy; ^2^I.R.C.C.S., UO Medicina, Milan, Italy; ^3^Semmelweis University, Budapest, Hungary; ^4^Centro Universitario FMABC, Sao Paulo, Brazil; ^5^Hiroshima University and Hiroshima City Hiroshima Citizens Hospital, Hiroshima, Japan; ^6^Allergy and Asthma Clinical Research, Walnut Creek, CA, USA; ^7^University Hospitals Plymouth NHS Trust, Plymouth, UK; ^8^Queen Mary Hospital, University of Hong Kong, Hong Kong; ^9^Bellvitge University Hospital, Barcelona, Spain; ^10^University of Auckland, Auckland, New Zealand; ^11^Allergy and Asthma Research Associates Research Center, Dallas, TX, USA; ^12^Charité-Universitätsmedizin Berlin, Berlin, Germany; ^13^Fraunhofer Institute for Translational Medicine and Pharmacology, Berlin, Germany; ^14^HZRM Hämophilie Zentrum Rhein Main, Diagnostik, Therapie und Erforschung von Gerinnungsstörungen, Immundefekten und HAE, Frankfurt, Germany; ^15^Groote Schuur Hospital, University of Cape Town, Cape Town, South Africa; ^16^University of Leeds, Leeds, UK; ^17^Jagiellonian University Medical College, Krakow, Poland; ^18^University of California at Los Angeles, Los Angeles, CA, USA; ^19^Medical University of Sofia, University Hospital Alexandrovska, Sofia, Bulgaria; ^20^Washington University School of Medicine, St. Louis, MO, USA; ^21^Hospital Universitario Austral, Buenos Aires, Argentina; ^22^Pharvaris GmbH, Zug, Switzerland; ^23^Pharvaris Inc., Lexington, MA, USA; ^24^University Hospital Frankfurt, Goethe University Frankfurt, Frankfurt, Germany

***Allergy, Asthma & Clinical Immunology*** 2025, **21(Suppl 2)**:P-42

**Rationale:** Deucrictibant is a selective, orally administered antagonist of the bradykinin B2 receptor under development for prophylactic and on-demand treatment of hereditary angioedema (HAE) attacks. In the placebo-controlled CHAPTER-1 Phase 2 trial (NCT05047185), deucrictibant significantly reduced occurrence of attacks, induced clinically meaningful improvement in control of disease and in health-related quality of life, and was well tolerated. In Phase 1 studies, deucrictibant extended release (XR) tablet resulted in a sustained mean level in circulation exceeding the threshold of therapeutic exposure (effective concentration that leads to 85% maximal response, EC_85_, 13.8 ng/mL) from 1.5 to at least 24 h post-dose.

**Methods:** CHAPTER-3 (NCT06669754) is an ongoing Phase 3, multicenter, randomized, double-blind, placebo-controlled study designed to evaluate the efficacy and safety of orally administered deucrictibant XR tablet once daily for prophylaxis of HAE attacks. Participants must be ≥ 12 years old and diagnosed with HAE. Eligible participants are randomized in a 2:1 ratio to receive 40 mg deucrictibant XR tablet or matching placebo once daily for 24 weeks.

**Results:** The primary efficacy endpoint is time-normalized (per 4 weeks) number of investigator-confirmed HAE attacks during the 24-week treatment period. Secondary efficacy endpoints include attacks treated with on-demand medication, rate of ‘moderate and severe’ attacks, and proportion of participants achieving ≥ 50%, ≥ 70%, ≥ 90% attack rate reduction or remaining attack free. Patient-reported outcomes (PROs) include the evaluation of health-related quality of life (HRQoL) using the Angioedema Quality of Life questionnaire (AE-QoL) and of disease control using the Angioedema Control Test (AECT). Safety measures include treatment-emergent adverse events (TEAEs), serious TEAEs, and changes in clinical laboratory tests, ECG, and vital signs including blood pressure. After CHAPTER-3 completion, participants can continue treatment with deucrictibant XR tablet in an open-label extension study.

**Conclusion:** CHAPTER-3 is an ongoing, global, Phase 3 study designed to evaluate the efficacy and safety of once daily, oral deucrictibant XR tablet for prophylaxis of attacks in adolescents and adults with HAE.

## P-43 Genetic features of bradykinin-forming cascade and vascular endothelium permeability regulation pathways in Brazilian families with recurrent angioedema

### Clarissa A. Bittencourt^1,2^, Raquel L. Neves^1^, Beatriz R. Nogueira^1^, Marcelo Y. Icimoto^1^, Caio P. Gomes^1^, Agatha R. Mendes^1^, Camila L. Veronez^1^, Michael Bader^2^, João B. Pesquero^1^

#### ^1^Department of Biophysics, Federal University of São Paulo (UNIFESP), São Paulo, Brazil; ^2^Max-Delbrück Center for Molecular Medicine (MDC), Berlin, Germany

***Allergy, Asthma & Clinical Immunology*** 2025, **21(Suppl 2)**:P-43

Angioedema (AE) is characterized by swelling of the subcutaneous and submucosal tissues due to increased vascular permeability. Hereditary angioedema (HAE) is a rare, often underdiagnosed and potentially life-threatening genetic disorder marked by recurrent AE episodes, primarily resulting from dysregulation of the bradykinin-forming cascade. HAE is caused by pathogenic variants in *SERPING1*, leading to C1-inhibitor deficiency (HAE C1-INH), and in *F12, ANGPT1, PLG, KNG1, MYOF, HS3ST6, DAB2IP*, and *CPN1*, which are associated with HAE with normal C1-INH (HAE nC1-INH). However, in many individuals with HAE nC1-INH, the underlying pathomechanism remains unknown. This study aimed to investigate the genetic profile of components involved in the bradykinin-forming cascade and vascular endothelial permeability regulation in individuals with HAE symptoms. Molecular analyses were performed using Sanger sequencing, multiplex ligation-dependent probe amplification (MLPA), and whole-exome sequencing (WES). Among 53 individuals from 23 HAE C1-INH families, 20 different pathogenic or likely pathogenic variants were identified in *SERPING1*, predominantly missense and nonsense variants, including three novel variants reported for the first time. Additionally, the pathogenic variant p.Thr328Lys in *F12* was detected in 61 individuals from 40 HAE nC1-INH families. No pathogenic variants were identified in exon 9 of *F12* in 80 probands. Clinical manifestations within our cohort were variable, with a predominance of symptomatic female patients, especially in HAE-FXII. Stress and trauma were the most common triggers, while hormonal influences were reported as exacerbating factors in female patients with both HAE C1-INH and HAE nC1-INH. Furthermore, WES identified potentially disease-causing variants in *CPN1, HS3ST6, KNG1, NOS3,* and *PLG*, suggesting their possible role in HAE nC1-INH. Computational modeling of *NOS3* variants predicted structural alterations in the encoded protein. To further investigate the functional impact of variants in ANGPT1 and NOS3, site-directed mutagenesis was performed, and ongoing studies involve transient transfection of HEK293 cells for functional analysis. Our findings underscore the genetic heterogeneity of *SERPING1* variants and reveal that approximately 30% of HAE nC1-INH families carry a pathogenic variant affecting FXII function, while most remain without a confirmed genetic diagnosis. Expanding the understanding of AE attack modulation mechanisms and identifying new genetic variants could enhance HAE pathophysiology knowledge, facilitate early diagnosis, enable targeted therapies, and improve genetic counseling for affected families.

## P-44 Utility of abdominal ultrasound in the diagnosis of abdominal angioedema

### Gabriela Leon-Zambrana^1^, Fiorella Adrianzen^1^, Joaquín Poza^2^, Marta Goyanes-Malumbres^1,3,4,5^

#### ^1^Allergy Department, Hospital Universitario La Paz, Madrid, Spain; ^2^Gastroenterology Department, Hospital Universitario La Paz, Madrid, Spain; ^3^Hospital La Paz Institute for Health Research (IdiPAZ), Madrid, Spain; ^4^CSUR de Angioedema Hereditario, Hospital Universitario La Paz, Madrid, Spain; ^5^Center for Biomedical Research Network on Rare Diseases (CIBERER U754), Madrid, Spain

***Allergy, Asthma & Clinical Immunology*** 2025, **21(Suppl 2)**:P-44

**Introduction:** Angioedema is characterized by recurrent cutaneous, subcutaneous, and submucosal swellings due to increased vascular permeability. When it affects the gastrointestinal (GI) tract, it often presents with abdominal pain, mimicking other clinical conditions, which can lead to misdiagnosis and inadequate treatment.

We present three cases of angioedema -histaminergic, hereditary, and acquired- highlighting the role of abdominal ultrasound (AUS) as a key diagnostic tool.

**Case 1—Hereditary angioedema:** A 41-year-old male with hereditary angioedema due to C1-inhibitor deficiency (HAE-C1-INH) without long-term prophylaxis (LTP) experienced an abdominal attack over two days. Initially, the patient reported mild pain (VAS 1/10). He self-administered icatibant 30 mg subcutaneously (SC), with adequate symptom control. However, the following day, the pain worsened (VAS 7.5/10), accompanied by nausea and uncontrollable vomiting. AUS revealed marked mucosal edema with Kerckring folds (KF) in the colon and jejunum, with moderate ascites. He was treated with intravenous (IV) C1-inhibitor (1,000 IU), leading to symptom resolution.

**Case 2—Acquired angioedema:** A 46-year-old male with acquired angioedema due to C1-inhibitor deficiency (AAE-C1-INH) under LTP with danazol (100 mg/day) developed abdominal pain after having lunch. He had presented oral lesions suggestive of herpes simplex days before. The pain worsened throughout the day, reaching a VAS score of 10/10. He self-administered icatibant 30 mg SC, resulting in partial symptom relief (VAS 4/10) within 30 min. AUS detected ascites in the pelvis and abdomen, as well as ileal mucosal edema with prominent KF. The patient received IV C1-inhibitor (1,500 IU), but pain persisted for four hours before complete resolution two days later.

**Case 3—Histaminergic angioedema:** A 37-year-old female with a history of histaminergic angioedema presented with abdominal pain and loose stools, without other symptoms or signs of peritoneal irritation.

AUS revealed free perihepatic, perisplenic, and pelvic fluid. The patient improved with H1-antihistamines. After the episode, LTP with H1-antihistamines was initiated, and she experienced no recurrent episodes.

**Conclusions:** The identification of mucosal edema (KF) and ascites, makes AUS a fast and useful diagnostic tool in emergency settings. However, there were no specific findings that suggest that it may be helpful to differentiate between angioedema subtypes. Furthermore, it is important to consider that AUS is an operator-dependent test. Even so, it may aid in the early recognition of abdominal angioedema, thereby preventing misdiagnosis, unnecessary surgical interventions, and inappropriate treatments. Its non-invasive nature makes it a valuable first-line tool in patients with suspected abdominal angioedema. Consent to publish had been obtained.

## P-45 Phenotype and SERPING1 genotype of a cohort of 46 unrelated families with HAE-C1-INH in Southern Spain

### Krasimira Baynova^1^, Teresa De Aramburu Mera^1^, José Manuel Lucena^2^, Raúl García^2^, Isabel Fernández de Alba Porcel^3^, Stefan Cimbollek^1^

#### ^1^Allergy Department, Spanish National Center for Angioedema, Virgen del Rocío University Hospital, Seville, Spain; ^2^Immunology Department, Spanish National Center for Angioedema, Virgen del Rocío University Hospital, Seville, Spain; ^3^HLA Inmaculada University Hospital, Granada, Spain

***Allergy, Asthma & Clinical Immunology*** 2025, **21(Suppl 2)**:P-45

**Background:** HAE-C1-INH is a low-prevalence genetic disease with an autosomal dominant mode of inheritance. The activity of the disease is highly variable from one patient to another and even in the same patient throughout lifetime. Our objective was to compare the phenotype and SERPING1 genotype of a cohort of 106 HAE-C1-INH patients from 46 unrelated families who receive regular follow-up at our reference center.

**Materials and methods:** 49 males, 57 females, with a mean age of 42.15 years ( 6 months-88 years) were inclueded. 43 families presented HAE-C1-INH type 1 and 3 families-HAE-C1-INH type 2. Details regarding clinical expression were documented. Patients and progenitors of paediatric patients were informed about the genetic testing and signed informed consent.

The severity of the disease was assessed and mild, moderate, severe and very severe phenotype were established according to:

- annual frequency of attacks
- severity of attacks
- disease control measured through angioedema control test (AECT)
- disease-related quality of life measured through Angioedema Quality of Life questionnaire (AEQoL)
- need of first-line Long Term Prophilaxis at usual or higher doses

**Results:** The disease severity phenotypes were distributed as follows: mild -24.53% (n26), moderate -21.7%( n23), severe -26.42%( n28) and very severe 18.87%( 20). 9 patients(8.49%) remained asymptomatic, 7 of them were children under 14 years.

Testing of the SERPING1 gene found casual variants in all families in heterozygosity. 41 unique causal variants were found, including 11 previously undescribed ones. Missense variants were the most common 45.65%( n21,), followed by frameshift 19.57%(n9), nonsense17.36% (n = 8), splice defect 8.7% (n4), large deletions 6.52% (n3,) and a single no stop variant (2.17%).

Distributing the phenotypes among the different variants in SERPING1, the following relationships were found:

- genetic variants with protein changes (large deletions, nonsense, frameshift, no stop)- 65 patients: very severe phenotype- 24.62%(n16), severe phenotype-30.77%(n20), moderate phenotype-15.38% (n10) and mild phenotype- 21.54%(n14). 5 patients (7.69%) remained asymptomatic.
- genetic variants that predict amino acid change (missense)-41 patients who presented as follows: very severe phenotype-9.76%( n4), severe -19.51%( n 8), moderate phenotype-29.27% (n12), mild phenotype- 31.71%( n13). 4 patients( 9,76%) remained asymptomatic.

**Conclusions:** We found 11 novel variants in SERPING1. In our cohort of patients a genotype that causes protein changes, presented almost twice more (55.39%) severe and very severe phenotypes compared to subjects with genotype that causes amino acid change (29.18%).

## P-46 Genetic insights in pediatric hereditary angioedema: Two cases treated with Lanadelumab

### Rolando Laurel Laurel^1^, Francisco Alberto Contreras Verduzco^2^, Emmanuel Arce Estrada^3^, Sandra Nieto^4^

#### ^1^Instituto Nacional de Pediatría, Dirección de Investigación, Mexico City, Mexico; ^2^Instituto Nacional de Pediatría, Servicio de Alergia, Mexico City, Mexico; ^3^Instituto Nacional de Pediatría, Unidad de Investigación en Inmunodeficiencias, Mexico City, Mexico; ^4^Instituto Nacional de Pediatría, Unidad de Genética de la Nutrición, Mexico City, Mexico

***Allergy, Asthma & Clinical Immunology*** 2025, **21(Suppl 2)**:P-46

**Background:** Hereditary angioedema (HAE) is a rare autosomal dominant disorder with a prevalence of 0.9 cases per 50,000 individuals in Mexico as of 2019.^2^ Historically, management focused on symptom control, with prophylaxis primarily using 17-alpha-alkylated androgens, which, while effective, often caused significant adverse effects, especially in women and children. Advances in understanding HAE-C1-INH have led to targeted therapies for both acute attacks and prevention. Current management includes on-demand therapy, short-term prophylaxis before procedures, and long-term prophylaxis for severe or recurrent cases.^3^ Most cases of HAE-C1-INH are caused by mutations in the SERPING1 gene, which encodes C1-inhibitor (C1-INH), resulting in reduced or dysfunctional C1-INH and uncontrolled vascular permeability.^4^ Other genetic variants, including mutations in F12, PLG, and ANGPT1, have been linked to HAE-nC1-INH.1 The relationship between genetic variants and treatment response remains an area of ongoing research, particularly in pediatric patients.

**Case report:** We present two pediatric cases with genetic insights, treated with Lanadelumab, showing improvements in quality of life (QoL).

**Case 1:** A 14-year-old male with a history of debilitating angioedema since age 3 was diagnosed with HAE-C1-INH at age 8. Genetic testing in 2022 revealed a pathogenic SERPING1 variant (c.1081C > T p.(Gln361*)), linked to decreased C1-INH production. Despite various treatments, including tranexamic acid and pdC1-INH, he experienced up to 4 attacks/month. Long-term prophylaxis with Lanadelumab led to a significant reduction in attack frequency and severity, improving his quality of life (Figure 1 shows the QoL questionnaire results).

**Figure:**
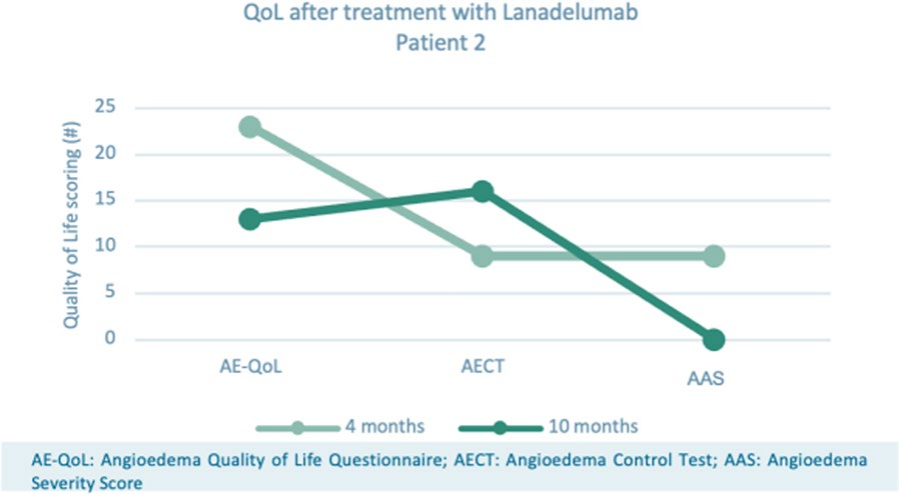
**Figure 1 (abstract P-46)**

**Case 2:** A 13-year-old female with a maternal history of angioedema started experiencing attacks at age 6. Two months before admission, she reported a sensation of a foreign body in her throat and dyspnea, without respiratory distress. Treated with icatibant and tranexamic acid, genetic testing in 2019 revealed the SERPING1 variant c.508_509delinsAA p.(Ser170Asn). Despite treatment, she continued to experience frequent episodes, prompting a switch to long-term prophylaxis with Lanadelumab, leading to a significant reduction in attacks and improved quality of life (Figure 2 shows the QoL questionnaire results).

**Figure:**
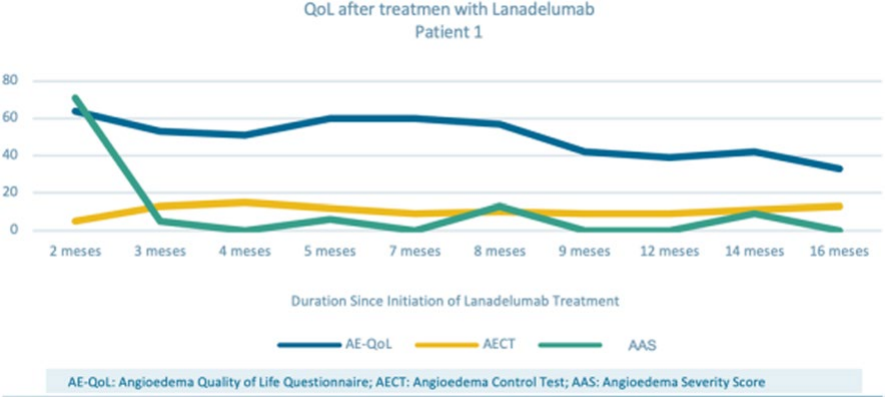
**Figure 2 (abstract P-46)**

**Conclusion:** Genetic testing played a crucial role in tailoring the treatment approach for these patients. Although Lanadelumab’s use in pediatrics is still limited and there are no validated assessment tools, these cases highlight the importance of prioritizing its use, especially in patients with confirmed genetic mutations.

The reduction in attack frequency and improved quality of life observed supports the efficacy of Lanadelumab and emphasizes the need for genetic testing to guide treatment decisions. Consent to publish had been obtained.

**References**

1. Sinnathamby ES. Hereditary Angioedema: Diagnosis, Clinical Implications, and Pathophysiology. Adv Ther. 2023.
2. Nieto et al. World Allergy Organization Journal (2023) 16:100812 10.1016/j.waojou.2023.100812
3. Zafra H. Hereditary Angioedema: A Review. WMJ. 2022 Apr;121(1):48-53. PMID: 35442579.
4. Maurer M, Lumry WR, et.al. Lanadelumab in Patients 2 to Less Than 12 Years Old With Hereditary Angioedema: Results From the Phase 3 SPRING Study. J Allergy Clin Immunol Pract. 2024 Jan;12(1):201-211.e6. 10.1016/j.jaip.2023.09.009. Epub 2023 Sep 18. PMID: 37730089.

## P-47 An update on SERPING1 variants identified in Hungarian patients with hereditary angioedema, including patients with two alterations and novel variants

### Ágnes Szilágyi^1^, Edina Szabó^1^, Katalin Marossy^1^, Hanga Horváth^2^, Henriette Farkas^2^

#### ^1^Research Laboratory, Department of Internal Medicine and Haematology, Semmelweis University, Budapest, Hungary; ^2^Hungarian Angioedema Center of Reference and Excellence, Department of Internal Medicine and Haematology, Semmelweis University, Budapest, Hungary

***Allergy, Asthma & Clinical Immunology*** 2025, **21(Suppl 2)**:P-47

**Background:** Hereditary angioedema (HAE) with C1-inhibitor (C1-INH) deficiency is an autosomal dominant disorder caused by variants in the SERPING1 gene. The underlying genetic causes identified in Hungarian HAE-C1-INH patients have been previously reported [1], here we present the SERPING1 variants identified in nine newly diagnosed families.

**Methods:** HAE-C1-INH was diagnosed based on complement measurements, while SERPING1 was screened using bidirectional sequencing following PCR amplification and multiplex ligation-dependent probe amplification.

**Results:** Despite the fact that the disease is inherited in an autosomal dominant manner, two SERPING1 variants were detected in two HAE-C1-INH families. Three affected individuals from a kindred carried two previously unreported missense changes, p.T452I and p.G453V. These nearby variants were in a cis configuration based on familial inheritance, suggesting that either one or both together may be responsible for the disease. In contrast, the other patient with two variants carried an assumed de novo, previously characterized pathogenic variant, p.P498L, along with another missense change, p.S255T, which had been reported previously in a single HAE-C1-INH case. The index patient’s unaffected father and uncle, both of whom had normal C1-inhibitor levels and function, also carried the p.S255T change indicating that this variant is in fact benign in the context of C1-inhibitor deficiency. Two novel disease-causing variants leading to the generation of premature stop codons (p.Y199Pfs*57, p.S137X) were identified in two other families. In one patient multiplex ligation-dependent probe amplification revealed a large heterozygous deletion involving exons 4–8 of the SERPING1 gene. Additionally, previously reported SERPING1 variants (c.-22-19_-22-4del, c.51 + 2 T > C, p.Q348X, p.R466C) were detected in the remaining four families.

**Conclusions:** By combining routinely used molecular genetic approaches, such as sequencing of the SERPING1 gene (including exons, exon/intron boundaries, and untranslated regions) along with copy number analysis of exons, all the underlying disease-causing variants could be identified in each family with HAE-C1-INH. In addition, our study highlights the importance of screening unaffected family members alongside affected individuals, as this strategy can help assess the pathogenicity of a variant even in cases where functional characterization is unavailable.

**References**

1. Edina Szabó, Dorottya Csuka, Noémi Andrási, Lilian Varga, Henriette Farkas, Ágnes Szilágyi. Overview of SERPING1 Variations Identified in Hungarian Patients With Hereditary Angioedema. Front Allergy. 2022; 3:836465.

## P-48 Atypical presentation of hereditary angioedema: A case of HS3ST6 mutation with uncommon symptoms and normal C1-INH levels

### Sandra Nieto^1,*^, Eugenia Vargas Camaño^2^, Francisco Alberto Contreras Verduzco^3^, Rolando Laurel Laurel^4^

#### ^1^Instituto Nacional de Pediatría, Unidad de Genética de la Nutrición, Mexico City, Mexico; ^2^CMN 20 de Noviembre ISSSTE, Mexico City, Mexico; ^3^Instituto Nacional de Pediatría, Servicio de Alergia, Mexico City, Mexico; ^4^Instituto Nacional de Pediatría, Dirección de Investigación, Mexico City, Mexico

##### **Correspondence:** Sandra Nieto (sandranietoaeh@gmail.com)

***Allergy, Asthma & Clinical Immunology*** 2025, **21(Suppl 2)**:P-48

**Background:** Hereditary angioedema (HAE) is an autosomal dominant disorder caused by mutations in the C1 esterase inhibitor (C1-INH) gene, affecting approximately 1 in 50,000 people. There are three main types: Type I (C1-INH deficiency), Type II (dysfunctional C1-INH), and HAE with normal C1-INH activity (HAE-nC1-INH). HAE-nC1-INH is subdivided based on genetic mutations, including HS3ST6 (c.536 T > A, p.Val179Glu), identified as likely pathogenic[2][3]. Symptoms often appear before 20 and are triggered by stress, infections, or trauma. Lab findings show abnormal C1-INH levels, elevated bradykinin, and low C4 levels. Treatment includes C1-INH infusions, receptor antagonists, and kallikrein inhibitors, along with prophylaxis to prevent attacks[1].

**Case Report:** We present the case of a pediatric patient with a family history that includes a father with osteochondromatosis, an 11-year-old sister with arthralgia currently under investigation, and a maternal aunt with lupus. She has a history of recurrent arthralgia, abdominal pain, and angioedema. The patient initially presented symptoms at 1.5 years of age, with intermittent knee arthralgia. In 2020, following a tonsillectomy, she experienced a severe laryngospasm that required intensive care. In 2022, she was hospitalized for abdominal pain, fever, facial edema, and arthralgia, initially treated as an allergic reaction, with partial response to antihistamines and glucocorticoids. In each of these events, epistaxis was identified as a prodromal symptom. Additionally, C1 esterase inhibitor levels and the percentage of C1-INH function and complement testing were normal. Genetic testing (February 2024) identified a HS3ST6 c.536 T > A p.Val179Glu N/A heterozygous variant, classified as likely pathogenic and associated with HAE-nC1-INH. In February 2024, she presented to the emergency department with colicky abdominal pain, fever (38 °C), productive cough lasting 27 days, and nasal congestion. She had previously been prescribed ibuprofen for suspected pharyngitis, with no improvement. Given her history and current symptoms, a diagnosis of HAE was considered. (Fig. 1: Family tree illustrating possible HAE inheritance.)

**Figure 1 (abstract P-48):**
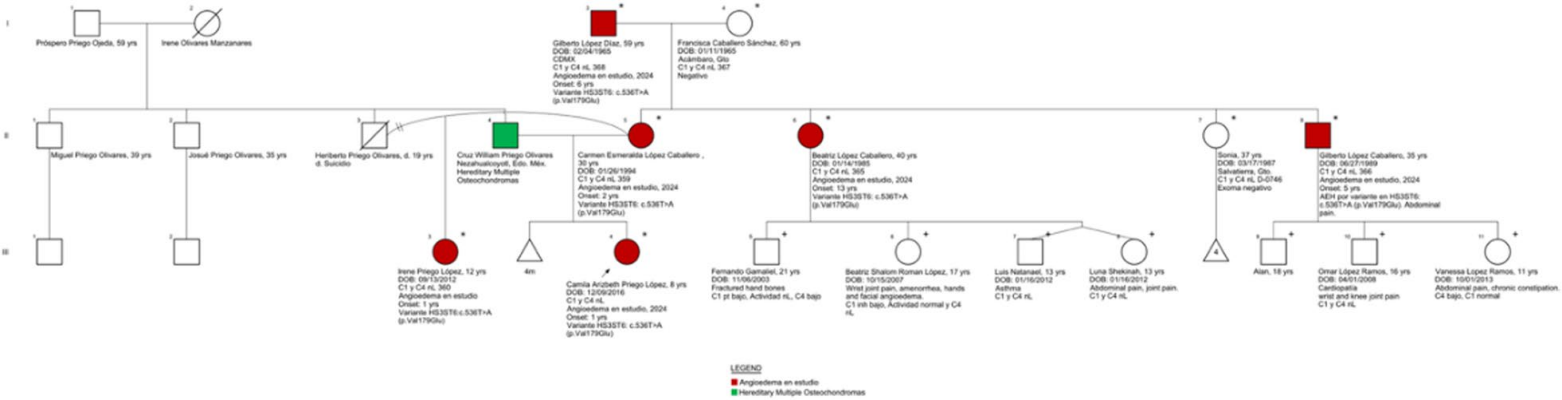
Family tree illustrating possible HAE inheritance

**Conclusion:** This case highlights the clinical variability of HAE, particularly in newly described genetic variants. The HS3ST6 c.536 T > A p.Val179Glu N/A, heterozygous mutation may contribute to atypical HAE presentations, including arthralgia and prolonged respiratory symptoms. Early recognition and genetic testing are crucial for accurate diagnosis and appropriate management, preventing unnecessary treatments for alternative diagnoses such as autoimmune disorders. Consent to publish had been obtained.

**References**

1. Sinnathamby ES. Hereditary Angioedema: Diagnosis, Clinical Implications, and Pathophysiology. Adv Ther. 2023.
2. Germenis AE, Speletas M. Genetics of Hereditary Angioedema Revisited. Clin Rev Allergy Immunol. 2016 Oct;51(2):170-82. 10.1007/s12016-016-8543-x. PMID: 27116602
3. Bork K, Wulff K, Möhl BS, Steinmüller-Magin L, Witzke G, Hardt J, Meinke P. Novel hereditary angioedema linked with a heparan sulfate 3-O-sulfotransferase 6 gene mutation. J Allergy Clin Immunol. 2021 Oct;148(4):1041-1048. 10.1016/j.jaci.2021.01.011. Epub 2021 Jan 25. PMID: 33508266

## P-49 First report of hereditary angioedema due to C1-inhibitor deficiency in Bosnia and Herzegovina: Novel mutation and evidence of genotype–phenotype association

### Mensuda Hasanhodzic^1^, Azra Lukavackic^1^, Aneta Vareskic^1^, Matija Rijavec^2^

#### ^1^University Clinical Centre Tuzla, Centre for Rare Diseases and Medical Gitenetics, Tuzla, Bosnia and Herzegovina; ^2^University Clinic of Respiratory and Allergic Diseases Golnik, Golnik, Slovenia

***Allergy, Asthma & Clinical Immunology*** 2025, **21(Suppl 2)**:P-49

Hereditary angioedema (HAE) is a rare autosomal dominant, potentially life-threatening genetic disease characterized by subcutaneous and submucosal swelling of the face, oropharynx, larynx, genitals, or extremities, associated with intra-abdominal edema and pain. HAE is caused by mutations in the SERPING1 gene, which encodes C1-inhibitor (C1 INH). The gene mutation causes low levels of C1 INH (HAE type 1) or normal levels of ineffective C1 INH (HAE type 2). Our study identified HAE type I in five unrelated families with 10 individuals in the Federation of Bosnia and Herzegovina. The diagnosis of HAE was made in the presence of clinical symptoms and laboratory criteria (low levels of C1-inhibitor antigen and/or function, low level of C4 complement), accompanied by a positive family history. One patient (1/10) had no family history. Genetic studies were performed using PCR and sequencing to detect SERPING1 mutations in the promoter, non-coding exon 1, 7 coding exons, and exonintron boundaries. Multiplex ligation-dependent probe amplification was performed to search for large deletions/duplications in the SERPING1 gene. Nine patients from four families with HAE have been identified as having a mutation responsible for HAE, while genetic analysis of one patient from a fifth family is still ongoing. This study identified two previously reported intronic splice variants of the SERPING1 gene c.889 + 1G > A (family 1) and c.-22-2A > T (one individual from the family 3), and one missense known variant c.1477G > A (p.Gly493Arg) (family 2). One novel variant, c.1996C > T (p.Pro377Leu) is identified in the large BosnianMontenegrin family. In conclusion: Our study identified five families and four different mutations in the HAE population of Bosnia and Herzegovina, confirming the heterogeneity of mutations in the SERPING1 gene that cause C1 INH HAE in the human population. Further analyses will help in faster detection of HAE patients in Bosnia and Herzegovina, especially asymptomatic familial cases.

## P-50 Hereditary angioedema breakthrough: FXII mutation discovery and the power of Icatibant and Lanadelumab

### Aviv Talmon, Limor Rubin, Yaarit Ribak, Oded Shamriz, Mariana Druker, Inon Sarig, Eyal Ben Dori, Yuval Tal

#### Allergy and Clinical Immunology Unit, Department of Medicine, Hadassah Medical Organization, Faculty of Medicine, Hebrew University of Jerusalem, Israel

***Allergy, Asthma & Clinical Immunology*** 2025, **21(Suppl 2)**:P-50

**Background, Objectives:** Hereditary angio-edema (HAE) is a rare, life-threatening disorder characterized by recurrent angioedema episodes. We delve into the less explored domain of Normal C1-inhibitor-HAE caused by FXII mutations. Our study highlights the efficacy of acute and prophylactic interventions for FXII-HAE management.

**Methods:** Exome sequencing was conducted on the index patient, which revealed the presence of an FXII mutation (c.983C > A, p.Thr328Lys). Subsequently, we conducted genetic investigation involving 28 out of 76 family members, utilizing Sanger sequencing.

Additionally, genetic testing was performed on a 53-year-old female patient who had been under suspicion of having HAE but had been misdiagnosed with Familial Mediterranean Fever (FMF) for 40 years.

**Results:** Out of 28 family members in the study, 18 were FXII mutation carriers- seven asymptomatic males, seven females with clinic of estrogen-triggered angioedema episodes and four asymptomatic pre-pubertal girls indicating a risk for angioedema during their first ovulation.

The index patient was treated successfully with Icatibant. Subsequent prophylactic Lanadelumab therapy completely prevented her attacks, which had previously occurred 3–4 times per month.

The case study revealed a novel FXII gene mutation (c530T > C, p.Val177Ala). A CT scan during an acute attack confirmed extensive intestinal angioedema. Icatibant treatment aborted the attack, and prophylactic Lanadelumab therapy significantly reduced angioedema episode frequency.

**Conclusions:** Icatibant and Lanadelumab treatments have shown significant effectiveness in managing HAE caused by FXII mutations. Our research has reclassified a previously labeled "VOUS" mutation in the FXII gene as pathogenic, enhancing our understanding of FXII HAE.

Early genetic screening is crucial for timely monitoring and intervention.

## P-51 Development of a WB-based method to quantify different forms of C1-INH in HAE-C1-INH-Type2, based on the example of the R444C variant

### Bence Farkas^1^, Péter Gál^1^, Lilian Varga^2^, Henriette Farkas^2^, József Dobó^1,*^

#### ^1^Institute of Molecular Life Sciences, HUN-REN Research Centre for Natural Sciences, Budapest, Hungary; ^2^Hungarian Angioedema Center of Reference and Excellence, Department of Internal Medicine and Haematology, Semmelweis University, Budapest, Hungary

##### **Correspondence:** József Dobó (dobo.jozsef@ttk.hu)

***Allergy, Asthma & Clinical Immunology*** 2025, **21(Suppl 2)**:P-51

We have shown earlier that the dominant form of the C1-inhibitor (C1-INH) R444C variant is its disulfide linked complex to serum albumin. This variant is the most common among patients with hereditary angioedema type 2 (HAE-C1-INH-Type2) in Hungary. We identified that patients carrying this mutation contain both albumin-complexed and monomeric C1-INH R444C, beside wild-type monomeric C1-INH. We have developed a Western blot (WB)-based method to quantify these different forms from patient plasma samples. The method involved a one-step purification on Jacalin-agarose, and treatment with recombinant C1r catalytic (CCP1-CCP2-SP) fragment prior to electrophoresis (non-reducing SDS-PAGE) and WB. Our data showed that patient #1 had ~ 57% (~ 72% by weight) C1-INH-R444C-albumin complex, while the remaining uncomplexed fraction comprised ~ 20% (~ 13% by weight) monomeric R444C variant and ~ 23% (~ 15% by weight) wild-type C1-INH. Our initial findings demonstrated the viability of this approach, however, the results showed variation between the different isolates from the same patient. These factors motivated us to refine the method for improved robustness.

To overcome the above mentioned problem, we initially attempted to use whole plasma samples to avoid protein loss during purification, and test different direct blotting strategies, like using Cy5 fluorophore-labeled primary antibody, or employing peroxidase-conjugated primary antibody for chemiluminescent detection. However, both approaches had issues, particularly in terms of signal linearity and transfer efficiency (whole plasma), prompting us to reconsider the one-step purification-based protocol. We optimized the isolation to virtually eliminate loss of C1-INH in the flow-through and wash fractions, ensuring that we recovered essentially all C1-INH in the eluate. Further optimization involved the use of gradient gels, which ensured the greater separation of different C1-INH forms and enhanced transfer in larger molecular ranges, allowing for their simultaneous detection. Our revised approach focuses exclusively on protease-treated samples, ensuring that all three forms are detected in a single analytical step. To achieve this, we tested different fragments of recombinant target proteases, identifying a smaller C1r (CCP2-SP) catalytic fragment as the most promising candidate. This fragment provides the best analytical performance for distinguishing and quantifying the different forms of C1-INH associated with HAE-C1-INH-Type2, particularly in the context of the R444C mutation.

## P-52 Uncovering urinary protein biomarkers for early diagnosis and evaluation of hereditary angioedema

### Jianqing Wu^1^, Nan Zhou^2^, Yuxiang Zhi^2,*^

#### ^1^State Key Laboratory of Complex Severe and Rare Diseases, Peking Union Medical College Hospital, Chinese Academy of Medical Sciences and Peking Union Medical College, Beijing, China; ^2^Department of Allergy & Clinical Immunology, National Clinical Research Center for Immunologic Diseases, Peking Union Medical College Hospital, Chinese Academy of Medical Sciences and Peking Union Medical College, Beijing, China

##### **Correspondence:** Yuxiang Zhi (Yuxiang_zhi@126.com)

***Allergy, Asthma & Clinical Immunology*** 2025, **21(Suppl 2)**:P-52

**Purpose:** Hereditary angioedema (HAE) is a rare genetic disorder characterized by recurrent episodes of severe swelling, often affecting the skin, gastrointestinal tract, and airway. This study aimed to identify urinary protein biomarkers specific to HAE, elucidate their roles in disease pathogenesis, and evaluate their potential for noninvasive clinical monitoring.

**Methods:** Urinary proteomics was performed using data-independent acquisition (DIA) and parallel reaction monitoring (PRM) techniques on samples from HAE patients and healthy controls. Differentially expressed proteins were identified and analyzed for biological pathway involvement using bioinformatics tools. Enzyme-linked immunosorbent assays (ELISA) were performed to validate the levels of several key proteins in an independent cohort. Correlations between urinary protein levels and disease severity were assessed using a scoring system based on clinical and laboratory parameters.

**Results:** Our study identified 269 differentially expressed urinary proteins (203 upregulated, 66 downregulated) in HAE patients compared to healthy controls. IPA analysis revealed that immune-inflammatory pathways (e.g., IL-15 signaling, complement system, leukocyte extravasation) and biological processes related to vascular permeability, endothelial dysfunction, and immune dysregulation were significantly enriched in HAE. The STRING- and Cytoscape-based co-expression network identified 10 hub proteins, among which the biomarker panel (C1-INH, EGF, KNG1) demonstrated robust diagnostic performance (AUC = 0.956). Moreover, it was validated in two independent clinical cohorts, with area under the curve (AUC) values of 0.910 and 0.949 for HAE diagnosis. Additionally, some urinary protein levels were significantly correlated with the disease severity of HAE.

**Discussion:** This study provides novel insights into the urinary proteome of HAE patients, uncovering critical biological pathways and identifying promising biomarkers. The findings advance our understanding of HAE pathogenesis and may contribute to the development of noninvasive diagnostic and monitoring tools for HAE in clinical settings.

## P-53 Proactive identification of rare diseases: Lessons from hereditary angioedema diagnosis using electronic medical records

### Xue Wang^1^, Huizhen Jiang^2^, Ziyang Huang^2^, Chao Dong^2^, Weiguo Zhu^3,*^, Shuyang Zhang^4,**^, Yuxiang Zhi^1,***^

#### ^1^Department of Allergy & Clinical Immunology, Peking Union Medical College Hospital, Peking Union Medical College & Chinese Academy of Medical Sciences, National Clinical Research Center for Immunologic Diseases, Beijing, China; ^2^Department of Information Center, Peking Union Medical College Hospital, Peking Union Medical College, Chinese Academy of Medical Sciences, Beijing, China; ^3^Department of Primary Care and Family Medicine, State Key Laboratory of Complex Severe and Rare Diseases, Peking Union Medical College Hospital, Chinese Academy of Medical Sciences and Peking Union Medical College, Beijing, China; ^4^Department of Cardiology, Peking Union Medical College Hospital, Chinese Academy of Medical Sciences and Peking Union Medical College, Beijing, China

##### **Correspondence:** Weiguo Zhu (zhuwg@pumch.cn); Shuyang Zhang (shuyangzhang103@nrdrs.org); Yuxiang Zhi (yuxiang_zhi@126.com)

***Allergy, Asthma & Clinical Immunology*** 2025, **21(Suppl 2)**:P-53

**Background:** Diagnosing rare diseases traditionally requires patients to endure lengthy and challenging journeys to find specialists familiar with their conditions. This study advocates a paradigm shift in rare disease diagnosis, moving from patients seeking physicians to physicians actively identifying patients.

**Method:** Using hereditary angioedema (HAE) as an example, we demonstrate how this approach, supported by electronic medical records (EMRs), enables proactive care for patients with rare diseases. Our EMR system incorporates a free-text search engine to screen for patients with potential HAE based on clinical symptoms and laboratory tests. Search terms include recurrent skin edema, abdominal pain, laryngeal edema, and/or decreased C4 levels. Suspected cases are followed up by telephone calls from trained physicians, inviting patients to undergo confirmatory C1-INH and C4 testing and genetic testing to ensure accurate diagnosis and appropriate treatment.

**Results:** Of 2,689 patients who met the screening criteria, 3,441 records were analyzed. Ninety-five patients had already been diagnosed with HAE. After excluding those with a known etiology for edema or characteristics inconsistent with HAE, three patients with unexplained cutaneous edema, abdominal pain, and/or laryngeal edema were included in the final screening. Laboratory tests confirmed HAE in all three, highlighting the effectiveness of this proactive approach.

**Conclusions:** This study underscores the transformative potential of EMRs in diagnosing rare diseases. By shifting the responsibility of identifying rare diseases from patients to healthcare professionals, we expedite diagnosis and exemplify the spirit of service in medicine, ensuring patients with rare diseases receive timely and effective care.

**Competing interests: **The authors declare that they have no competing interests.

**Keywords** Proactive diagnosis, Electronic medical records, Hereditary angioedema, Rare diseases

## P-54 Factors influencing quality of life in angioedema due to C1-inhibitor deficiency: A single-center experience

### Riccardo Senter, Federica Ruin, Mauro Cancian

#### Azienda Ospedale-Università di Padova, Padova, Italy

***Allergy, Asthma & Clinical Immunology*** 2025, **21(Suppl 2)**:P-54

**Background:** Quality of life (QoL) questionnaires have been extensively used to assess the efficacy of treatments for angioedema due to C1-inhibitor (C1-INH) deficiency, comparing pre-treatment and post-treatment time points in the same patient. When completed by multiple patients simultaneously, they can also provide an estimate of the overall quality of life of the entire population. The AE-QoL questionnaire [1] was specifically developed for angioedema patients and explores four different domains: food, fatigue/mood, fear/shame, and functioning. Additionally, it provides a global score. The aim of our study was to assess the AE-QoL scores in patients at our center and to identify the factors influencing them.

**Methods:** We conducted a single-center, cross-sectional evaluation of patients with angioedema due to C1-inhibitor deficiency (both hereditary and acquired), asking all patients to complete the AE-QoL questionnaire during their annual visit. The questionnaire was completed through a dedicated section of our national registry (ITACA Registry), which was approved by our ethics committee in 2017. All patients who completed the questionnaire between January 1, 2024, and December 31, 2024, were included in the study.

**Results:** A total of 74 patients were recruited: 66 with hereditary angioedema (HAE) and 8 with acquired angioedema (AAE) due to C1-INH deficiency. Among them, 44 were female (59.45%), and the mean ± standard deviation age was 46.7 ± 17.9 years.

Neither the global AE-QoL score nor the domain-specific scores differed significantly by gender or diagnosis. Patients on long-term prophylaxis (LTP) had significantly better scores in the functioning domain (p = 0.048), but no significant differences were observed in other domains or in the global score. Patients experiencing attacks had significantly worse scores in the functioning and fear/shame domains (p < 0,001 and 0,022 respectively), as well as in the global score. (p = 0,009).

In regression analysis, the number of attacks, the number of severe attacks, and the presence of attacks involving the airways (but not a history of laryngeal attacks) predicted worse global and domain-specific AE-QoL scores. Age of onset and age at diagnosis were not correlated with a worse global score. However, age itself tended to predict a worse global score.

**Conclusion:** Our study showed that the number and severity of attacks, as well as airway involvement, are the primary factors impairing quality of life, emphasizing the importance of addressing these aspects in clinical practice. The benefit of long-term prophylaxis appears to be primarily due to its ability to reduce the frequency and severity of attacks.

**References**

1. Weller K, Groffik A, Magerl M, Tohme N, Martus P, Krause K, Metz M, Staubach P, Maurer M. Development and construct validation of the angioedema quality of life questionnaire. Allergy. 2012 Oct;67(10):1289-98.

## P-55 Safety and efficacy of oral deucrictibant for treatment of upper airway and laryngeal hereditary angioedema attacks: Results from the RAPIDe-2 extension study

### Ramón Lleonart Bellfill^1^, John Anderson^2^, Emel Aygören-Pürsün^3^, Laurence Bouillet^4^, Hugo Chapdelaine^5^, Henriette Farkas^6^, Delphine Gobert^7^, Roman Hakl^8^, Joshua S. Jacobs^9^, Michael E. Manning^10^, Avner Reshef^11^, Giuseppe Spadaro^12^, Maria Staevska^13^, Marcin Stobiecki^14^, Anna Valerieva^13^, Justin Sun^15^, Yumeng Li^15^, Ming Yu^15^, Giorgio Giannattasio^16^, Peng Lu^15^, Marc A. Riedl^17^

#### ^1^Bellvitge University Hospital, L'Hospitalet de Llobregat, Barcelona, Spain; ^2^AllerVieHealth, Birmingham, AL, USA; ^3^University Hospital Frankfurt, Goethe University Frankfurt, Frankfurt, Germany; ^4^Grenoble Alpes University, Grenoble, France; ^5^CHU de Montréal, Université de Montréal, Montréal, QC, Canada; ^6^Semmelweis University, Budapest, Hungary; ^7^Sorbonne Université, Hôpital Saint-Antoine, Paris, France; ^8^Masaryk University, Brno, Czech Republic; ^9^Allergy and Asthma Clinical Research, Walnut Creek, CA, USA; ^10^Allergy, Asthma and Immunology Associates, Ltd., Scottsdale, AZ, USA; ^11^Barzilai University Hospital, Ashkelon, Israel; ^12^University of Naples Federico II, Napoli, Italy; ^13^Medical University of Sofia, Sofia, Bulgaria; ^14^Jagiellonian University Medical College, Krakow, Poland; ^15^Pharvaris Inc., Lexington, MA, USA; ^16^Pharvaris GmbH, Zug, Switzerland; ^17^University of California San Diego, La Jolla, CA, USA

***Allergy, Asthma & Clinical Immunology*** 2025, **21(Suppl 2)**:P-55

**Rationale:** Hereditary angioedema (HAE) attacks are caused by excess bradykinin activating bradykinin B2 receptors and are potentially life-threatening when airways, including the larynx, are involved. Deucrictibant is a selective, orally administered bradykinin B2 receptor antagonist under development for prophylactic and on-demand treatment of HAE attacks. This analysis investigated the safety and efficacy of deucrictibant immediate-release (IR) capsule for on-demand treatment of upper airway, including laryngeal, HAE attacks.

**Methods:** RAPIDe-2 (NCT05396105) is an ongoing, two-part, Phase 2/3 extension study evaluating the long-term safety and efficacy of deucrictibant IR capsule for on-demand treatment of HAE attacks. RAPIDe-2 Part A enrolled adult participants who completed the Phase 2, placebo-controlled RAPIDe-1 trial (NCT04618211). These participants continued to self-administer the same double-blinded dose of deucrictibant IR capsule (10 mg, 20 mg, or 30 mg) received during RAPIDe-1 to treat qualifying attacks including laryngeal attacks presenting without breathing difficulties. Investigators confirmed upper airways, including laryngeal, attacks as per the protocol definition of the presence of swelling of the lips or tongue or any sensation of lump in the throat, difficulty swallowing, or voice change. For upper airway attacks with inadequate response ≥ 4 h after first dose of deucrictibant, only rescue medication was permitted. For other attacks, a second dose of deucrictibant was permitted.

**Results:** This Part A data snapshot (cutoff: 31 May 2024) included 335 attacks treated with deucrictibant IR capsule, of which 7 met the study definition of an airway attack. Airway attacks were experienced by 5 participants. The cohort of 19 participants analyzed had a total of 328 non-airway attacks. For participants with or without airway attacks, deucrictibant IR capsule was well tolerated with no treatment-related treatment emergent adverse events (TEAEs) reported. As measured using the Patient Global Impression of Change (PGI-C), the median (95% CIs) time to onset of symptom relief for airway attacks and total non-airway attacks was 0.9 (0.5, 2.0) and 1.1 (1.0, 1.1) hours. Using PGI of Severity (PGI-S), the median time to substantial symptom relief was 3.0 (0.9, not estimable) and 2.7 (2.1, 2.9) hours, respectively. 85.7% (6/7) of upper airway attacks did not use rescue medication within 48 h post-treatment. 85.4% (280/328) of non-upper airway attacks were treated with a single dose of deucrictibant.

**Conclusions:** In Part A of the RAPIDe-2 study, safety and efficacy outcomes of treatment with deucrictibant IR capsule were consistent for both HAE attacks in the upper airway (including laryngeal attacks) and HAE attacks in other locations.

## P-56 Durability of response to a single dose of oral deucrictibant for on-demand treatment of hereditary angioedema attacks

### Anna Valerieva^1^, John Anderson^2^, Emel Aygören-Pürsün^3^, Maria Luisa Baeza^4^, Laurence Bouillet^5^, Hugo Chapdelaine^6^, Danny M. Cohn^7^, Aurélie Du-Thanh^8^, Olivier Fain^9^, Henriette Farkas^10^, Delphine Gobert^11^ Jens Greve^12^, Mar Guilarte^13^, David Hagin^14^, Roman Hakl^15^, Joshua S. Jacobs^16^, Aharon Kessel^17^, Sorena Kiani-Alikhan^18^, Pavlína Králíčková^19^, H.Henry Li^20^, Ramón Lleonart^21^, Markus Magerl^22,23^, Michael E. Manning^24^, Avner Reshef^25^, Bruce Ritchie^26^, Giuseppe Spadaro^27^, Maria Staevska^1^, Petra Staubach^28^, Marcin Stobiecki^29^, Gordon L. Sussman^30^, Michael D. Tarzi^31^, William H. Yang^32^, Yumeng Li^33^, Li Zhu^33^, Ming Yu^33^, Peng Lu^33^, Umar Katbeh^34^, Giorgio Giannattasio^34^, Marc A. Riedl^35^

#### ^1^Medical University of Sofia, Sofia, Bulgaria; ^2^AllerVie Health, Birmingham, AL, USA; ^3^University Hospital Frankfurt, Goethe University Frankfurt, Frankfurt, Germany; ^4^Hospital General Universitario Gregorio Marañón, Gregorio Marañón, Spain; ^5^Grenoble Alpes University, Grenoble, France; ^6^CHU de Montréal, Université de Montréal, Montréal, QC, Canada; ^7^Amsterdam UMC, University of Amsterdam, Amsterdam, The Netherlands; ^8^University Montpellier, Montpellier, France; ^9^Sorbonne University, AP-HP, Saint Antoine Hospital, Paris, France; ^10^Semmelweis University, Budapest, Hungary; ^11^Sorbonne Université, AP-HP, Saint Antoine Hospital, Paris, France; ^12^Ulm University Medical Center, Ulm, Germany; ^13^Hospital Universitari Vall d'Hebron, Barcelona, Spain; ^14^Allergy and Clinical Immunology Unit, Tel Aviv, Israel; ^15^Masaryk University, Brno, Czech Republic; ^16^Allergy and Asthma Clinical Research, Walnut Creek, CA, USA; ^17^Technion-Israel Institute of Technology, Haifa, Israel; ^18^Royal Free London NHS Foundation Trust, London, UK; ^19^University Hospital Hradec Kralove, Charles University, Hradec Kralove, Czech Republic; ^20^Institute for Asthma and Allergy, Chevy Chase, MD, USA; ^21^Bellvitge University Hospital, L'Hospitalet de Llobregat, Barcelona, Spain; ^22^Charité-Universitätsmedizin Berlin, Berlin, Germany; ^23^Fraunhofer Institute for Translational Medicine and Pharmacology ITMP, Berlin, Germany; ^24^Allergy, Asthma and Immunology Associates, Ltd., Scottsdale, AZ, USA; ^25^Barzilai University Hospital, Ashkelon, Israel; ^26^University of Alberta, Edmonton, AB, Canada; ^27^University of Naples Federico II, Napoli, Italy; ^28^University Medicine Mainz, Mainz, Germany; ^29^Jagiellonian University Medical College, Krakow, Poland; ^30^University of Toronto, Toronto, ON, Canada; ^31^University Hospitals Sussex NHS Foundation Trust, Brighton, UK; ^32^University of Ottowa, Ottawa, ON, Canada; ^33^Pharvaris Inc., Lexington, MA, USA; ^34^Pharvaris GmbH, Zug, Switzerland; ^35^University of California San Diego, Division of Allergy and Immunology, La Jolla, CA, USA

***Allergy, Asthma & Clinical Immunology*** 2025, **21(Suppl 2)**:P-56

**Rationale:** Hereditary angioedema (HAE) attacks are caused by excess bradykinin activating bradykinin B2 receptors. Deucrictibant is an orally administered bradykinin B2 receptor antagonist under development for prophylactic and on-demand treatment of HAE attacks. For people with HAE experiencing an attack, a rapid and sustained response to on-demand treatment through to complete resolution is paramount to abate the physical, functional, and emotional burden associated with the symptoms and to enable the prompt restart of daily activities. We assessed the durability of effects following a single dose of deucrictibant immediate-release (IR) capsule for treatment of HAE attacks.

**Methods:** RAPIDe-1 (NCT04618211) was a double-blind, placebo-controlled, crossover, phase 2 trial evaluating the efficacy and safety of deucrictibant IR capsule (10 mg, 20 mg, or 30 mg) for treatment of HAE attacks. After RAPIDe-1, participants could enrol in the two-part, Phase 2/3 RAPIDe-2 (NCT05396105) extension study. In RAPIDe-2 Part A, participants self-administered the same double-blinded dose of deucrictibant IR capsule. In a post-hoc analysis of both studies, durability of response to a single dose of deucrictibant IR capsule was evaluated as the achievement and maintenance of serial milestones of symptom relief and resolution without reoccurrence of symptoms during the period specified for each outcome (12 or 24 h).

**Results:** In RAPIDe-1, 80/96 (83.3%) attacks were treated with a single dose of deucrictibant across all dose groups (10 mg: 75.7%; 20 mg: 89.3%; 30 mg: 87.1%). Of the 78/80 attacks that reached onset of symptom relief (≥ 30% reduction in visual analogue scale [VAS]-3 score) by 24 h, 97.4% maintained a durable response. Of the 70/80 attacks that achieved almost complete or complete attack resolution (all 3 VAS item scores ≤ 10) by 24 h, 98.6% had durable effects and no subsequent worsening of symptoms. In RAPIDe-2, 395/465 (84.9%) attacks were treated with a single dose of deucrictibant across all dose groups. Of the 385/395 attacks that achieved onset of symptom relief (“a little better” on the Patient Global Impression of Change [PGI-C]) by 12 h, 99.2% had a durable response. Of the 350/395 attacks that achieved complete attack resolution (“none” on the PGI-Severity [PGI-S]) by 24 h, 98.3% had durable effects and no evidence of symptoms reoccurring.

**Conclusions:** In a post-hoc analysis of Phase 2 studies, response to a single dose of deucrictibant IR capsule was durable and majority of attacks achieved symptom relief and resolution without reoccurrence of symptoms.

## P-57 DEFICIT-syndrome, a novel entity in cerebral vasogenic oedema

### Simon Péter Nagy^1^, Sarolta Dobner^2^, Fanni Tóth-Szumutku^2^, Léna Szabó^2^, Ádám Goschler^2^, Anikó Szabó^3^, Georgina Szabó-Fehér^4^, Melinda Zombor^4^, Alíz Zimmerman^4^, Dániel Jakab^4^, Csaba Bereczki^4^, Zoltán Prohászka^1^, Ágnes Szilágyi^1^

#### ^1^Department of Internal Medicine and Hematology, Research Laboratory, Semmelweis University, Budapest, Hungary; ^2^Pediatric Center, Tűzoltó street Department, Semmelweis University, Budapest, Hungary; ^3^Albert Szent-Györgyi Health Centre, Department of Rheumatology and Immunology, University of Szeged, Szeged, Hungary; ^4^Albert Szent-Györgyi Health Centre, Department of Pediatrics and Pediatric Health Center, University of Szeged, Szeged, Hungary

***Allergy, Asthma & Clinical Immunology*** 2025, **21(Suppl 2)**:P-57

Deficiency of factor I with cerebral inflammation (DEFICIT-syndrome), is a recently identified disease, associated with complete complement factor I deficiency and characterized by recurrent aseptic encephalitis/meningitis and oedema. This condition, like hereditary angioedema (HAE), results from dysregulation of the complement system. However, it exhibits a key distinction in its primary involvement of the alternative complement pathway. Given that both conditions may present with central nervous system oedema, it is crucial to consider both disorders. Therefore, we report two pediatric cases of DEFICIT-syndrome, diagnosed within our Research Laboratory at Semmelweis University, to illustrate key diagnostic features.

An 8-year-old boy, previously treated with severe pleuropneumonia, was hospitalized with progressive cerebral vasogenic oedema, necessitating ICU treatment, decompressive craniotomy and plasmapheresis. Despite extensive testing, no infectious agent was identified. Given the medical history and the severe ongoing immunmediated inflam-mation, a complement-mediated disease was suspected. In the acute sample the classical pathway activity was deficient, followed by rapid normalization. C1q, C4 and C1-inhibitor levels were in the reference range. Additional complement analysis revealed consistently low C3 level, deficient alternative pathway activity and elevated terminal complex level. Laboratory findings confirmed deficient Factor I level in the index patient. Furthermore, decreased protein levels were found in the father, mother, and sibling. Genetic investigations demonstrated that the child inherited two distinct rare CFI variants, maternally and paternally, resulting in reduced Factor I level.

The other patient is a 15-year-old girl with one-year history of recurrent severe headaches, nuchal rigidity and vasogenic oedema correlating with her menstrual cycles. Repeated cerebrospinal fluid (CSF) examinations failed to identify a definitive pathogenic organism. Her complement profile showed signs of alternative pathway dysregulation with low Factor I level and normal C1q, C4 and C1-inhibitor concentrations. In this case, we likewise identified two pathogenic rare variants in the CFI gene associated with Factor I deficiency.

When evaluating patients with CNS oedema of unclear origin or vasogenic cerebral oedema without identifable infectious or causative agent, it is imperative to include DEFICIT-syndrome in the differential diagnosis alongside HAE, and to conduct complete complement analysis and genetic investigations to achieve the correct diagnosis. Consent to publish had been obtained.

## P-58 Hereditary angioedema (HAE) and post traumatic stress disorder (PTSD)

### Sandra C. Christiansen^1^, D.G. Baker^2^, Bruce L. Zuraw^1,3^

#### ^1^Division of Allergy and Immunology, Department of Medicine, University of California, San Diego, USA; ^2^Center of Excellence for Stress and Mental Health, VASDHS, San Diego, CA 92161, and Department of Psychiatry, UC San Diego, La Jolla, CA, USA; ^3^Research Service, San Diego VA Healthcare System, USA

***Allergy, Asthma & Clinical Immunology*** 2025, **21(Suppl 2)**:P-58

**Background:** HAE patients may be vulnerable to the development of PTSD as a consequence of repeated unpredictable trauma associated with attacks of disfiguring swelling, severe abdominal pain, risk of asphyxiation or experiences with loss of a loved one with HAE.

**Methods:** Two cohorts of participants 18 and older were recruited from the United States Hereditary Angioedema Association (US HAEA) with a documented history of HAE due to C1-inhibitor deficiency (HAE-C1-INH): Cohort 1, self-referred; Cohort 2, randomized from the HAEA membership roster. Subjects completed 8 on-line questionnaires in a defined sequence within 4 weeks. These were: 1) HAE survey; 2) SF-36 Quality of Life (QOL); 3) Childhood Trauma Questionnaire (CTQ); 4) Beck Depression Inventory-II (BDI-II); 5) Life Events Checklist form for HAE (LEC-HAE); 6) PTSD checklist-civilian version (PCL-C); 7) Peritraumatic Dissociative Experiences Questionnaire (PDEQ) and 8) Peritraumatic Distress Inventory (PDI).

**Results:** The prevalence of presumptive PTSD based on the PLC-C outcomes was between 20–25% in HAE-C1-INH participants (n = 77) with no significant difference between the self-referred (n = 29) and randomized (n = 48) cohorts. Significant correlations were found between the PLC-C scores and BDI II scores (p < 0.0001, R2 = 0.23), total CTQ scores (p = 0.001, R2 = 0.13), PDEQ scores (p < 0.0001, R2 = 0.23) and PDI scores (p < 0.0001, R2 = 0.39). SF-36 total mental health scores were significantly associated with presumptive PTSD at all PLC-C cut-off scores as were the mental health subdomains (vitality, social functioning, role-emotional, and mental health). Early age of HAE symptom onset as well as a history of requiring emergency department visits for HAE attacks were both significantly associated with presumptive PTSD.

**Conclusion:** Our study identified a high prevalence of presumptive PTSD among HAE-C1-INH subjects. Indication of a PTSD diagnosis was correlated with an increased disease burden with high rates of depression and reduced functioning for SF-36 QOL mental domains. The interrelationship between PTSD and HAE may represent reciprocal risk factors enhancing disease severity and offer new strategies for treatment.

**Funding** US HAEA Association.

